# Molecular detection of *bla_VIM_* and *bla_NDM_* in multidrug-resistant *Pseudomonas aeruginosa* from cancer and burn patients in Erbil, Iraq

**DOI:** 10.3389/fmicb.2025.1672531

**Published:** 2025-09-15

**Authors:** Bahra Kakamin Hamad, Muayad Ahmed Mahmud

**Affiliations:** ^1^Medical Laboratory Technology Department, Erbil Health and Medical Technical College, Erbil Polytechnic University, Erbil, Iraq; ^2^Medical Laboratory Technology Department, Shaqlawa Technical College, Erbil Polytechnic University, Erbil, Iraq

**Keywords:** *Pseudomonas aeruginosa*, multidrug resistance, carbapenem resistance, carbapenemase, *bla_VIM_*, *bla_NDM_*, cancer, burn

## Abstract

**Introduction:**

*Pseudomonas aeruginosa* is an opportunistic Gram-negative pathogen and a critical-priority organism according to the World Health Organization. Its increasing resistance to multiple antimicrobial classes, including carbapenems, poses a major challenge in treating infections among immunocompromised individuals, particularly burn and cancer patients.

**Methods:**

This cross-sectional study investigated phenotypic resistance profiles, carbapenemase classifications using an advanced expert system, and the molecular prevalence of *bla_VIM_* and *bla_NDM_* genes in 50 clinical isolates from cancer, burn, and other immunocompromised patients in Erbil, Iraq.

**Results:**

Multidrug resistance and carbapenem resistance were detected in 66.0 and 58.0% of isolates, respectively, with the highest burden among burn patients (93.8%). Carbapenem resistance was significantly associated with prior carbapenem exposure (*p* = 0.0044) and increased mortality (p = 0.0392). Carbapenemase-producing isolates classified by the advanced expert system exhibited universal multidrug resistance and more than 95% resistance to imipenem and meropenem. Molecular analysis identified *bla_VIM_* in 47.5%, *bla_NDM_* in 10.0%, and both genes in 30.0% of tested isolates, with *bla_NDM_* significantly associated with carbapenem resistance (*p* = 0.027). Resistance patterns varied by patient group and antibiotic class, with burn isolates demonstrating the highest rates.

**Discussion/conclusion:**

These findings highlight the need for enhanced molecular surveillance, infection control, and antimicrobial stewardship in high-risk settings.

## 1 Introduction

*Pseudomonas aeruginosa* is a metabolically versatile, Gram-negative opportunistic pathogen that poses a major global healthcare threat due to its intrinsic resistance mechanisms and remarkable capacity to acquire additional resistance determinants. It thrives in nutrient-limited environments and hospital settings and is a leading cause of healthcare-associated infections (HAIs), particularly in immunocompromised individuals such as burn and cancer patients or those undergoing transplantation or intensive chemotherapy (Rossi et al., 2022; Wood et al., 2023). Clinical manifestations include ventilator-associated pneumonia, bloodstream infections, surgical site infections, and urinary tract infections, with mortality rates exceeding 50% in severe cases (Schwartz et al., 2024).

The emergence of multidrug-resistant (MDR) and carbapenem-resistant *P. aeruginosa* (CRPA) has prompted the World Health Organization to classify CRPA as a critical-priority pathogen for antimicrobial research and development (Tacconelli et al., 2018; Flores-Vega et al., 2025). Resistance arises from reduced outer membrane permeability, efflux pump upregulation, target site modification, and production of carbapenemases, particularly metallo-β-lactamases (MBLs) such as *bla_VIM_* and *bla_NDM_*, often encoded on mobile genetic elements (Karakonstantis et al., 2020; Brkic and Cirkovic, 2024).

Middle Eastern studies report a rising prevalence of these MBL genes, including *bla_NDM_* in 21% of CRPA isolates in Iraq (Alsaadi et al., 2020) and *bla_VIM_* in 19% of Iranian isolates (Vaez et al., 2018). In addition to resistance, *P. aeruginosa* expresses multiple virulence factors (e.g., *toxA*, *lasB*, *exoS*) linked to resistance mechanisms through shared regulatory networks (Moradali et al., 2017; Huang et al., 2025). Its large, adaptable genome (6.3–6.6 Mb) further complicates treatment (Grace et al., 2022; Hu et al., 2024).

Although strategies such as reverse vaccinology and immunoinformatics-based epitope prediction offer future promise, their clinical utility remains limited by genetic variability (Fereshteh et al., 2023; Zhu et al., 2025). Until then, the burden of CRPA continues to rise, particularly in vulnerable populations such as those with burns or cancer (Hasan et al., 2024a,b).

This study aimed to characterise the clinical and epidemiological features, resistance patterns, and molecular profiles of MDR *P. aeruginosa* isolates from burn, cancer, and other immunocompromised patients in Erbil, Iraq. Specifically, we assessed the prevalence and co-occurrence of *bla_VIM_* and *bla_NDM_* genes and their associations with phenotypic resistance patterns and AES-based carbapenemase classification to inform local antimicrobial stewardship and infection control efforts.

## 2 Materials and methods

### 2.1 Study design and population

A cross-sectional study was conducted between October 2024 and June 2025 across major public and private hospitals in Erbil, Iraq, including Nanakaly Hospital for Hematology and Oncology, Rizgary Teaching Hospital, the Burns and Plastic Surgery Hospital, Erbil Central Laboratory, Mihrabani Surgical Hospital, and additional private facilities. Hospitalized immunocompromised patients with culture-confirmed *P. aeruginosa* infections**—**attributed to malignancy, chemotherapy, burns, or chronic immunosuppressive conditions**—**were enrolled. Patients without confirmed infection or non-immunocompromised individuals were excluded. A total of 50 eligible patients were included and stratified into three clinical subgroups: cancer (*n* = 14), burn (*n* = 16), and other immunocompromised conditions (*n* = 20). Ethical approval was granted prior to data collection (see Section 2.7).

### 2.2 Specimen collection

**Figure 1:**
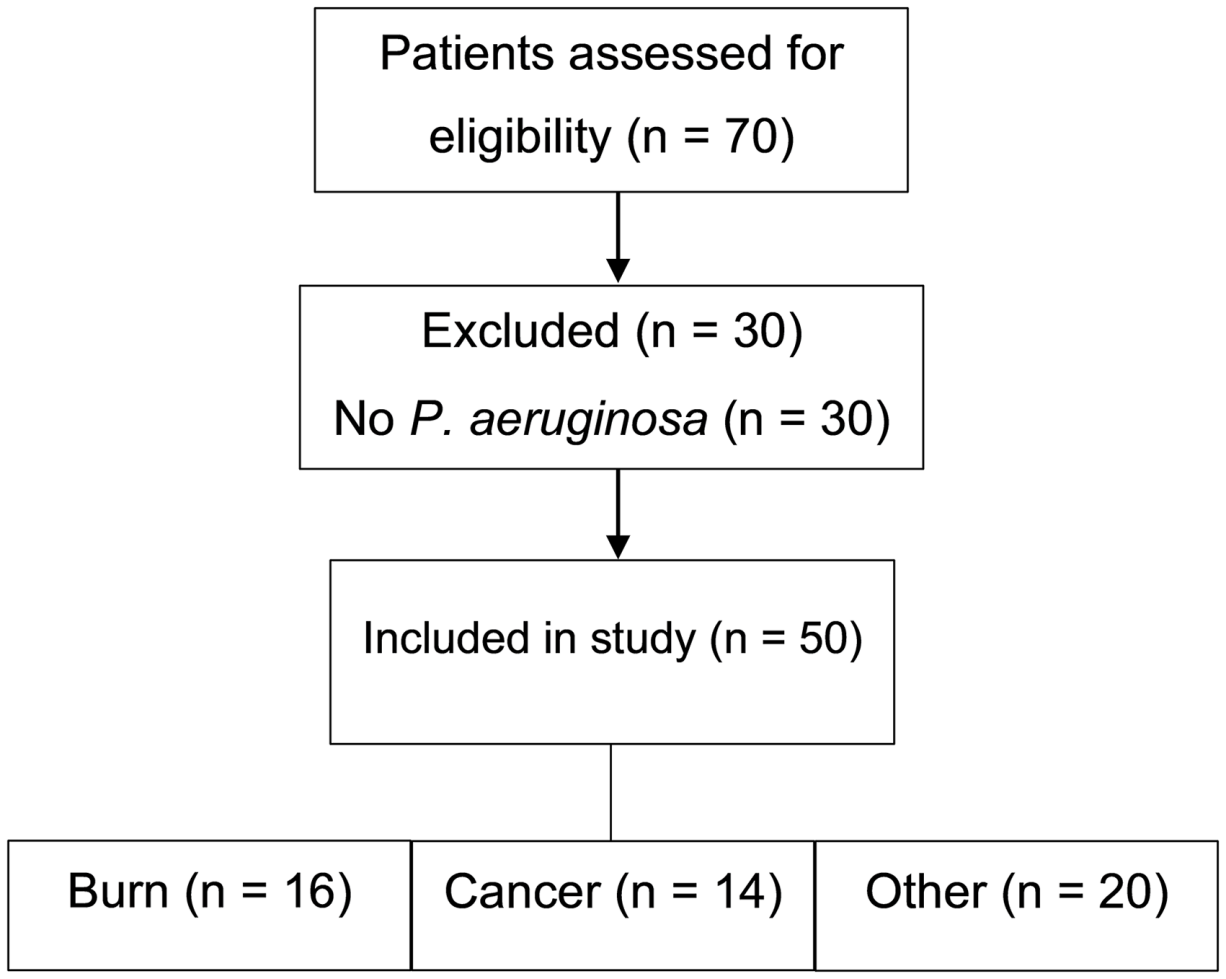
Flowchart of participant selection and inclusion in the study.

Seventy patients were initially screened for eligibility. Of these, 30 were excluded due to the absence of *P. aeruginosa* infection (Figure 1). Clinical specimens—including blood, urine, wound swabs, burn swabs, sputum, and bronchoalveolar lavage fluid—were collected using standard aseptic techniques and promptly transported to the microbiology laboratory. *P. aeruginosa* isolates were confirmed and preserved in brain heart infusion broth supplemented with 20% glycerol at −80 °C for molecular analysis. Only non-duplicate clinical isolates with confirmed identity and purity were included.

### 2.3 Microbiological identification and molecular confirmation

Bacterial isolation was performed using standard microbiological techniques. Clinical specimens were cultured on nutrient agar, MacConkey agar, and cetrimide agar and incubated at 37 °C for 24–48 h. Preliminary identification for *P. aeruginosa* was performed based on colony morphology, pigment production, oxidase test, catalase test, motility, and Gram staining. Confirmatory identification of *P. aeruginosa* was performed using the Vitek® 2 GN ID card system (bioMérieux, Marcy-l’Étoile, France). Molecular confirmation was performed by amplifying the conserved *16S rDNA* gene via polymerase chain reaction (PCR) using previously validated primers (Jarjees et al., 2021). Quality control was ensured using the reference strain *P. aeruginosa* ATCC 27853.

### 2.4 Antimicrobial susceptibility testing (AST)

Isolates underwent susceptibility testing using the Vitek® 2 automated system with AST-N419, AST-N222, AST-XN20, or AST-417 cards (bioMérieux, Marcy-l’Étoile, France). Antibiotic classes tested included β-lactams, carbapenems, aminoglycosides, fluoroquinolones, and polymyxins. Where required, disk diffusion testing was performed in accordance with CLSI and EUCAST guidelines. Interpretations were based on minimum inhibitory concentrations (MICs) or inhibition zone diameters, and appropriate standard control strains (e.g., *E. coli* ATCC 25922, *P. aeruginosa* ATCC 27853) were included.

#### 2.4.1 Definition of key variables

Multidrug-resistant (MDR) *P. aeruginosa* was defined as resistance to at least one agent in three or more antimicrobial categories, according to international consensus definitions. Carbapenem resistance was defined as resistance to at least one carbapenem (imipenem or meropenem), based on established clinical breakpoints. Prior carbapenem exposure referred to documented administration of any carbapenem within the 90 days preceding the date of culture collection. Mortality was defined as death occurring during the same hospitalization in which the *P. aeruginosa* infection was confirmed.

### 2.5 Molecular detection of *bla_VIM_* and *bla_NDM_* genes

#### 2.5.1 DNA extraction

Bacterial DNA was extracted from isolates cultured in brain heart infusion broth using the Bacterial DNA Preparation Kit (Jena Bioscience, Germany). The concentration and purity of the extracted DNA were assessed using a NanoDrop™ spectrophotometer (Thermo Scientific, USA). DNA samples were stored at −20 °C until further use.

#### 2.5.2 PCR amplification

PCR amplification of *bla_VIM_* (390 bp), *bla_NDM_* (621 bp), and *16S rDNA* (956 bp) genes was performed in a final volume of 25 μL using a Techne thermal cycler (UK). Each reaction mixture contained 12.5 μL GoTaq® Green Master Mix (Promega, USA), 3 μL of genomic DNA template, 1.5 μL of each primer (10 μM; Macrogen, South Korea), and 6.5 μL of DNase/RNase-free water (Promega, USA). A positive control (*P. aeruginosa* ATCC 27853) and a negative control were included in all PCR runs. Primers were selected based on previously published sequences (Kazemian et al., 2019; Jarjees et al., 2021). Thermocycling parameters and primer sequences are detailed in Supplementary Table S1.

#### 2.5.3 Agarose gel electrophoresis

PCR amplicons were separated on 2% agarose gels (Norgen Biotek, Canada) prepared in 1 × TBE buffer (Promega, USA). Gels were stained with Safe DNA Stain (SolarBio, China) and visualized using a UV transilluminator (Syngene, UK). A 1 kb DNA ladder (FroggaBio, Canada) was included in each run to estimate band sizes. The presence of target amplicons was confirmed by comparing observed bands with the expected product sizes. A complete list of laboratory instruments and Chemicals used is available in Supplementary Table S2.

### 2.6 Statistical analysis

All statistical analyses were performed using GraphPad Prism version 10.4.2 (GraphPad Software, San Diego, CA, USA). Categorical variables were analyzed using Chi-square or Fisher’s exact test, depending on expected frequencies. Associations between resistance gene carriage (*bla_VIM_, bla_NDM_*) and clinical variables were assessed using Fisher’s exact test.

One-way and two-way ANOVA, followed by Tukey’s post-hoc test, were employed to compare antibiotic resistance rates across patient groups and antibiotic classes. Assumptions of normality and homogeneity of variance were assessed using residual plots and appropriate statistical tests. A two-sided *p*-value < 0.05 was considered statistically significant. Missing data were handled using pairwise deletion. To reduce inter-site variability, standardized protocols were implemented across all participating laboratories. Effect sizes were not calculated due to the exploratory nature of the study and the relatively small sample size.

### 2.7 Ethical approval statement

Ethical approval for this study was obtained from the Medical Ethics Committee of Erbil Polytechnic University, Kurdistan Region, Iraq (Approval No. 25/0066 HRE; April 28, 2025). Written informed consent was obtained from all participants or their legal guardians prior to enrollment. The study was conducted in accordance with the principles of the Declaration of Helsinki and applicable institutional guidelines.

## 3 Results

### 3.1 Patient demographics and study population

A total of 50 hospitalized immunocompromised patients with culture-confirmed *P. aeruginosa* infections were enrolled between October 2024 and June 2025. The cohort included 29 males (58.0%) and 21 females (42.0%), with the most common age range being 21–30 years (23.9%). Patients were stratified into three clinical groups: cancer (*n* = 14), burn (*n* = 16), and other immunocompromised conditions (*n* = 20). Figure 1 illustrates the patient screening and group allocation process. Detailed demographic and clinical characteristics are summarized in Table 1 and visualized in Supplementary Figures S1–S3.

**Table 1:** Patient demographics, clinical specimens, and MDR status by group.

Parameters	Sub-parameters	Total (*n* = 50)	Cancer (*n* = 14)	Burn (*n* = 16)	Other (*n* = 20)
Age group (years)	Below 1	1 (2.2%)	1 (7.1%)	0 (0.0%)	0 (0.0%)
	1–10	4 (8.7%)	3 (21.4%)	0 (0.0%)	1 (5.0%)
	11–20	6 (13.0%)	3 (21.4%)	2 (12.5%)	1 (5.0%)
	21–30	11 (23.9%)	3 (21.4%)	6 (37.5%)	2 (10.0%)
	31–40	5 (10.9%)	0 (0.0%)	5 (31.3%)	0 (0.0%)
	41–50	8 (17.4%)	1 (7.1%)	2 (12.5%)	5 (25.0%)
	51–60	4 (8.7%)	1 (7.1%)	1 (6.3%)	2 (10.0%)
	61 and above	7 (15.2%)	2 (14.3%)	0 (0.0%)	5 (25.00%)
Gender	Male	29 (58.0%)	7 (50.0%)	10 (62.5%)	12 (60.0%)
	Female	21 (42.0%)	7 (50.0%)	6 (37.5%)	8 (40.0%)
Province	Erbil	42 (84.0%)	9 (64.3%)	14 (87.5%)	19 (95.0%)
	Kirkuk	2 (4.0%)	1 (7.1%)	1 (6.3%)	0 (0.0%)
	Duhok	1 (2.0%)	1 (7.1%)	0 (0.0%)	0 (0.0%)
	Sulaymaniyah	1 (2.0%)	0 (0.0%)	0 (0.0%)	1 (5.0%)
	Samarra	1 (2.0%)	1 (7.1%)	0 (0.0%)	0 (0.0%)
	Nineveh	1 (2.0%)	0 (0.0%)	1 (6.3%)	0 (0.0%)
	Baghdad	1 (2.0%)	1 (7.1%)	0 (0.0%)	0 (0.0%)
	Syria	1 (2.0%)	1 (7.1%)	0 (0.0%)	0 (0.0%)
Specimen	Wound swab	25 (50.0%)	3 (21.4%)	16 (100.0%)	6 (30.0%)
	Blood	13 (26.0%)	7 (50.0%)	0 (0.0%)	6 (30.0%)
	Urine	8 (16.0%)	3 (21.4%)	0 (0.0%)	5 (25.0%)
	Sputum	3 (6.0%)	1 (7.1%)	0 (0.0%)	2 (10.0%)
	Bronchoalveolar lavage (BAL)	1 (2.0%)	0 (0.0%)	0 (0.0%)	1 (5.0%)
Source of Infection	Burn wound infection	16 (32.0%)	0 (0.0%)	16 (100.0%)	0 (0.0%)
	Bloodstream infection	13 (26.0%)	7 (50.0%)	0 (0.0%)	6 (30.0%)
	Urinary tract infection	8 (16.0%)	3 (21.4%)	0 (0.0%)	5 (25.0%)
	Wound infection	7 (14.0%)	1 (7.1%)	0 (0.0%)	6 (30.0%)
	Respiratory infection	3 (6.0%)	1 (7.1%)	0 (0.0%)	2 (10.0%)
	Surgical site wound infection	2 (4.0%)	2 (14.3%)	0 (0.0%)	0 (0.0%)
	Lower respiratory tract infection / cystic fibrosis (CF)	1 (2.0%)	0 (0.0%)	0 (0.0%)	1 (5.0%)
MDR Status	MDR	33 (66.0%)	7 (50.0%)	15 (93.8%)	11 (55.0%)
	Non-MDR	17 (34.0%)	7 (50.0%)	1 (6.3%)	9 (45.0%)
Carbapenem Sensitivity	Carbapenem-resistant	29 (58.0%)	6 (42.9%)	15 (93.8%)	8 (40.0%)
	Carbapenem-sensitive	21 (42.0%)	8 (50.0%)	1 (0.0%)	12 (60.0%)

This table summarizes demographic data, specimen types, infection sources, and resistance status by group (cancer, burn, and other).

Complete clinical and exposure data were available for the burn and cancer groups. However, for the “other” immunocompromised group, data on prior carbapenem exposure and mortality outcomes were not recorded. These missing values (*n* = 20 each) were excluded from the relevant analyses, as noted in the corresponding tables and figures.

Among the 50 isolates, 29 (58.0%) were classified as carbapenem-resistant *P. aeruginosa* (CRPA) and were selected for molecular analysis. A total of 40 isolates—including CRPA, non-CRPA, and one control strain—were subjected to PCR detection of *bla_VIM_* and *bla_NDM_* genes.

### 3.2 Clinical specimens and infection sources

Wound swabs accounted for the majority of specimens (50.0%), followed by blood (26.0%) and urine (16.0%). The most frequent infection source was burn wound infections (32.0%), followed by bloodstream (26.0%) and urinary tract infections (16.0%) (Table 1; Supplementary Figures S4, S5). Representative wound infections, including percutaneous endoscopic gastrostomy (PEG) site involvement, are shown in Supplementary Figure S6. Supplementary Figures S7 and S8 display *P. aeruginosa* cultures grown on Cetrimide Agar under UV illumination and the corresponding colony morphology, respectively.

Detailed characteristics of burn injuries—including mechanism of injury, burn degree, total body surface area (TBSA) affected, anatomical distribution, season, place of occurrence, and reason for admission—are summarized in Supplementary Table S3.

### 3.3 Antimicrobial resistance profiles

**Figure 2:**
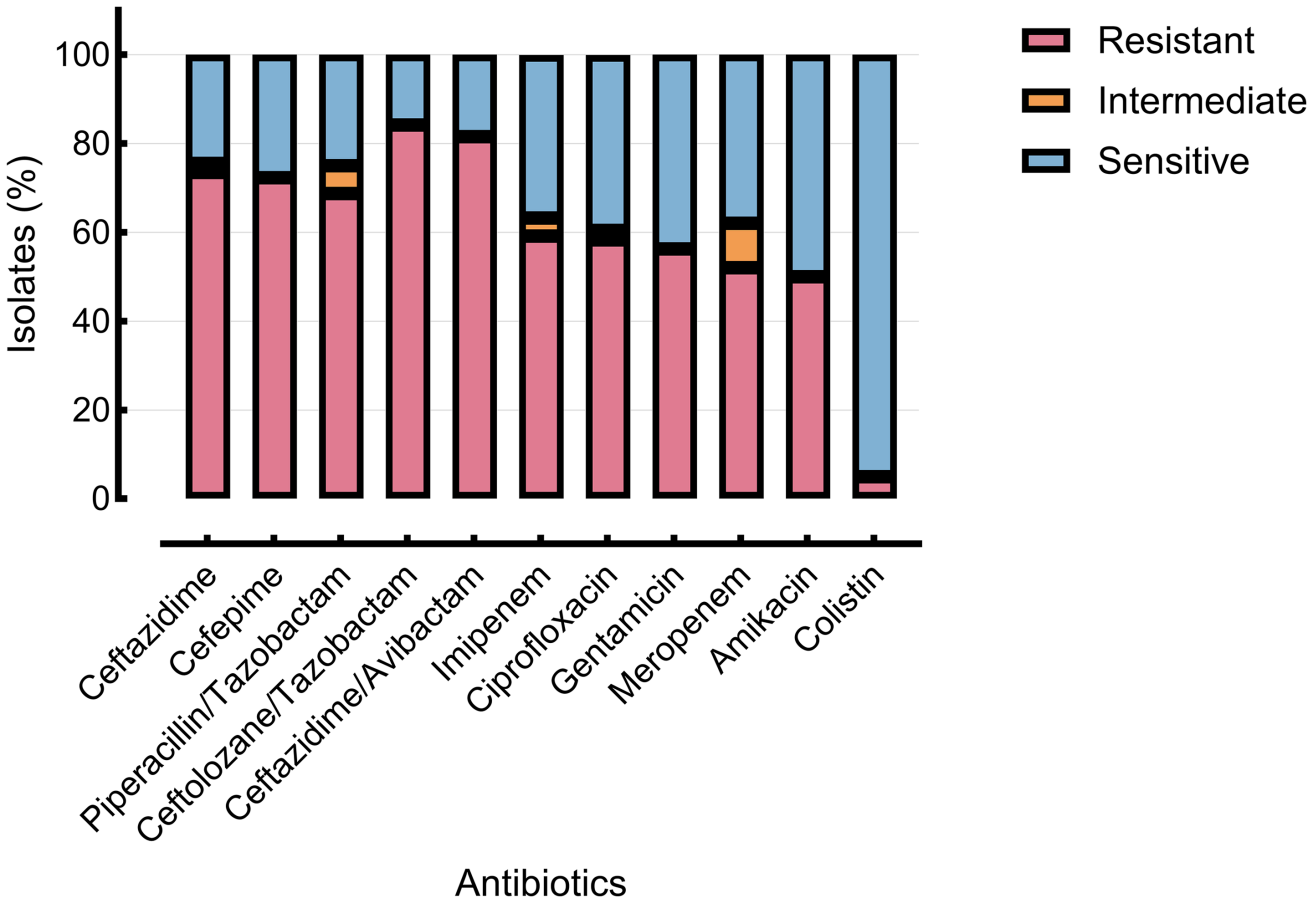
Antibiotic susceptibility patterns of *P. aeruginosa* isolates.

The highest resistance rates were observed for ceftolozane/tazobactam (84.2%), ceftazidime/avibactam (81.6%), and ceftazidime (73.5%). In contrast, colistin demonstrated the lowest resistance rate (5.0%). Among clinical subgroups, burn patients exhibited the highest resistance levels, followed by the cancer and other immunocompromised groups (Table 2; Figure 2). Comprehensive antimicrobial susceptibility data—including minimum inhibitory concentration (MIC) distributions and phenotypic resistance profiles—are provided in Supplementary Table S4.

**Table 2:** Resistance, intermediate, and susceptible profiles for all antibiotics.

Antibiotic	Resistant	(%)	Intermediate	(%)	Sensitive	(%)
Ceftazidime	36	73.5	1	2.0	12	24.5
Cefepime	34	72.3	0	0.0	13	27.7
Piperacillin/Tazobactam	33	68.8	3	6.3	12	25.0
Ceftolozane/Tazobactam	32	84.2	0	0.0	6	15.8
Ceftazidime/Avibactam	31	81.6	0	0.0	7	18.4
Imipenem	29	59.2	2	4.1	18	36.7
Ciprofloxacin	28	58.3	1	2.1	19	39.6
Gentamicin	27	56.3	0	0.0	21	43.8
Meropenem	26	52.0	5	10.0	19	38.0
Amikacin	24	50.0	0	0.0	24	50.0
Colistin	2	5.0	0	0.0	38	95.0

Percentages calculated based on isolates tested per antibiotic.

### 3.4 Multidrug resistance and carbapenem resistance

**Figure 3:**
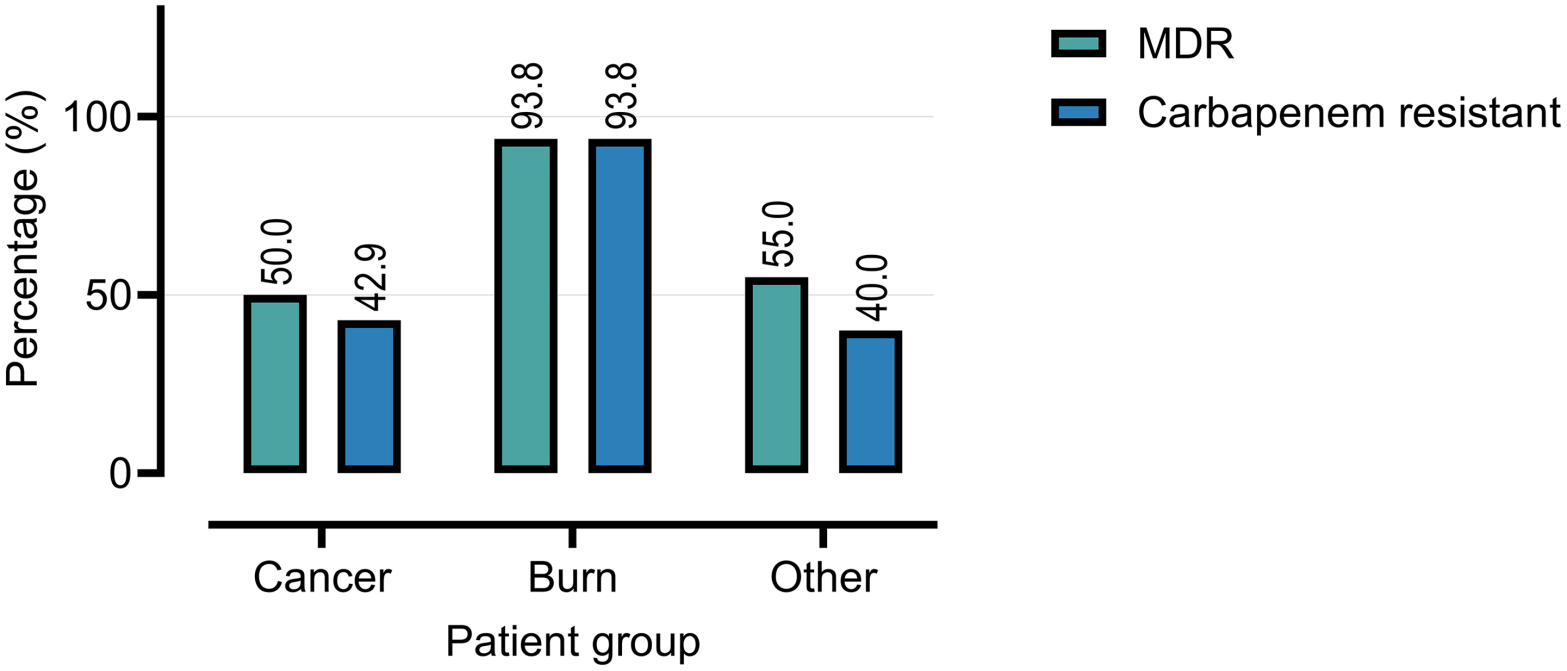
Prevalence of multidrug-resistant (MDR) and carbapenem-resistant *P. aeruginosa* isolates among clinical samples from cancer, burn, and other patient groups.

Overall, 66.0% of *P. aeruginosa* isolates were classified as multidrug-resistant (MDR). MDR prevalence was highest among burn patients (93.8%), compared to cancer patients (50.0%) and the other immunocompromised group (55.0%) (Table 1; Figure 3). Carbapenem resistance followed a similar trend: burn (93.8%), cancer (42.9%), and other (40.0%).

Prior carbapenem exposure was significantly associated with carbapenem resistance (76.2% vs. 57.1%, *p* = 0.0044), but showed no significant association with MDR status (*p* = 0.8011). Carbapenem-resistant isolates demonstrated significantly higher resistance across nearly all tested antibiotics compared to carbapenem-susceptible isolates (Table 3).

**Table 3:** Comparison of antibiotic resistance in carbapenem-resistant versus susceptible *Pseudomonas aeruginosa* (*n* = 50 each).

Antibiotic	Resistance	Carbapenem resistant (*n* = 50)		Carbapenem susceptible (*n* = 50)		*p* value
		*N*	%	N	%	
Ceftolozane/Tazobactam**	Resistant	28	96.6%	4	44.4%	0.0014
	Susceptible	1	3.5%	5	55.6%	
Ceftazidime/Avibactam**	Resistant	27	93.1%	4	44.4%	0.0042
	Susceptible	2	6.9%	5	55.6%	
Ceftazidime****	Resistant	29	100.0%	7	36.8%	<0.0001
	Susceptible	0	0.0%	12	63.2%	
Cefepime****	Resistant	29	100.0%	5	27.8%	<0.0001
	Susceptible	0	0.0%	13	72.2%	
Piperacillin/Tazobactam****	Resistant	27	96.4%	6	33.3%	<0.0001
	Susceptible	1	3.6%	12	66.7%	
Imipenem****	Resistant	29	100.0%	0	0.0%	<0.0001
	Susceptible	0	0.0%	18	100.0%	
Ciprofloxacin****	Resistant	27	96.4%	2	10.0%	<0.0001
	Susceptible	1	3.6%	18	90.0%	
Gentamicin****	Resistant	26	89.7%	2	10.5%	<0.0001
	Susceptible	3	10.3%	17	89.5%	
Meropenem****	Resistant	26	96.3%	0	0.0%	<0.0001
	Susceptible	1	3.7%	18	100.0%	
Amikacin****	Resistant	24	82.8%	0	0.0%	<0.0001
	Susceptible	5	17.2%	19	100.0%	
Colistin	Resistant	2	9.1%	0	0.0%	0.4923
	Susceptible	20	90.9%	18	100.0%	

Fisher’s exact test is used for statistical significance. **p < 0.01; ***p < 0.001; ****p < 0.0001.

**Figure 4:**
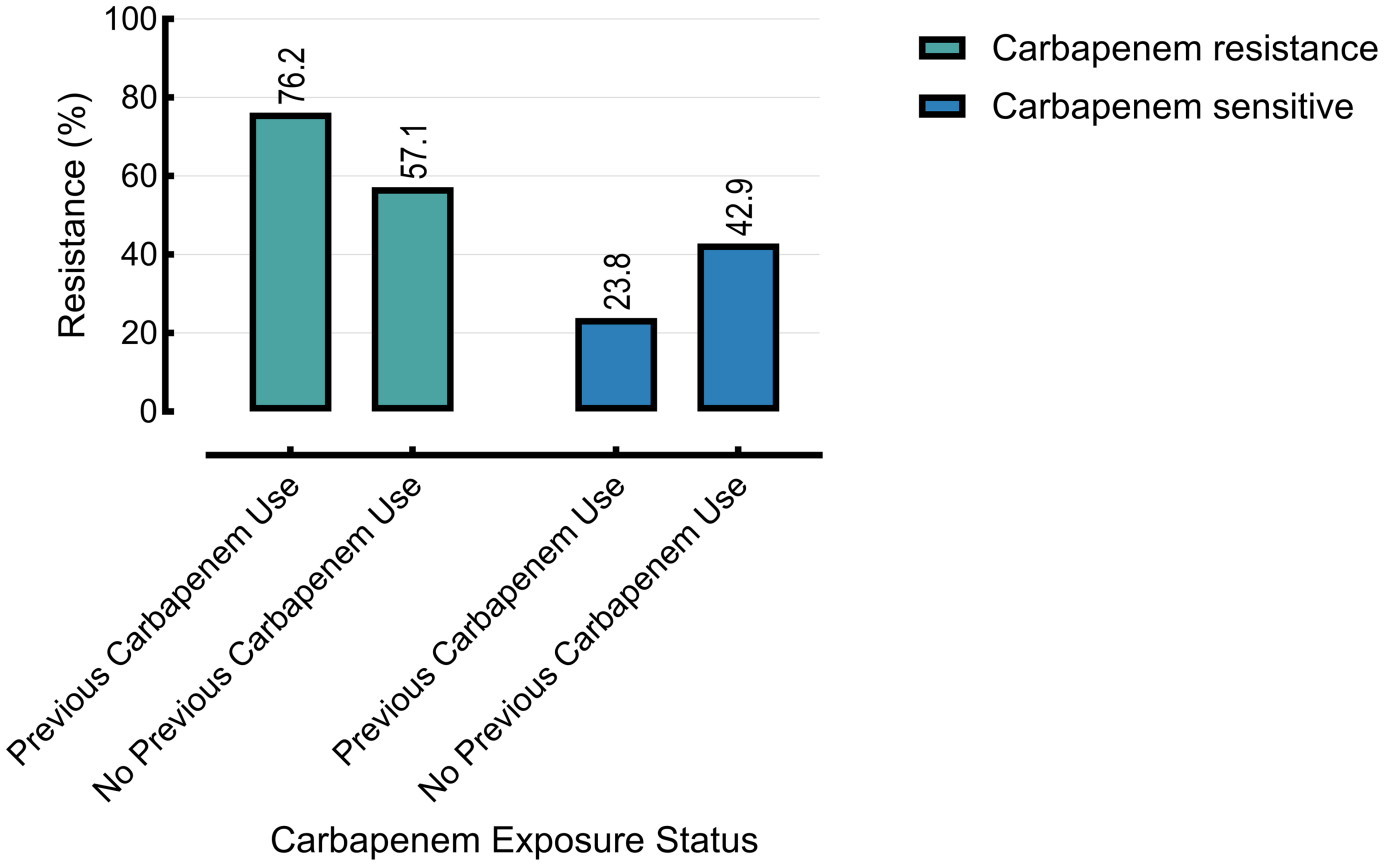
Carbapenem resistance is associated with prior use of carbapenems. Analysis includes burn and cancer groups only (*n* = 30); the other group lacked prior exposure data and was excluded.

**Figure 5:**
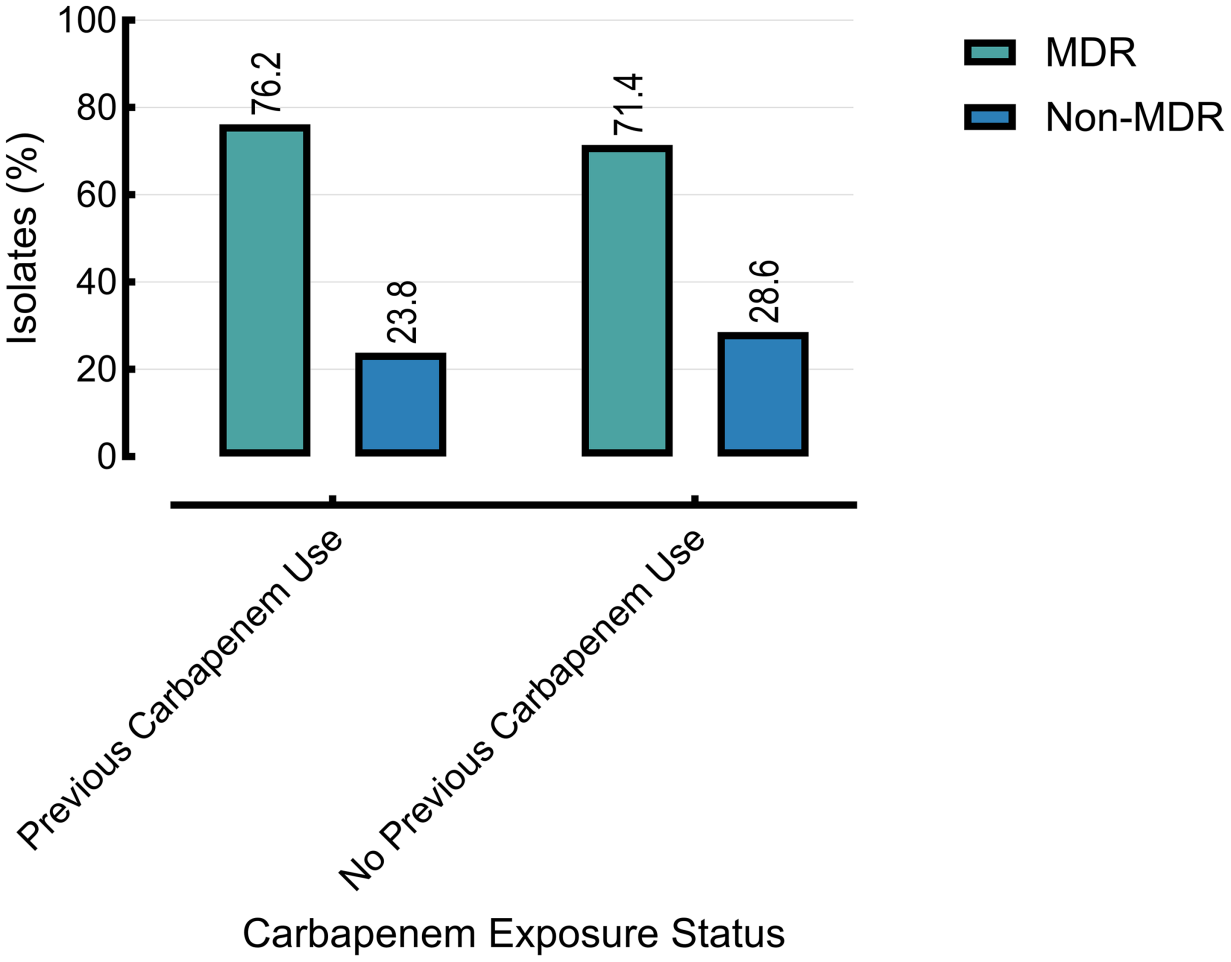
The multidrug-resistant (MDR) status is associated with prior carbapenem use. Other group excluded (*n* = 20) due to missing data on prior carbapenem exposure.

**Figure 6:**
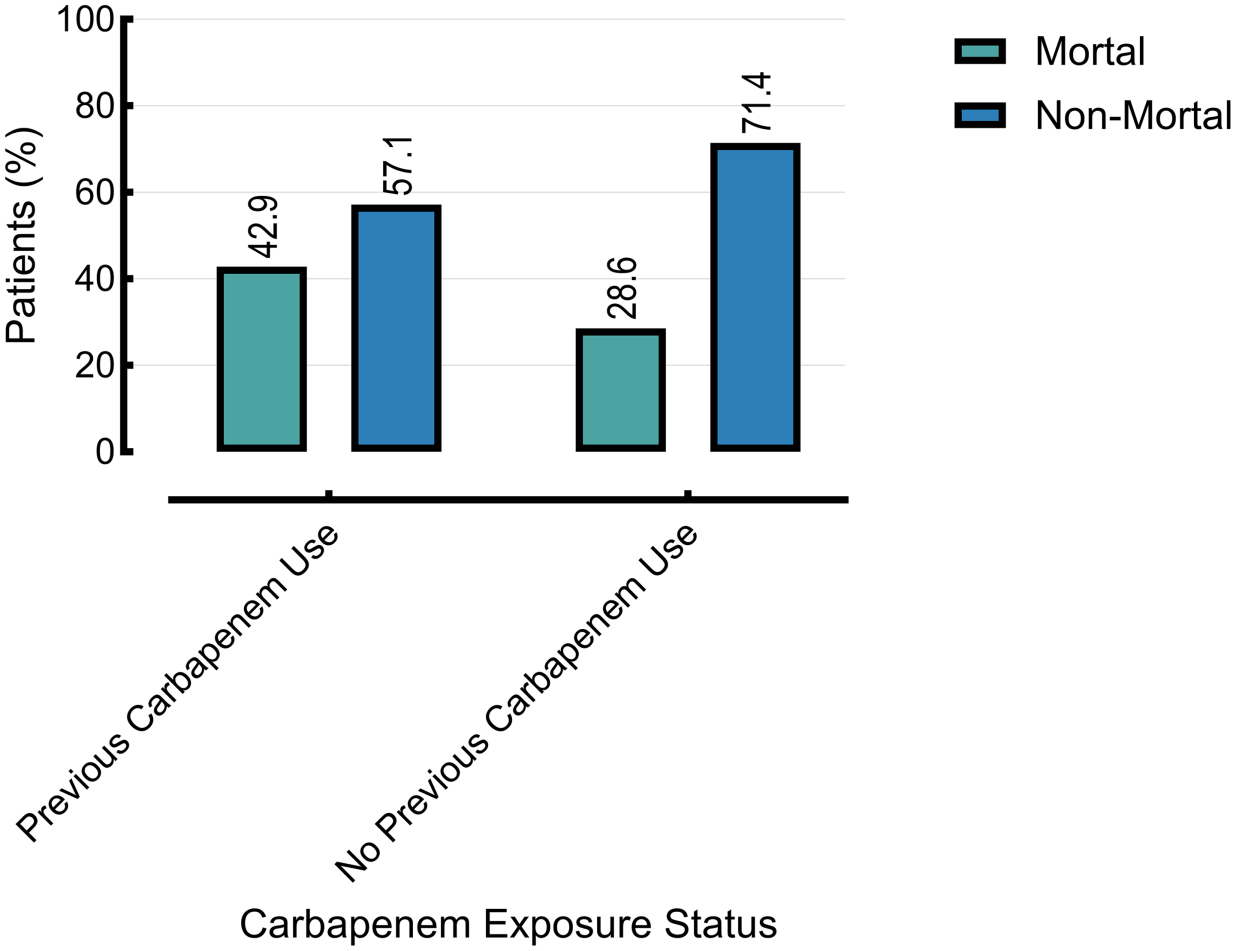
Mortality rate associated with prior carbapenem use. Analysis limited to burn and cancer groups (*n* = 30); mortality data were not available for the other group.

Mortality was also significantly higher in patients with prior carbapenem exposure (42.9%) compared to those without (28.6%, *p* = 0.0392) (Figures 4–6).

### 3.5 AES classification and resistance phenotypes

Carbapenemase production was identified in 40 of 50 *P. aeruginosa* isolates using the VITEK® 2 Advanced Expert System (AES). Among these, 26 isolates (65.0%) were classified as carbapenemase-producing subtypes. These isolates exhibited significantly higher rates of multidrug resistance (100%) and carbapenem resistance, including complete resistance to imipenem and 96.1% resistance to meropenem, compared to non-carbapenemase producers (MDR: 23.1%; carbapenem resistance: 15.4%; *p* < 0.0001).

**Figure 7:**
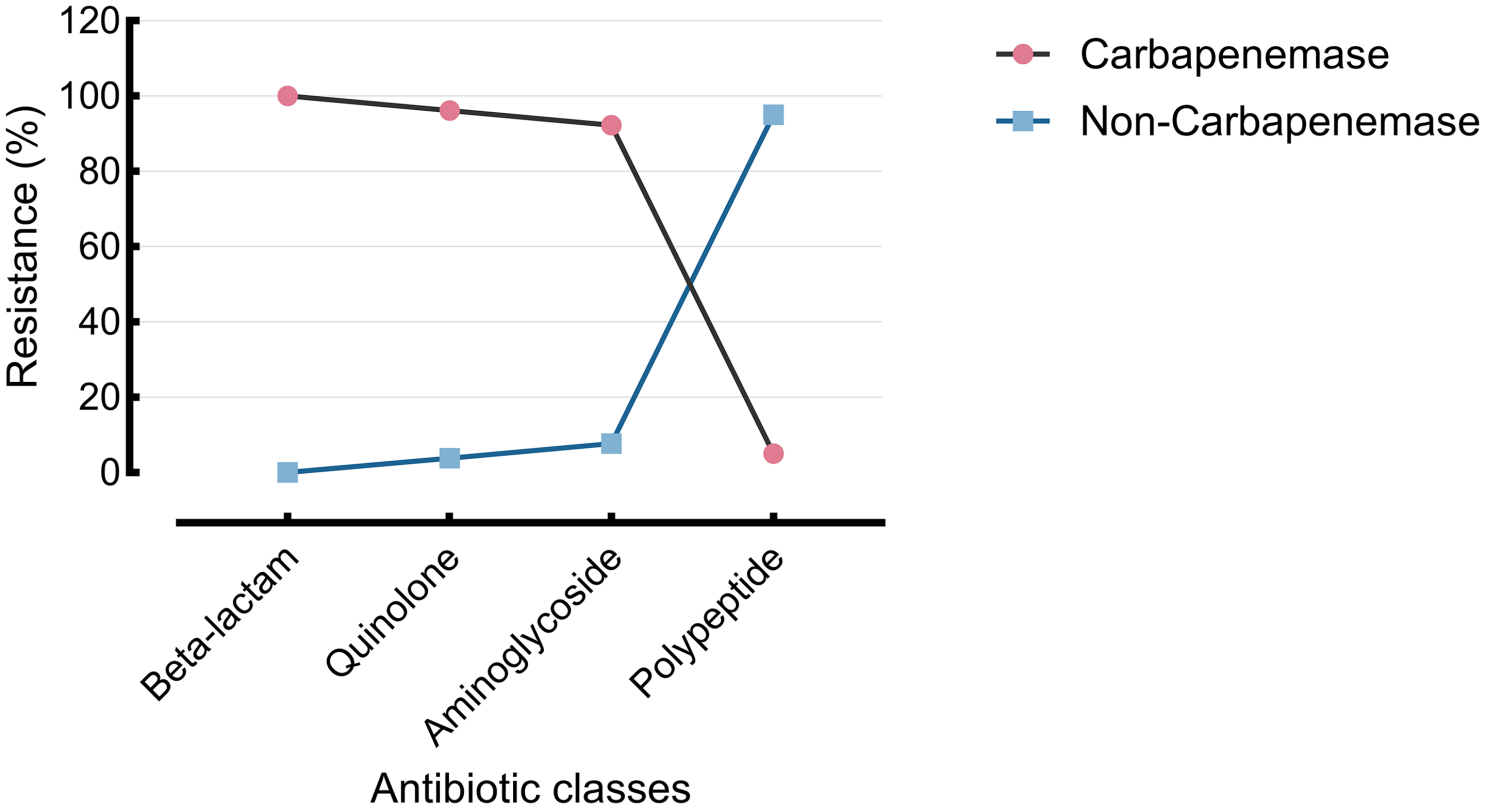
Resistance of the AES-predicted carbapenemase group across four antibiotic classes. Resistance rates across four major antibiotic classes are shown for AES-classified carbapenemase and non-carbapenemase *P. aeruginosa* isolates. Carbapenemase producers exhibited high resistance to β-lactams (100.0%), quinolones (96.2%), and aminoglycosides (92.3%), but low resistance to polypeptides (5.0%). Non-carbapenemase strains showed lower resistance across all classes, especially to polypeptides (5.0% vs. 77.6%).

Resistance profiles also varied significantly between AES subtypes. β-lactam resistance was higher among carbapenemase producers (*p* = 0.0014), as was quinolone resistance (*p* = 0.0016). Notably, colistin resistance was paradoxically higher among non-carbapenemase producers (77.6% vs. 5.0%; *p* < 0.0001) (Figure 7; Tables 4, 5).

**Table 4:** Association between AES subtypes and MDR/CRPA phenotypes in *Pseudomonas aeruginosa.*

AES Type	MDR (+)	MDR (−)	*p* value	Carbapenem-Resistant	Carbapenem-Susceptible	*p* value
AES with carbapenemase	26 (100.0%)	0 (0.0%)	<0.0001	26 (100.0%)	0 (0.0%)	<0.0001
AES without carbapenemase	3 (23.1%)	10 (76.9%)		2 (15.4%)	11 (84.6%)	

AES types are grouped into carbapenemase and non-carbapenemase categories. Statistical significance was determined using Fisher’s exact test.

**Table 5:** Class-specific resistance patterns in AES carbapenemase vs. non-carbapenemase *Pseudomonas aeruginosa.*

Antibiotic Class	AES Group	Resistant *n* (%)	Wild-type *n* (%)	Total	*p* value
β-lactams**	Carbapenemase	26 (100.0%)	0 (0.0%)	26	0.0014
	Non-Carbapenemase	13 (65.0%)	7 (35.0%)	20	
Aminoglycosides	Carbapenemase	24 (92.3%)	2 (7.7%)	26	>0.9999
	Non-Carbapenemase	15 (88.2%)	2 (11.8%)	17	
Quinolones**	Carbapenemase	25 (96.2%)	1 (3.9%)	26	0.0016
	Non-Carbapenemase	14 (58.3%)	10 (41.7%)	24	
Polypeptides****	Carbapenemase	1 (5.0%)	19 (95.0%)	20	<0.0001
	Non-Carbapenemase	38 (77.6%)	11 (22.5%)	49	

Statistical significance was determined using Fisher’s exact test. **p < 0.01; ***p < 0.001; ****p < 0.0001.

### 3.6 Resistance patterns by patient group and antibiotic class (two-way ANOVA analysis)

Two-way ANOVA revealed that both antibiotic type (*F*(10, 20) = 6.979, *p* = 0.0001; accounting for 37.7% of the total variance) and patient group (*F*(2, 20) = 47.76, *p* < 0.0001; accounting for 51.5% of the variance) had statistically significant effects on resistance rates. Spearman’s rank correlation (*R_s_* = −0.1825, *p* = 0.1547) indicated no evidence of heteroscedasticity; however, residuals failed to meet normality assumptions (*p* < 0.05 for all tests).

**Figure 8:**
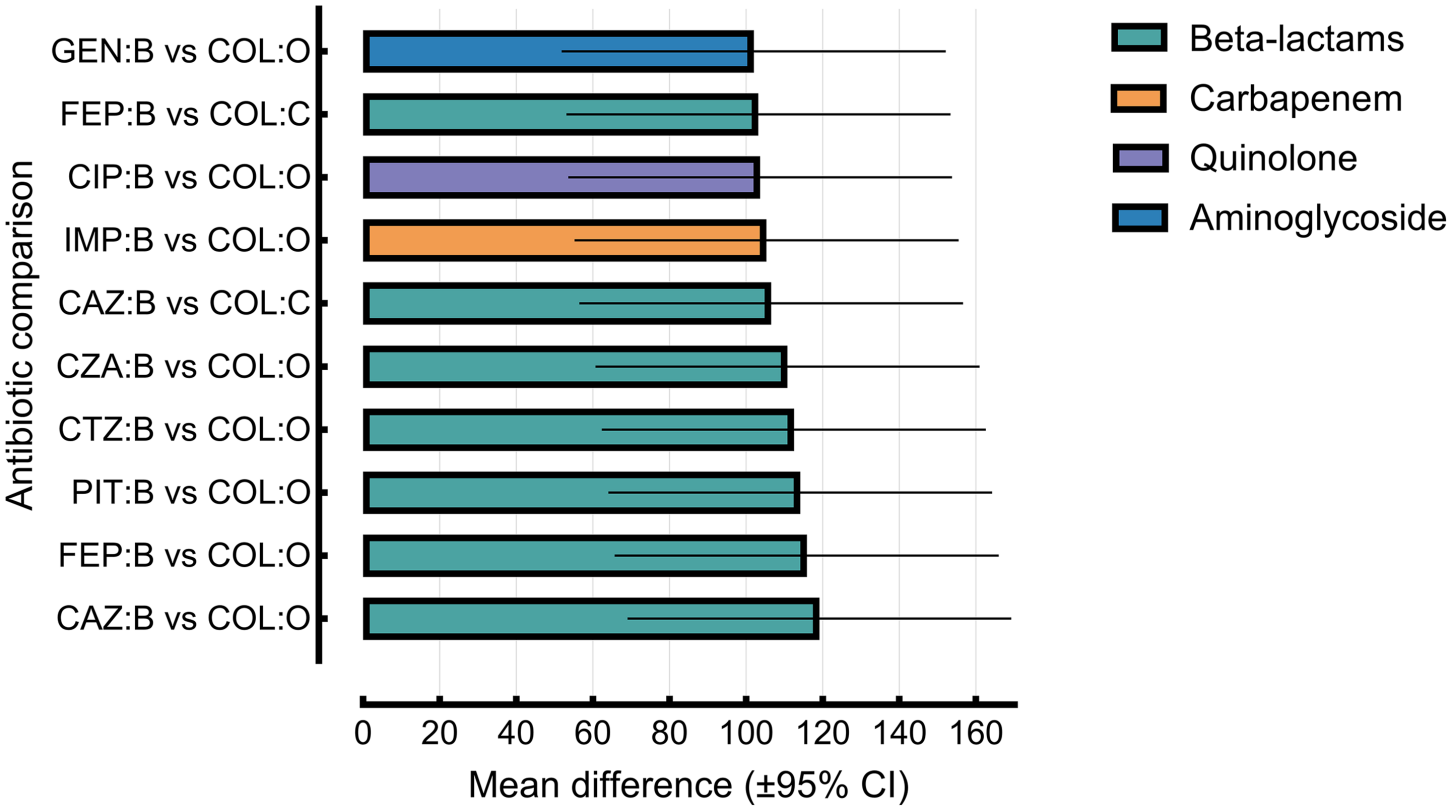
Pairwise resistance differences (burn vs. other groups) across selected antibiotics. Mean resistance differences (±95% CI) between burn patients and other clinical groups for selected antibiotics, grouped by class. β-lactams showed consistently higher resistance in burn isolates. Imipenem (carbapenem), ciprofloxacin (quinolone), and gentamicin (aminoglycoside) also demonstrated significantly increased resistance in burn patients compared to colistin sensitivity in other groups (adjusted *p* < 0.0001).

Tukey’s multiple comparison test revealed significantly higher resistance among isolates from burn patients compared to other groups. The most notable difference was observed between ceftazidime resistance in burn patients and colistin susceptibility in the other group (*Δ* = 119.1; 95% CI: 69.02–169.2; *p* < 0.0001). Supplementary Table S5 summarizes key pairwise comparisons; full results are available in Supplementary Table S6. An error-bar plot (mean difference ± 95% CI) visualizes selected comparisons such as CAZ: B vs. COL: O and FEP: B vs. COL: O (Figure 8). While some confidence intervals showed partial overlap, both main effects remained statistically robust (*p* < 0.0001).

To further explore trends within antibiotic classes, a stratified two-way ANOVA was performed. These sub-analyses demonstrated that the patient group was the primary source of variance across β-lactams, carbapenems, aminoglycosides, and fluoroquinolones, whereas differences between individual antibiotics within each class were generally not statistically significant. Detailed class-specific ANOVA results—including *F*-statistics, percentage variance explained, and residual diagnostics—are presented in Supplementary Figures S9–S14.

### 3.7 Prevalence and distribution of *bla_VIM_* and *bla_NDM_* genes

Among the 40 isolates tested by PCR, 19 (47.5%) were positive for *bla_VIM_*, 4 (10.0%) for *bla_NDM_*, and 12 (30.0%) co-harboured both genes. Five isolates (12.5%) were negative for both genes (Table 6). Gene distribution by patient group is summarised in Table 7. Burn patients showed the highest dual-carriage rate.

**Table 6:** Prevalence of *bla_VIM_* and *bla_NDM_* genes among *Pseudomonas aeruginosa* isolates (*n* = 40).

Gene Profile	No. of isolates	Percentage (%)
*bla_VIM_* only	19	47.5
*bla_NDM_* only	4	10.0
Both genes	12	30.0
Neither gene	5	12.5

**Table 7:** Distribution of *bla*_V*IM*_ and *bla_NDM_* genes by patient group.

Patient Group	*bla_VIM_*-positive	*bla_NDM_*-positive	Dual-positive (*bla_VIM_ + bla_NDM_*)	Neither-positive
Burn (*n* = 15)	6 (40.0%)	2 (13.3%)	6 (40.0%)	1 (6.7%)
Cancer (*n* = 14)	9 (64.3%)	0 (0.0%)	2 (14.3%)	3 (21.4%)
Other immunocompromised (*n* = 11)	4 (36.4%)	2 (18.2%)	4 (36.4%)	1 (9.1%)

PCR genotyping was limited to isolates meeting predefined DNA quality criteria.

### 3.8 Association of resistance genes with AES and carbapenem phenotypes

No significant association was found between *bla_VIM_* presence and AES β-lactam resistance phenotype (*p* = 0.601) or carbapenem resistance (*p* = 0.686). However, *bla_NDM_* carriage was significantly associated with both AES β-lactam resistance phenotype (*p* = 0.0169) and carbapenem resistance (*p* = 0.027) (Supplementary Tables S7 and S8).

### 3.9 Co-occurrence of *bla_VIM_* and *bla_NDM_*

Twelve isolates (30.0%) co-harboured both genes. Fisher’s exact test showed no significant association between *bla_VIM_* and *bla_NDM_* co-occurrence (*p* > 0.9999) (Supplementary Table S9).

### 3.10 PCR validation and gel electrophoresis

All isolates were confirmed as *P. aeruginosa* via *16S rDNA* PCR (956 bp). PCR yielded expected amplicons for *bla_VIM_* (390 bp) and *bla_NDM_* (621 bp). No non-specific bands were observed. Supplementary Figures S15–S20 show gel images for *16S rDNA*; Supplementary Figures S21–S24 for *bla_VIM_*; and (Supplementary Figures S25, S26) for *bla_NDM_*.

## 4 Discussion

*Pseudomonas aeruginosa* is a major cause of healthcare-associated infections (HAIs) worldwide and is recognized by the WHO as a critical-priority pathogen due to its intrinsic resistance mechanisms and remarkable capacity to acquire additional resistance determinants (World Health Organization, 2017; Centers for Disease Control and Prevention (CDC), 2019; Flores-Vega et al., 2025). Globally, the prevalence of MDR *P. aeruginosa* ranges from 15 to 30% in some regions, while emerging evidence from Asia and Africa indicates pooled rates of around 46% (95% CI: 37.1–55.0) (Horcajada et al., 2019; Reyes et al., 2023).

In the Middle East, MDR rates vary considerably—from 75.6% in Egypt to 0% in Morocco, with intermediate rates of 7.3% in Saudi Arabia and 8.1% in Qatar. In the Levant, MDR prevalence ranges from 64.5% in Lebanon to 12.4% in Iraq, with carbapenem resistance increasingly driven by metallo-β-lactamase (MBL) production (Al-Orphaly et al., 2021).

The present study reports an MDR rate of 66.0% and carbapenem resistance of 58.0% among clinical isolates in Erbil, Iraq. Burn patients exhibited the highest burden (93.8% MDR), surpassing international reports, where MDR *P. aeruginosa* prevalence in burn units ranges from 15.2% in India, 19–23.1% in Pakistan and Brazil, to 64–72% in Algeria, Tanzania, and U.S. tertiary-care burn units (Elsheikh and Makram, 2024). Substantial rates were also observed in cancer patients (MDR: 50.0%, CRPA: 42.9%), consistent with previous reports in oncology populations (Othman et al., 2014; Abdulhak et al., 2025). While these values are lower than those observed in our burn unit, they remain higher than MDR rates reported in oncology settings in some high-income countries, which typically range between 25 and 40% (Horcajada et al., 2019). Elevated β-lactam resistance in cancer patients likely reflects frequent healthcare interactions, extended courses of broad-spectrum antibiotic therapy, neutropenia, chemotherapy-induced mucositis, immunosuppression, or institutional differences in infection prevention and control practices (Gottesdiener and Satlin, 2023).

Our burn unit data align with international reports that identify burn units as high-risk environments for MDR pathogen emergence due to frequent invasive procedures, immunosuppression, and heavy empirical antibiotic use (Hmissi et al., 2023; Ullah et al., 2023). Compared to the 64.5% MDR rate reported in Lebanon and 52.5% in Jordan (Al-Orphaly et al., 2021), our burn patient MDR prevalence is notably higher, underscoring the urgent need for strengthened infection prevention and control (IPC) in such units.

At the molecular level, the co-carriage of *bla_VIM_* and *bla_NDM_* genes was observed in 30% of PCR-tested isolates, with the highest prevalence among burn patients. Although *bla_VIM_* alone did not show a statistically significant association with phenotypic carbapenem resistance (*p* = 0.686), *bla_NDM_* carriage was significantly correlated with carbapenem resistance (*p* = 0.027). These findings align with prior studies identifying *bla_NDM_* as a key driver of carbapenem resistance (Alsaadi et al., 2020; Seyedi et al., 2022). The lack of association for *bla_VIM_* may reflect variability in expression, compensatory resistance mechanisms, or regional strain differences.

The VITEK® 2 Advanced Expert System (AES) provided additional insights into phenotypic resistance profiles. All AES-classified carbapenemase-producing isolates were also MDR and exhibited near-universal resistance to both imipenem (100%) and meropenem (96.1%), supporting its diagnostic utility in resource-limited settings where molecular testing may not be readily available (Chan and Leroi, 2021; Hackel and Bailey-Person, 2025; Laço et al., 2025).

The two-way ANOVA and Tukey’s post-hoc test revealed significant differences in resistance levels across patient groups and antibiotics. Burn patients exhibited significantly higher resistance to β-lactams and carbapenems, particularly ceftazidime, cefepime, and imipenem (*p* < 0.0001). While prior carbapenem exposure was significantly associated with carbapenem resistance (*p* = 0.0044), it was not predictive of MDR status (*p* = 0.8011). This suggests that intrinsic chromosomal mechanisms—such as AmpC β-lactamase overexpression, OprD porin loss, and efflux pump upregulation—may play a more dominant role in the development of MDR than antimicrobial exposure alone (Karakonstantis et al., 2020).

Moreover, the mortality rate was significantly higher among patients with prior carbapenem exposure (42.9%) compared to those without (28.6%, *p* = 0.0392), highlighting the clinical impact of antimicrobial resistance. While promising strategies such as reverse vaccinology and epitope-based vaccine design are under investigation (Fereshteh et al., 2023; Zhu et al., 2025), their translation into clinical practice remains distant. In the interim, robust surveillance, targeted antimicrobial stewardship, and enhanced infection control measures are critical to reducing CRPA transmission and associated morbidity and mortality.

## 5 Conclusion

Our study demonstrates the substantial burden of multidrug-resistant and carbapenem-resistant *P. aeruginosa* in immunocompromised patients, particularly those in burn units. The high frequency of resistance—especially among AES-classified carbapenemase producers and *bla_NDM_*-positive strains—highlights the need for urgent and targeted intervention. In resource-limited settings, the AES system offers a valuable surrogate for molecular testing, supporting rapid treatment decisions and infection control efforts. Integration of advanced diagnostics with routine susceptibility testing and molecular surveillance is essential to mitigate the growing threat of CRPA in healthcare settings.

## Data Availability

The raw data supporting the conclusions of this article, including MIC distributions, resistance profiles, pairwise comparisons, full statistical results, and gene -resistance associations (Tables S4-S9, including the .xlsx dataset), have been deposited in Figshare and are publicly available at: https://doi.org/10.6084/m9.figshare.29919473. Additional requests for data can be directed to the corresponding author at bahra.hamad@epu.edu.iq.

## Supplementary material

The Supplementary material for this article can be found online at: https://www.frontiersin.org/articles/10.3389/fmicb.2025.1672531/full#supplementary-material. The complete dataset, including all supplementary figures and tables, has also been deposited in Figshare and is publicly available at: https://doi.org/10.6084/m9.figshare.29919473.

The Supplementary material includes 26 figures and 9 tables. Figures S1–S5 present demographic and clinical data; Figure S6 shows wound characteristics in cancer patients. Figures S7–S8 depict microbiological findings on cetrimide agar. Figures S9–S14 summarise statistical analyses of antibiotic resistance. Figures S15–S20 confirm *16S rDNA* amplification by PCR. Figures S21–S24 and S25–S26 show amplification of *bla_VIM_* and *bla_NDM_* genes, respectively, with appropriate controls.

Table S1 provides thermocycling parameters and primer sequences. Table S2 lists laboratory instruments and chemicals. Burn injury characteristics are summarised in Table S3. Table S4 presents MIC distributions and resistance profiles. Table S5 outlines key pairwise comparisons; full results are in Table S6 (.xlsx). Tables S7 and S8 explore associations between *bla_VIM_*, *bla_NDM_*, AES phenotypes, and carbapenem resistance. Table S9 reports non-significant *bla_VIM_*, *bla_NDM_* co-occurrence.
