# Supplementary material for: Molecular detection of blaVIM and blaNDM in multidrug-resistant Pseudomonas aeruginosa from cancer and burn patients in Erbil, Iraq
Source: Front Microbiol. 2025 Sep 15;16:1672531. doi: 10.3389/fmicb.2025.1672531 (PMC12477123; doi:10.3389/fmicb.2025.1672531)
Supplement: Supplementary file 1 [file Data_Sheet_1.zip › latest_supplementary_material file/Supplementary_Figures With Legends.pdf]

Supplementary Figures

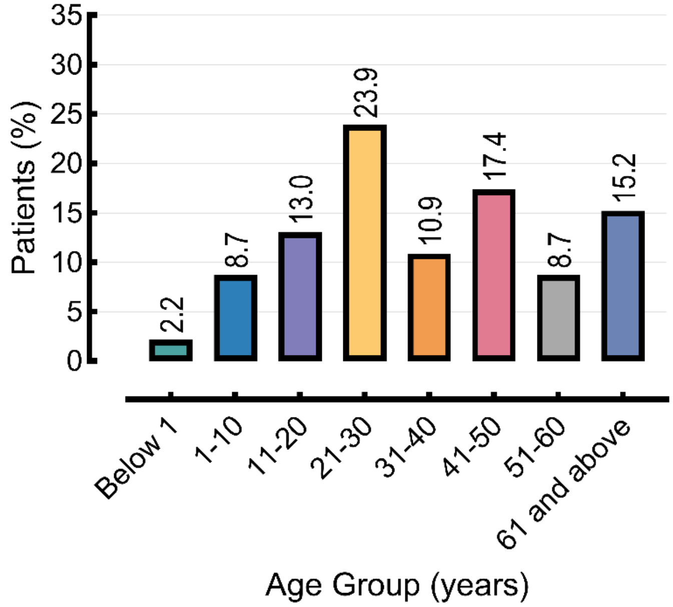

Supplementary Figure 1. Age distribution of patients.

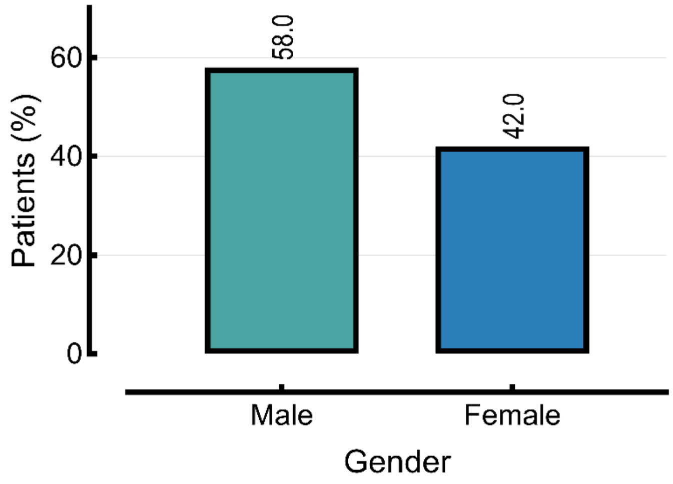

Supplementary Figure 2. Gender distribution of patients.

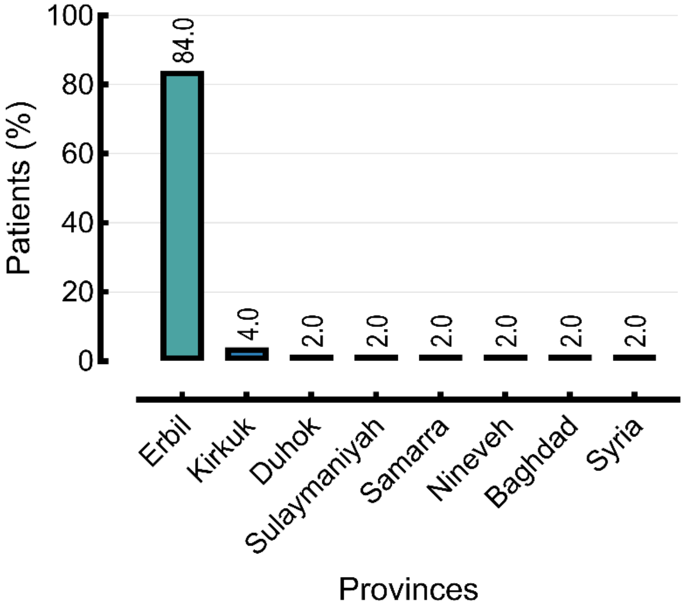

Supplementary Figure 3. Geographic distribution of patients by province.

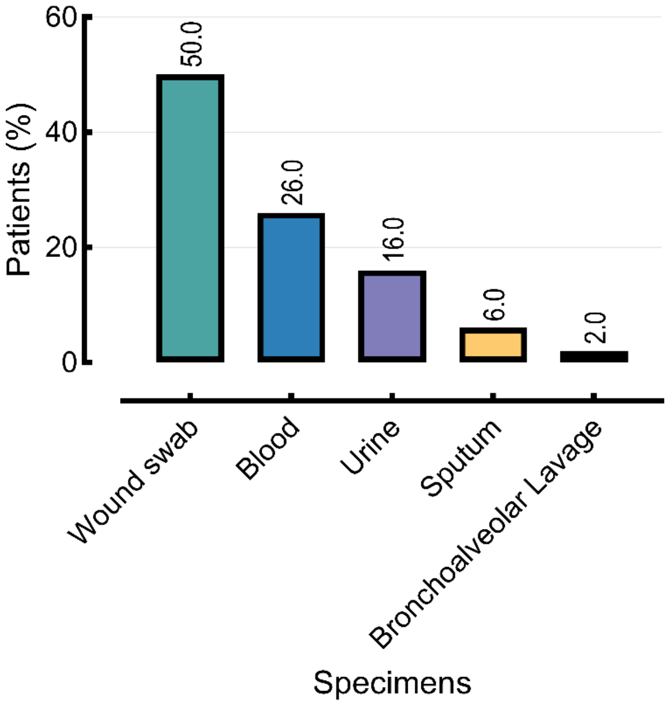

Supplementary Figure 4. Specimen type distribution.

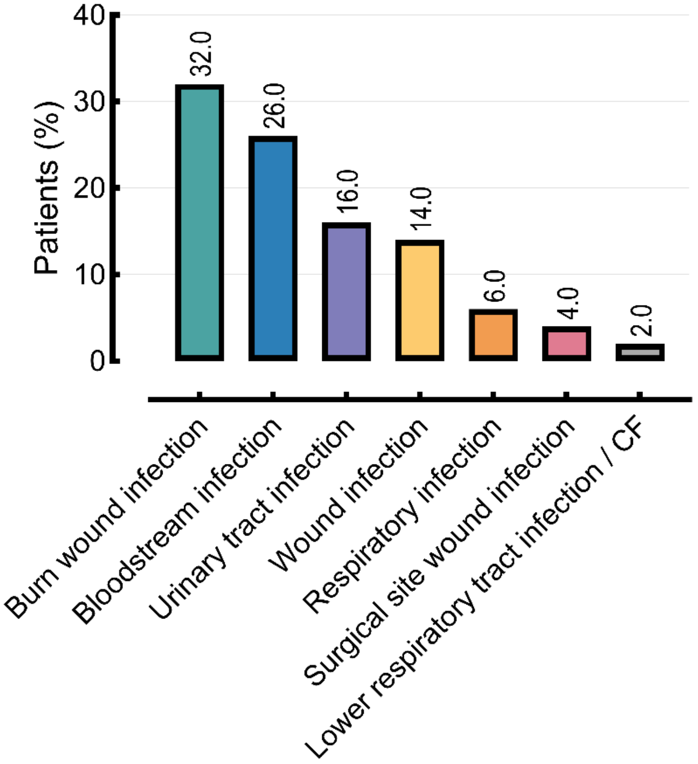

Supplementary Figure 5. Infection source distribution.

A **B**

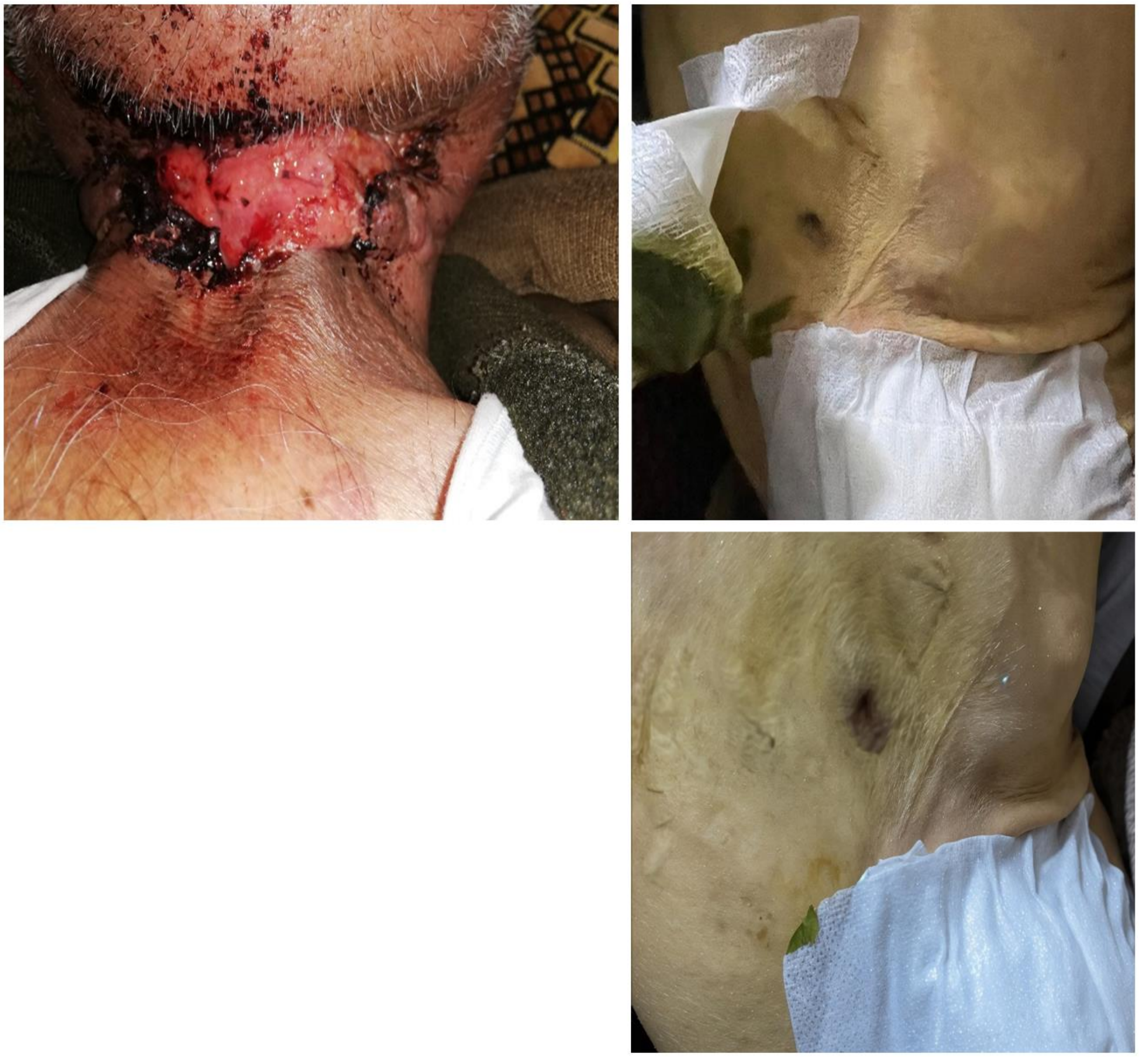

Supplementary Figure 6. Wound characteristics from cancer patients and isolation of *Pseudomonas aeruginosa*. *Image property of the author.*

(A) Moist wound from a cancer patient, showing visible signs of inflammation and exudate, where *Pseudomonas aeruginosa* was isolated.

(B) Wound site at percutaneous endoscopic gastrostomy (PEG) tube insertion in a stomach cancer patient, showing distinctive green drainage confirmed as *Pseudomonas aeruginosa*.

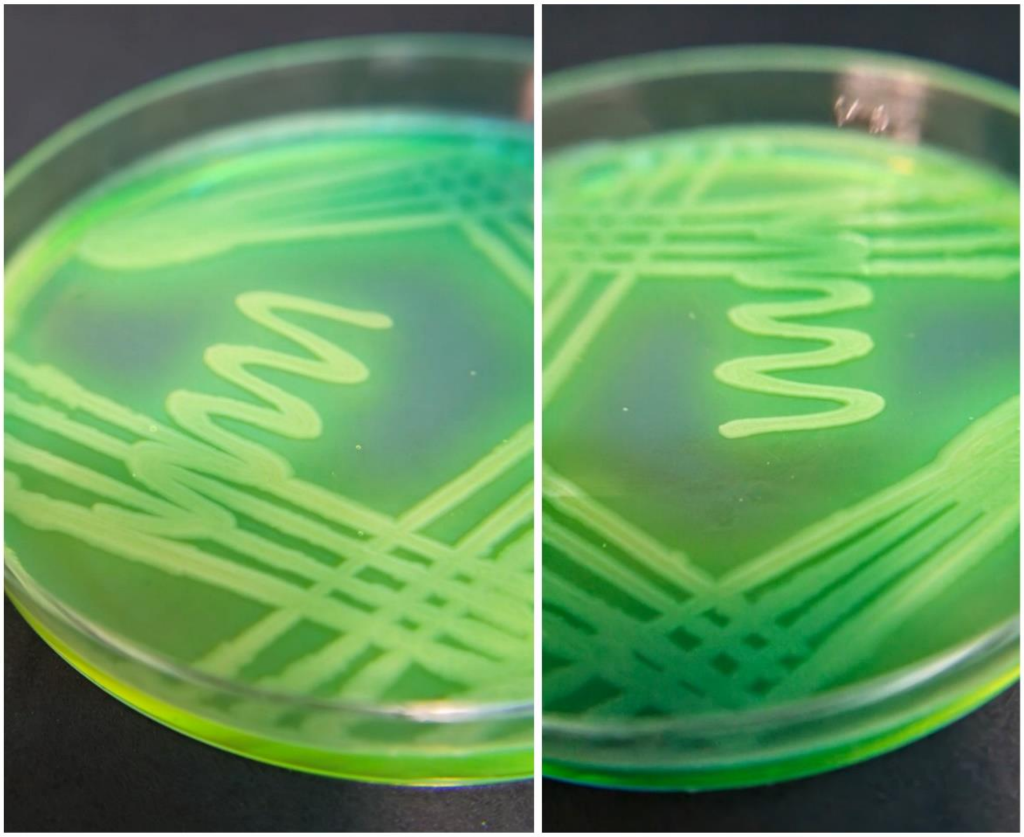

Supplementary Figure 7. Growth of *Pseudomonas aeruginosa* on cetrimide agar under UV light results in yellow–green pyoverdine and blue–green pyocyanin. *Image property of the author.*

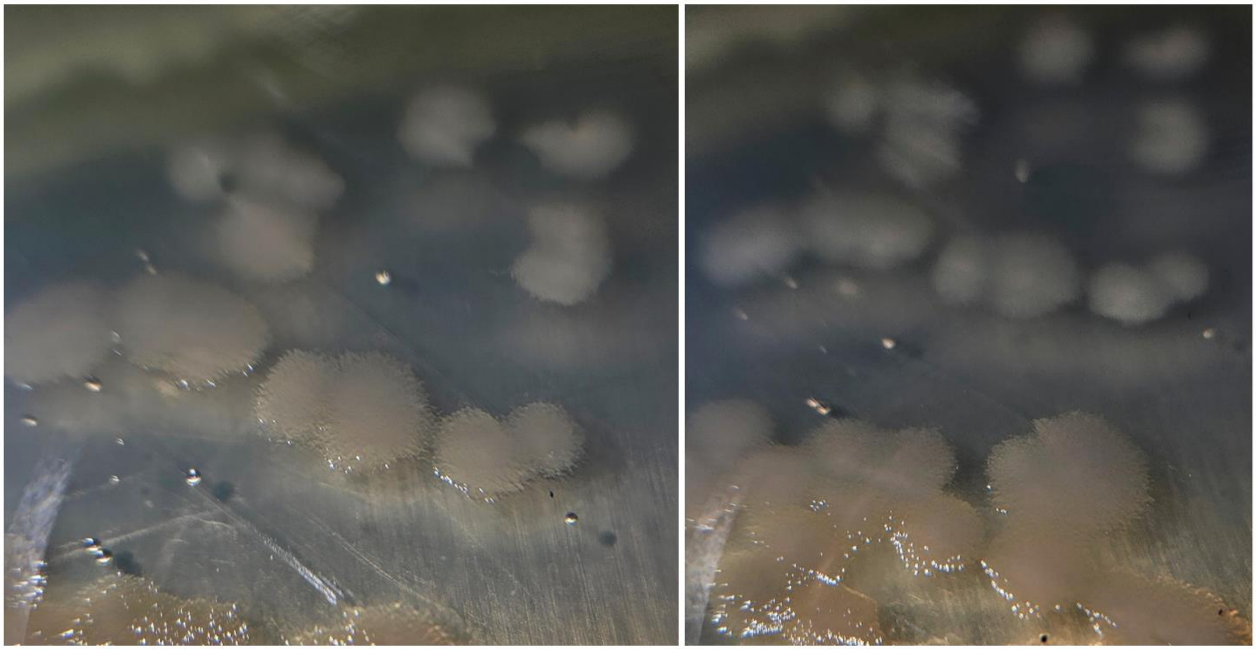

Supplementary Figure 8. *Pseudomonas aeruginosa* colonies on cetrimide agar have circular, irregular, and undulating morphologies. *Image property of the author.*

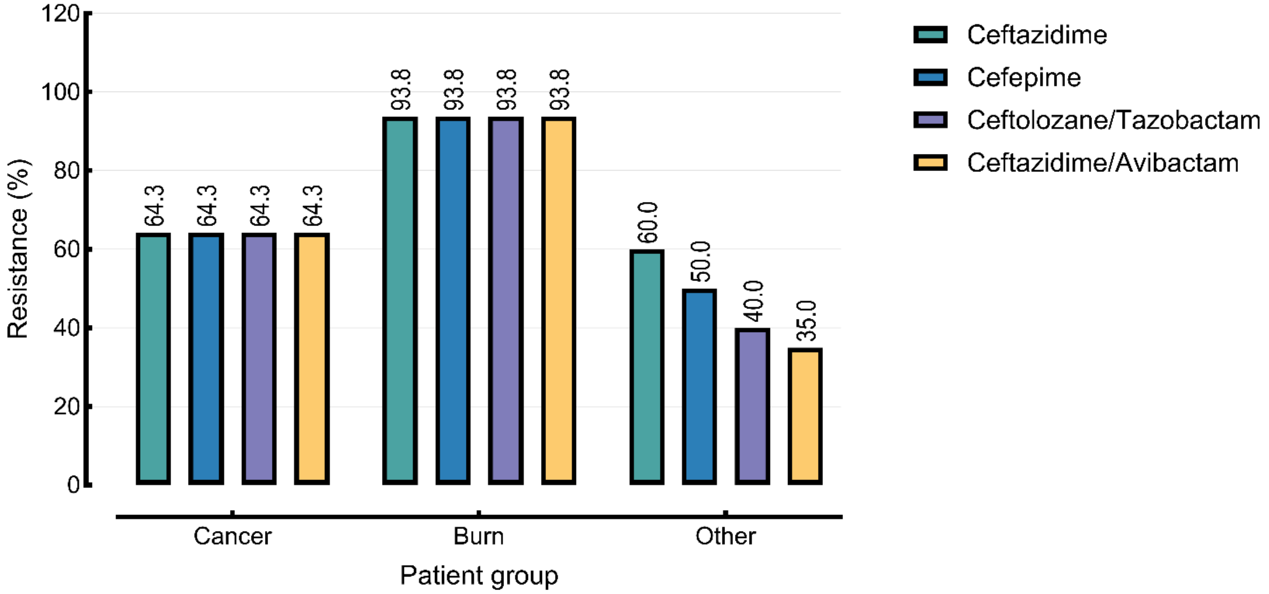

Supplementary Figure 9. Two-way ANOVA results for β-lactam antibiotic resistance among *Pseudomonas aeruginosa* isolates. The patient group accounted for 92.6% of the total variation, $F(2,6) = 56.13$, $p = 0.0001$, while the antibiotic type explained only 2.5% $F(3,6) = 1.000$, $p = 0.4547$. No

heteroscedasticity was detected ($Rs = -0.3732$, $p = 0.1154$), and the residuals satisfied the normality assumptions.

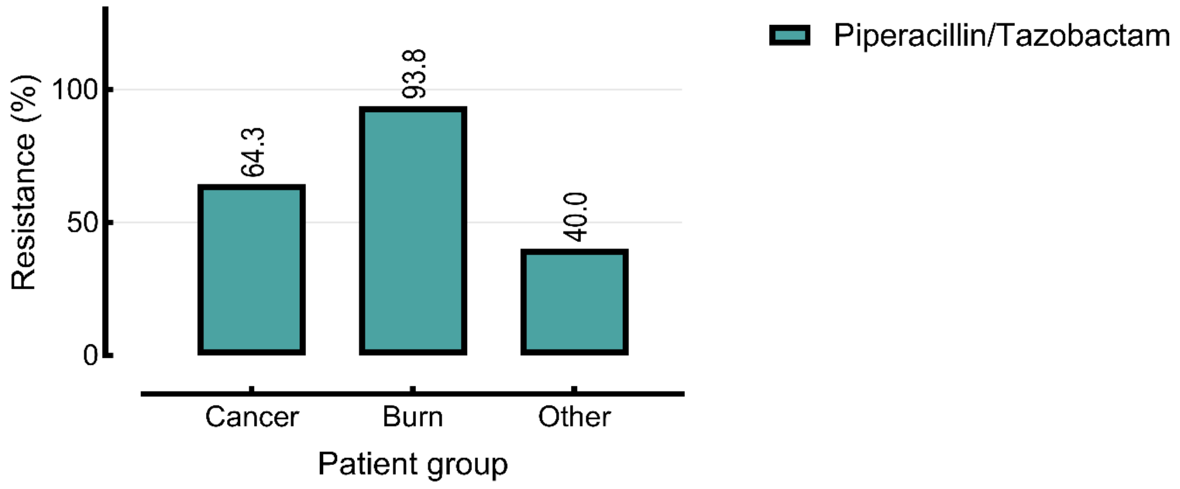

Supplementary Figure 10. Resistance to piperacillin/tazobactam among *Pseudomonas aeruginosa* isolates from different patient groups. A statistically significant variation was observed across patient groups ($p = 0.0027$), with burn patients exhibiting the highest resistance rates.

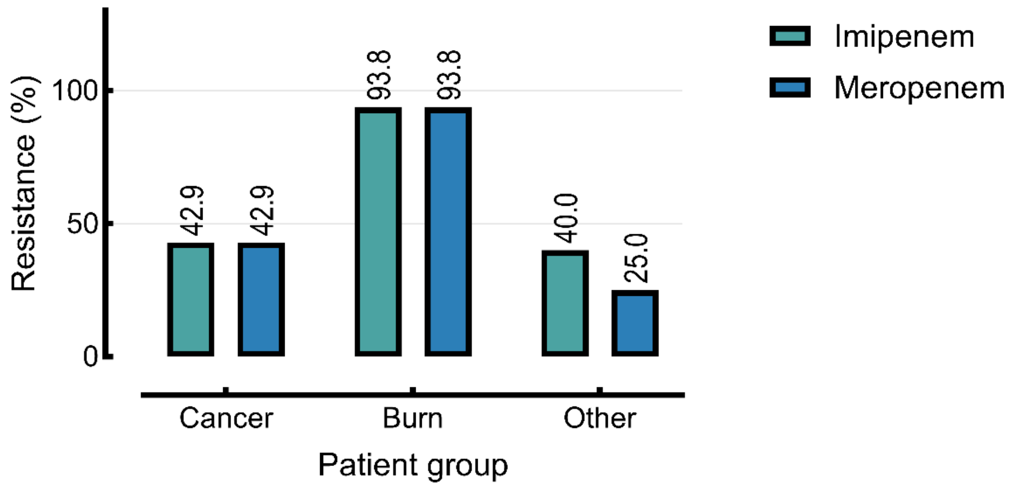

Supplementary Figure 11. Resistance to carbapenems (imipenem versus meropenem) among *Pseudomonas aeruginosa* isolates from different patient groups. The patient group explained a significant portion of the variance (97.5%; $F(2, 2) = 57.32$, $p = 0.0171$), while the antibiotic type had no significant effect ($F(1, 2) = 1.000$, $p = 0.4226$).

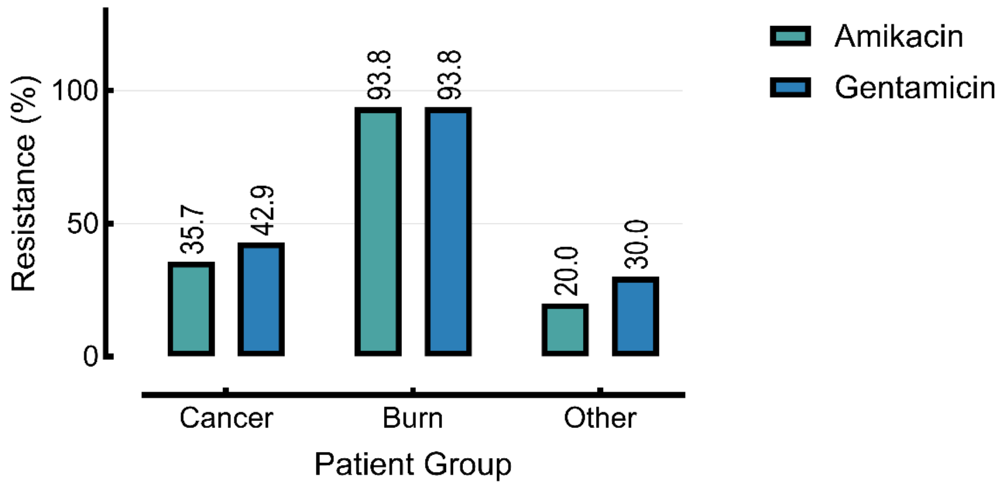

Supplementary Figure 12. Resistance to aminoglycosides (amikacin versus gentamicin) among *Pseudomonas aeruginosa* isolates from different patient groups. The patient group explained 98.6% of the variance ($F(2, 2) = 198.4$, $p = 0.0050$), while the drug type was not statistically significant ($F(1, 2) = 3.69$, $p = 0.1945$).

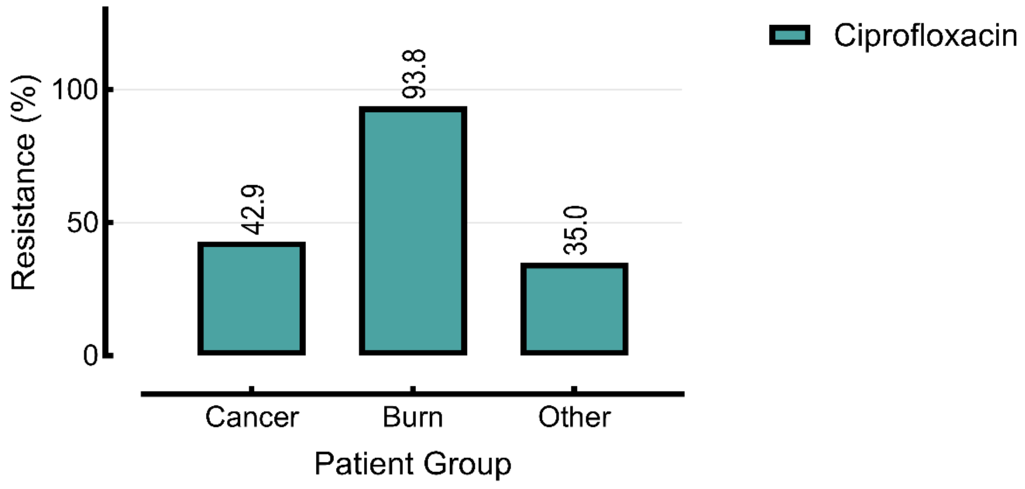

Supplementary Figure 13. Resistance to ciprofloxacin among *Pseudomonas aeruginosa* isolates from different patient groups. A significant difference was observed across patient groups ($p = 0.0007$).

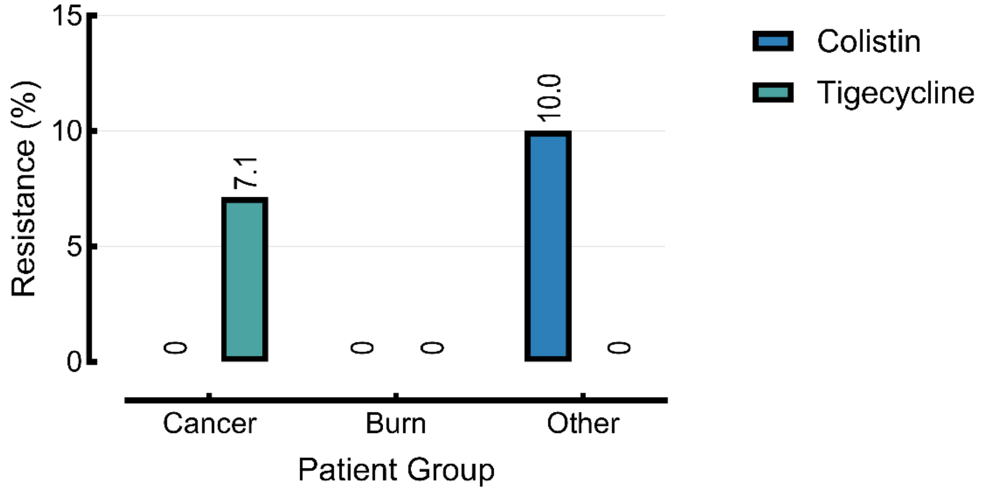

Supplementary Figure 14. Resistance to colistin and tigecycline among *Pseudomonas aeruginosa* isolates. Neither the patient group nor the antibiotic type significantly affected resistance ($p > 0.7$), with both drugs showing consistently low resistance levels.

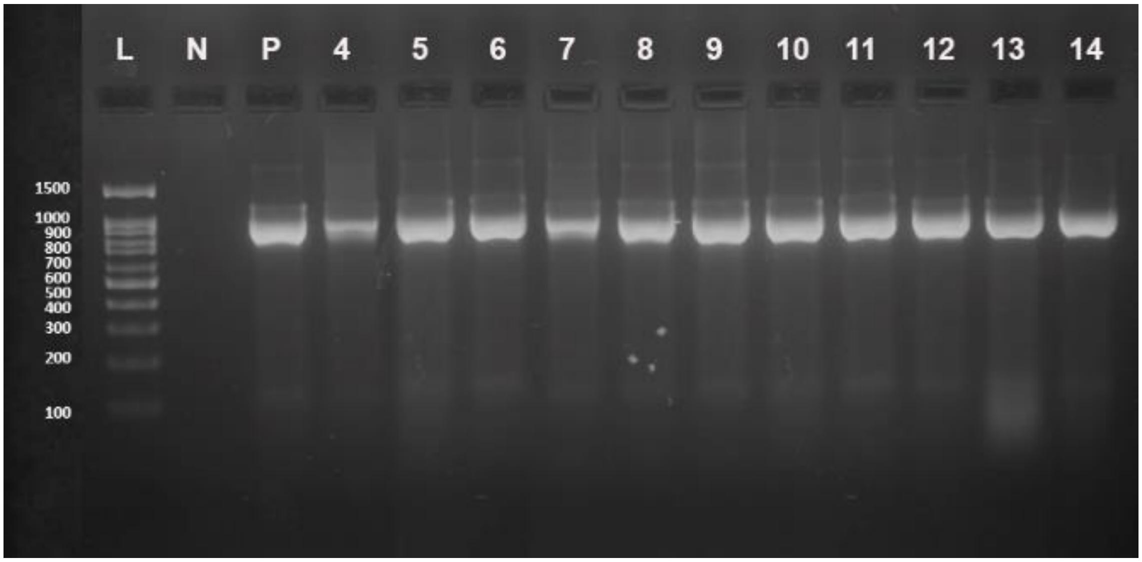

Supplementary Figure S15. PCR gel electrophoresis showing amplification of the *16S rDNA* gene (~956 bp) in *Pseudomonas aeruginosa* isolates. Lane L: 100 bp DNA ladder; Lane N: negative control; Lane P: positive control; Lanes 4–14: amplified products from clinical isolates. Clear bands at the expected size confirm successful amplification for molecular identification.

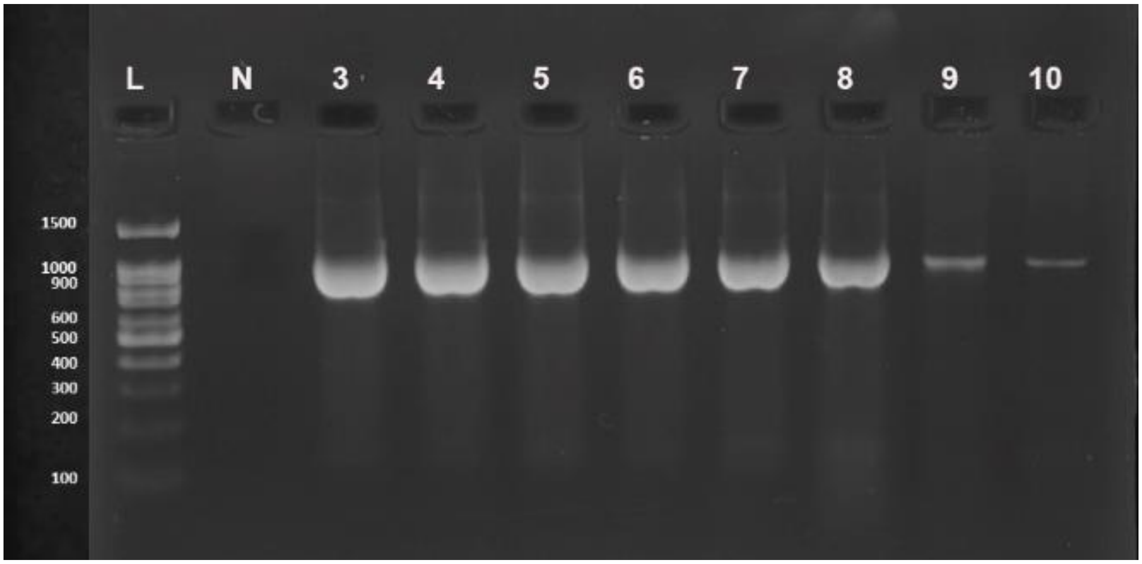

Supplementary Figure S16. PCR gel electrophoresis showing amplification of the *16S rDNA* gene (~956 bp) in *Pseudomonas aeruginosa* isolates. Lane L: 100 bp DNA ladder; Lane N: negative control; Lanes 3–10: amplified products from selected isolates. Clear bands of expected size confirm successful amplification and molecular identification.

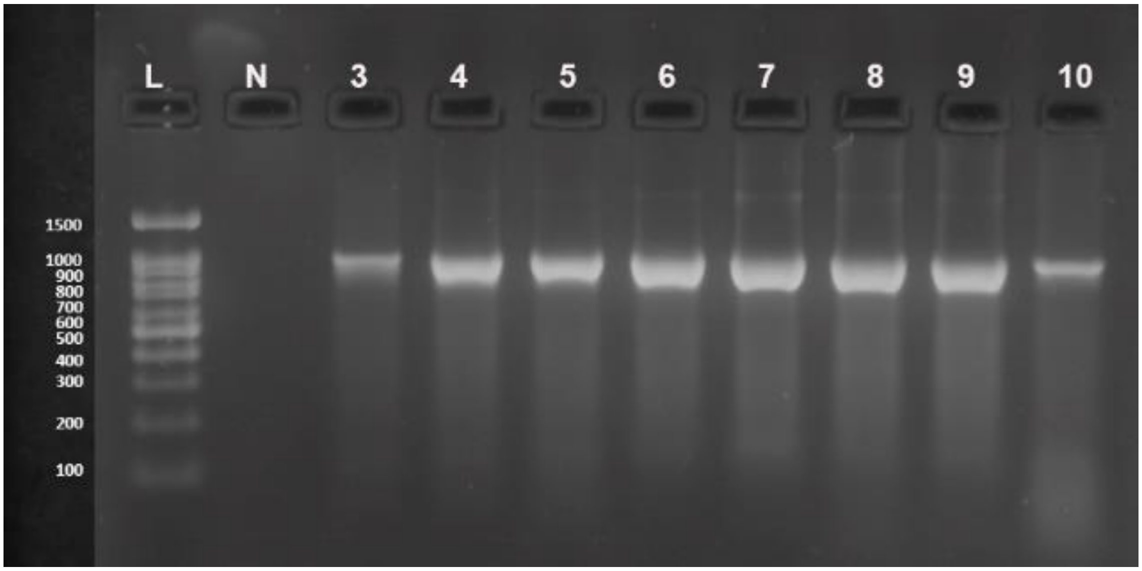

Supplementary Figures S17. PCR gel electrophoresis showing amplification of the *16S rDNA* gene (~956 bp) in *Pseudomonas aeruginosa* isolates. Lane L: 100 bp DNA ladder; Lane N: negative control; Lanes 3–10: amplified products from selected isolates. Clear bands of expected size confirm successful amplification and molecular identification.

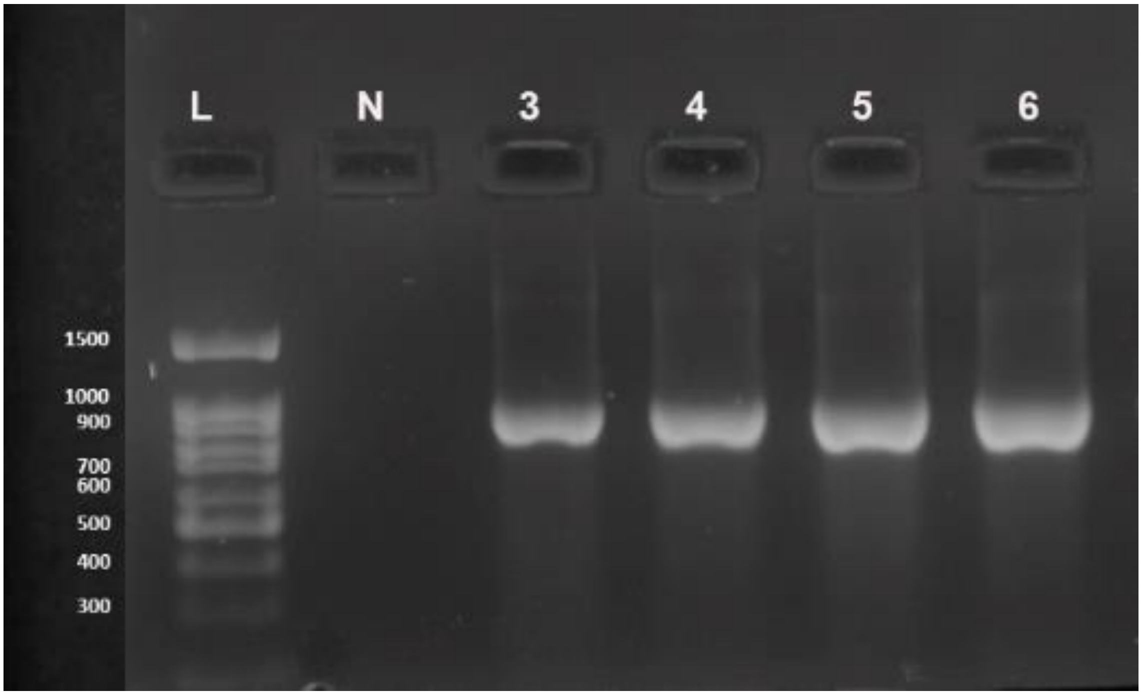

Supplementary Figure S18. PCR gel electrophoresis showing amplification of the *16S rDNA* gene (~956 bp) in *Pseudomonas aeruginosa* isolates. Lane L: 100 bp DNA ladder; Lane N: negative control; Lanes 3–6: amplified products from selected isolates. Clear bands of expected size confirm successful amplification and molecular identification.

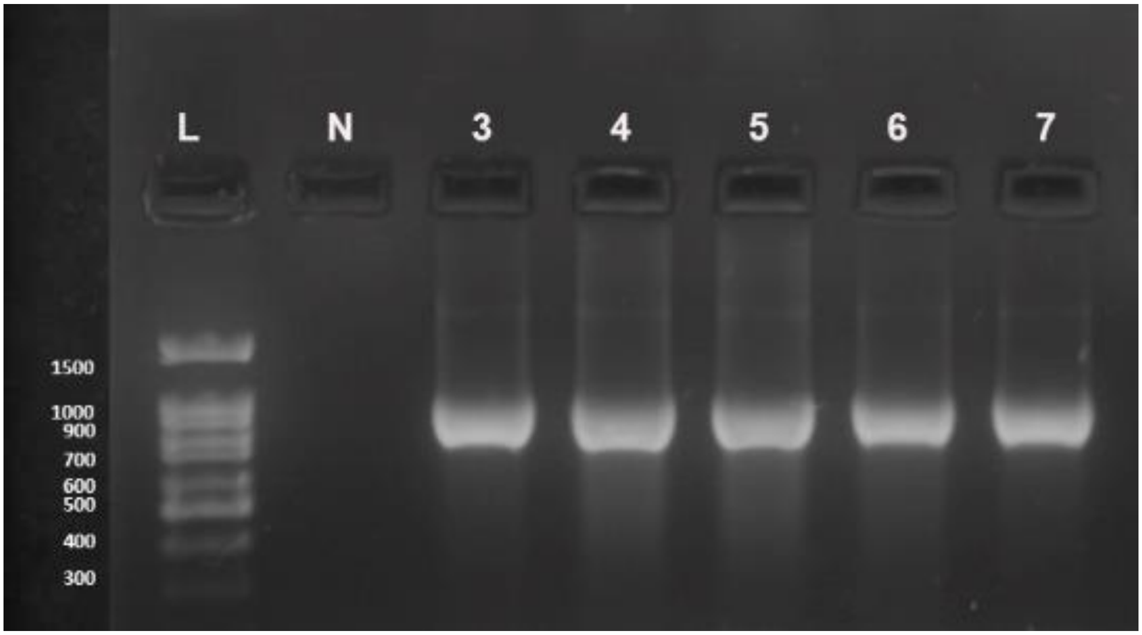

Supplementary Figure S19. PCR gel electrophoresis showing amplification of the *16S rDNA* gene (~956 bp) in *Pseudomonas aeruginosa* isolates. Lane L: 100 bp DNA ladder; Lane N: negative control; Lanes 3–7: amplified products from selected isolates. Clear bands of expected size confirm successful amplification and molecular identification.

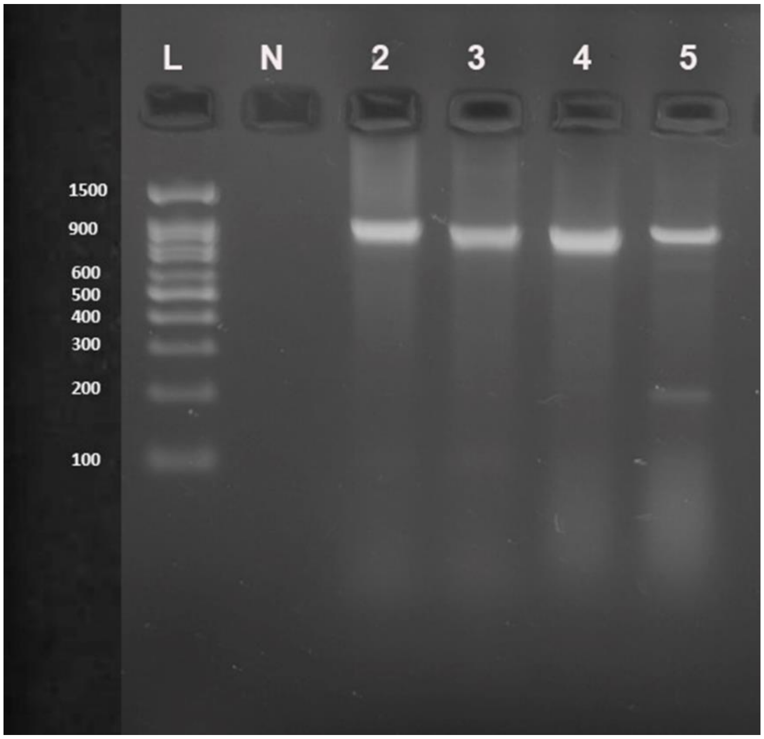

Supplementary Figure S20. PCR gel electrophoresis showing amplification of the *16S rDNA* gene (~956 bp) in *Pseudomonas aeruginosa isolates*. Lane L: 100 bp DNA ladder; Lane N: negative control; Lanes 2–5: amplified products from selected isolates. Clear bands of expected size confirm successful amplification and molecular identification.

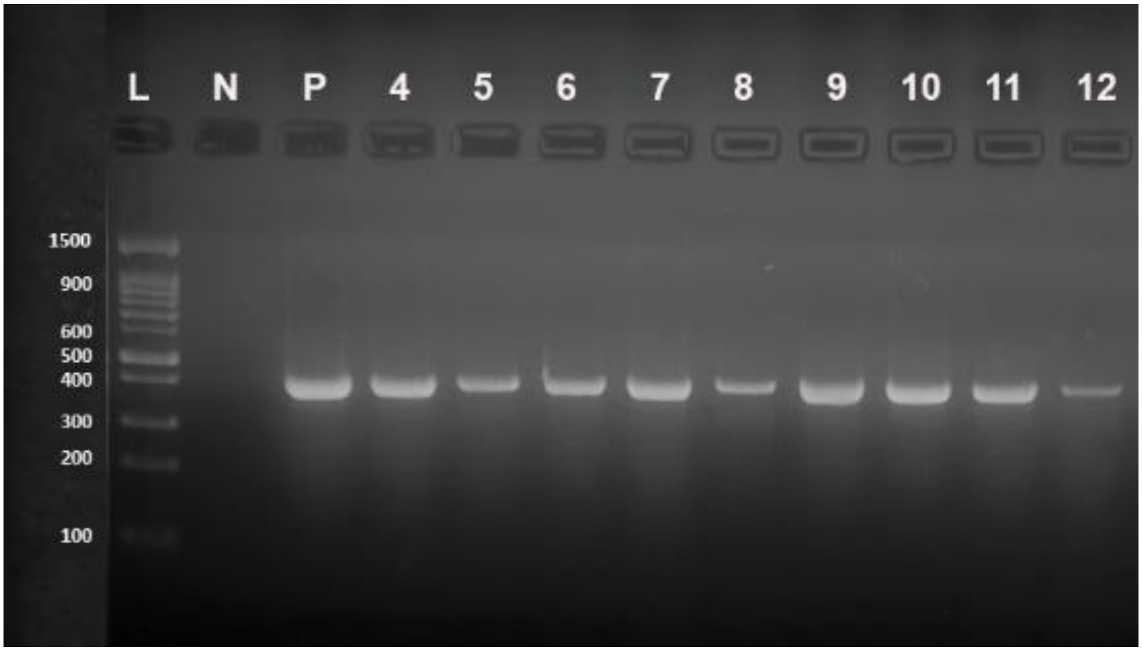

Supplementary Figures S21. PCR gel electrophoresis showing amplification of the bla_{VIM} gene (~390 bp) in *Pseudomonas aeruginosa* isolates. Lane L: 100 bp DNA ladder; Lane N: negative control; Lane P: positive control; Lanes 4–12: amplified products from selected isolates. Clear bands of expected size confirm successful amplification and molecular identification.

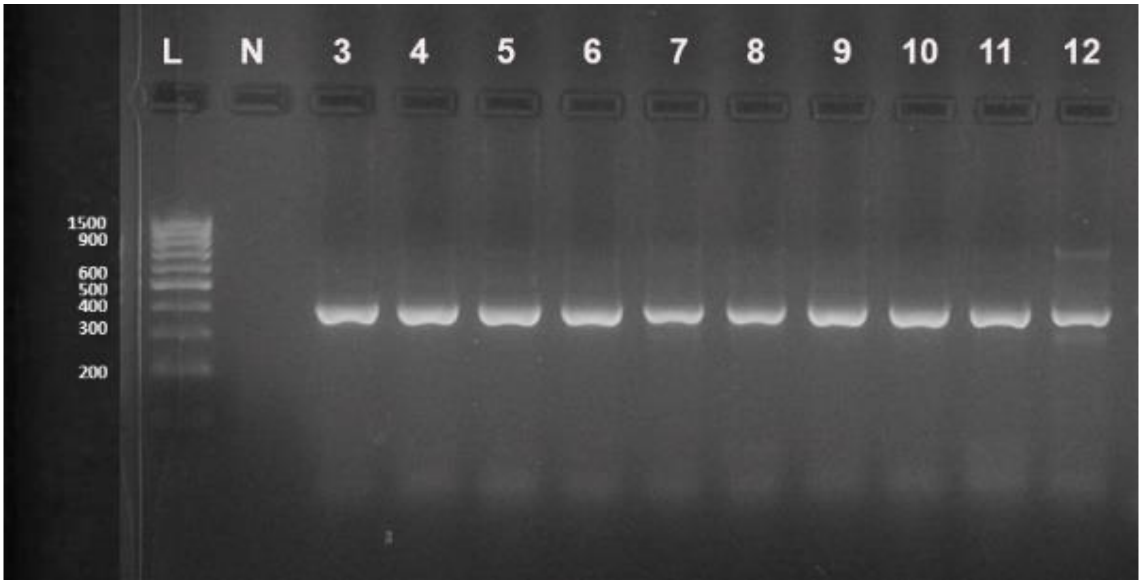

Supplementary Figures S22. PCR gel electrophoresis showing amplification of the *bla*$_{VIM}$ gene (~390 bp) in *Pseudomonas aeruginosa* isolates. Lane L: 100 bp DNA ladder; Lane N: negative control; Lanes 3–12: amplified products from selected isolates. Clear bands of expected size confirm successful amplification and molecular identification.

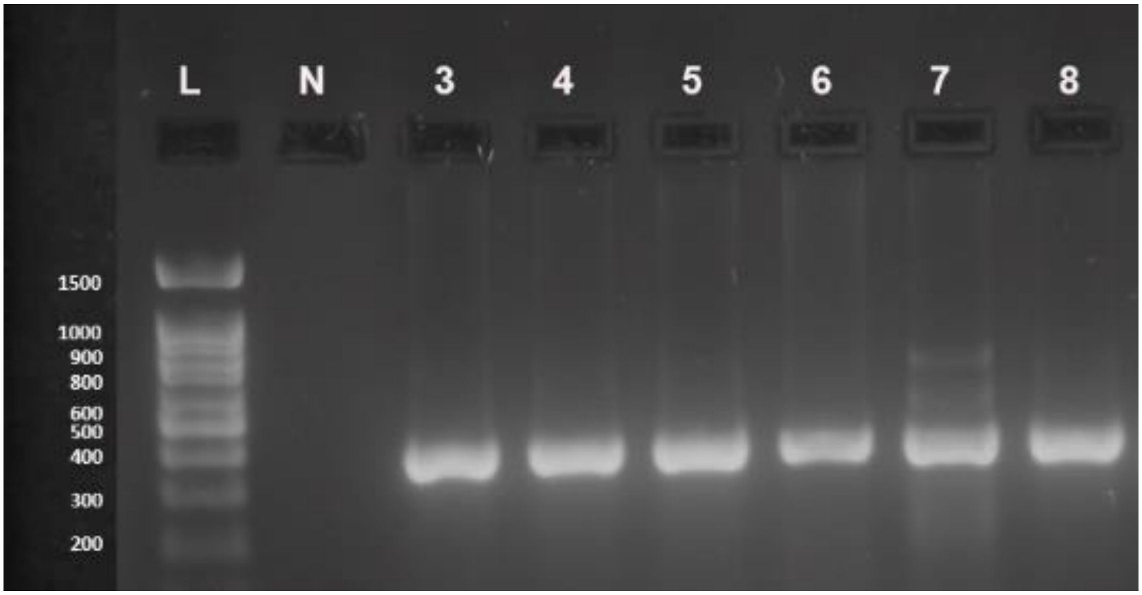

Supplementary Figures S23. PCR gel electrophoresis showing amplification of the *bla*$_{VIM}$ gene (~390 bp) in *Pseudomonas aeruginosa* isolates. Lane L: 100 bp DNA ladder; Lane N: negative control; Lanes 3–8: amplified products from selected isolates. Clear bands of expected size confirm successful amplification and molecular identification.

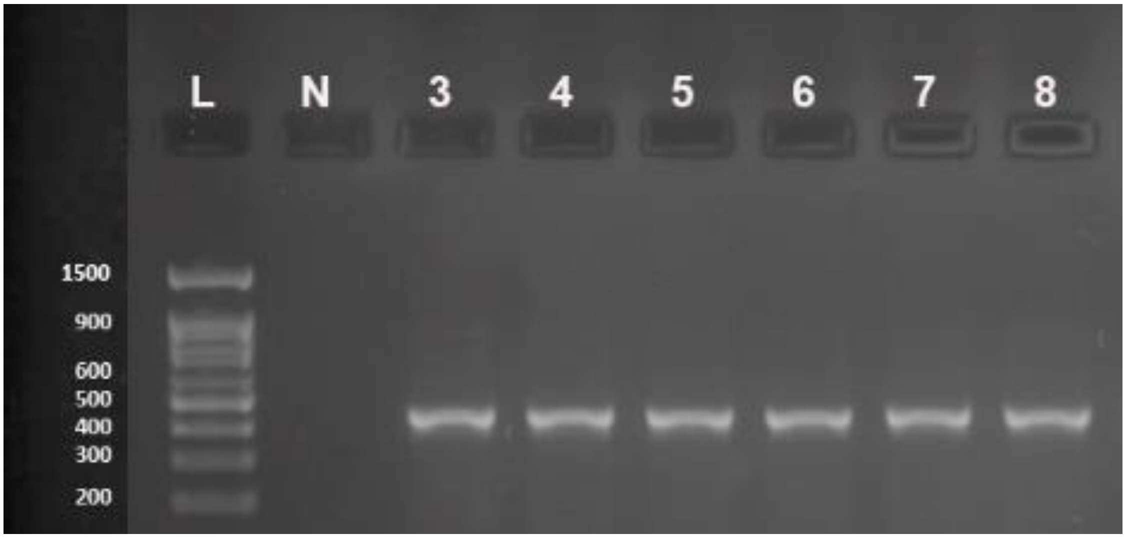

Supplementary Figures S24. PCR gel electrophoresis showing amplification of the *bla*$_{VIM}$ gene (~390 bp) in *Pseudomonas aeruginosa* isolates. Lane L: 100 bp DNA ladder; Lane N: negative control; Lanes 3–8: amplified products from selected isolates. Clear bands of expected size confirm successful amplification and molecular identification.

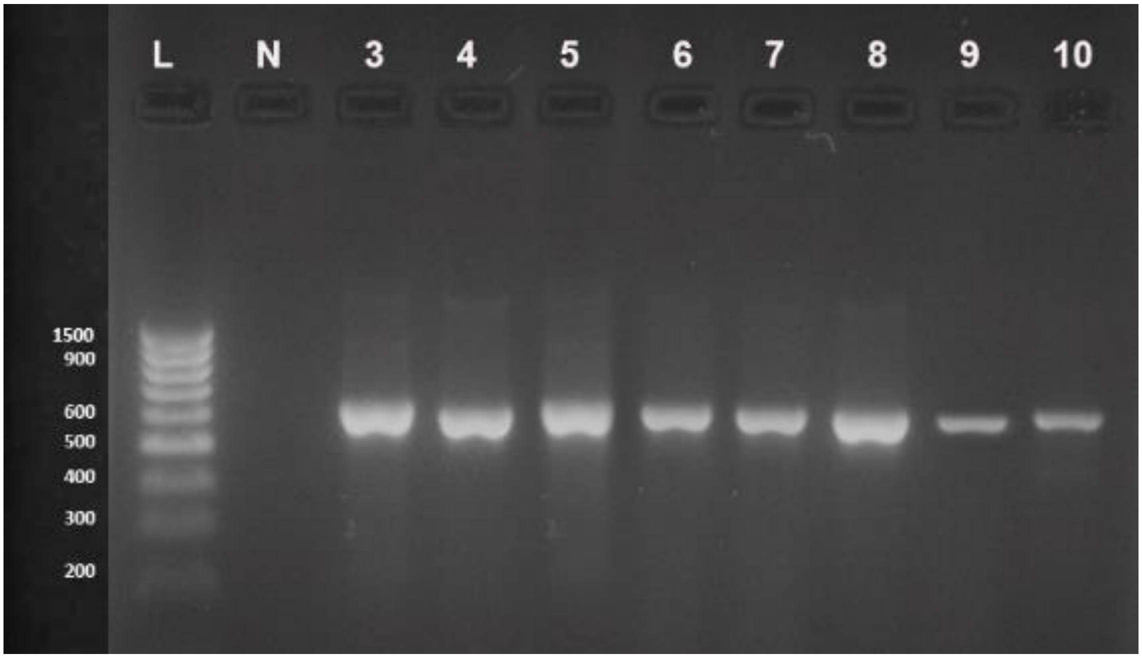

Supplementary Figures S25. PCR gel electrophoresis showing amplification of the *bla*$_{NDM}$ gene (~621 bp) in *Pseudomonas aeruginosa* isolates. Lane L: 100 bp DNA ladder; Lane N: negative control; Lanes 3–10: amplified products from selected isolates. Clear bands of expected size confirm successful amplification and molecular identification**.**

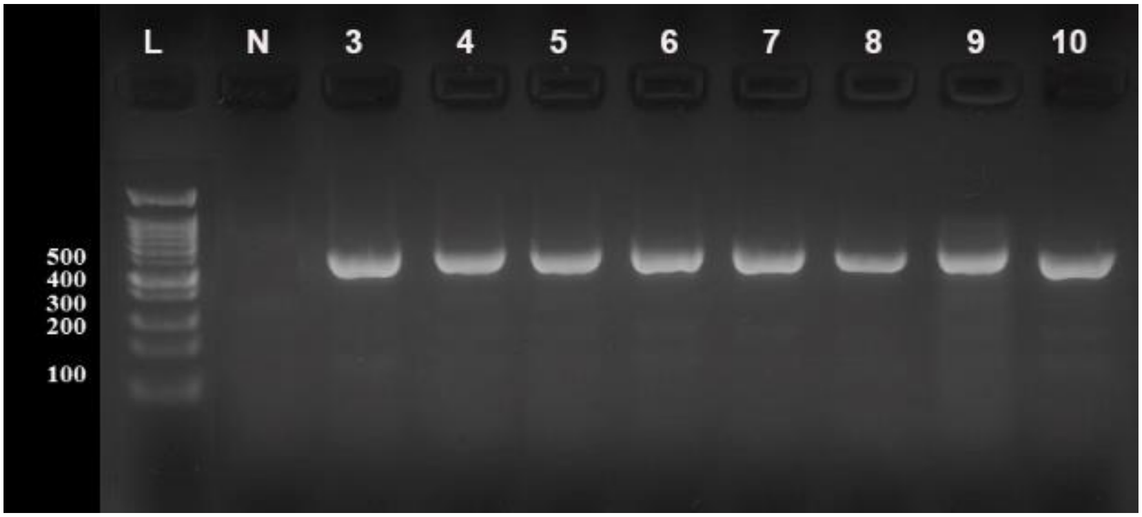

Supplementary Figures S26. PCR gel electrophoresis showing amplification of the *bla*$_{NDM}$ gene (~621 bp) in *Pseudomonas aeruginosa* isolates. Lane L: 100 bp DNA ladder; Lane N: negative control; Lanes 3–10: amplified products from selected isolates. Clear bands of expected size confirm successful amplification and molecular identification.
