# Supplementary material for: Molecular detection of blaVIM and blaNDM in multidrug-resistant Pseudomonas aeruginosa from cancer and burn patients in Erbil, Iraq
Source: Front Microbiol. 2025 Sep 15;16:1672531. doi: 10.3389/fmicb.2025.1672531 (PMC12477123; doi:10.3389/fmicb.2025.1672531)
Supplement: Supplementary file 1 [file Data_Sheet_1.zip › latest_supplementary_material file/Supplementary_Tables/Supplementary_Table_S1.docx]

| **Target Gene** | **Primer Sequence (5′–3′)** | **Product Size (bp)** | **Annealing Temp (°C)** | **Initial Denaturation** | **Denaturation** | **Annealing** | **Extension** | **Final Extension** | **No. of Cycles** | **Reference** |
| --- | --- | --- | --- | --- | --- | --- | --- | --- | --- | --- |
| ***bla_VIM_*** | F: GATGGTGTTTGGTCGCATA R: CGAATGCGCAGCACCAG | 390 | 57 °C for 30 s | 94 °C for 5 min | 94 °C for 30 s | 57 °C for 30 s | 72 °C for 1 min | 72 °C for 8 min | 35 | (Kazemian et al., 2019) |
| ***bla_NDM_*** | F: GGTTTGGCGATCTGGTTTTC R: CGGAATGGCTCATCACGATC | 621 | 57 °C | 95 °C for 5 min | 94 °C for 30 s | 57 °C for 30 s | 72 °C for 1 min | 72 °C for 10 min | 35 | (Kazemian et al., 2019) |
| ***16S rDNA*** | F: GGGGGATCTTCGGACCTCA R: TCCTTAGAGTGCCCACCCG | 956 | 58 °C | 95 °C for 5 min | 95 °C for 30 s | 58 °C for 30 s | 72 °C for 1 min | 72 °C for 5 min | 30 | (Jarjees et al., 2021) |

**Supplementary Table S1:** Detailed thermocycling conditions and primer sequences used in this study.

Kazemian, H., Heidari, H., Ghanavati, R., Ghafourian, S., Yazdani, F., Sadeghifard, N., Valadbeigi, H., Maleki, A. & Pakzad, I. 2019. Phenotypic and Genotypic Characterization of ESBL-, AmpC-, and Carbapenemase-Producing *Klebsiella pneumonia*e and *Escherichia coli* Isolates. *Med Princ Pract,* 28**,** 547-551.

Jarjees, K., Jarjees, R. & Qader, G. 2021. Detection of blaCTX-M Genes among Extended Spectrum Beta Lactamase producing *Pseudomonas aeruginosa* isolated from Clinical Specimens in Erbil. *Indian Journal of Pharmaceutical Sciences,* 83. https://doi.org/10.36468/pharmaceutical-sciences.spl.361
