# Supplementary material for: Molecular detection of blaVIM and blaNDM in multidrug-resistant Pseudomonas aeruginosa from cancer and burn patients in Erbil, Iraq
Source: Front Microbiol. 2025 Sep 15;16:1672531. doi: 10.3389/fmicb.2025.1672531 (PMC12477123; doi:10.3389/fmicb.2025.1672531)
Supplement: Supplementary file 1 [file Data_Sheet_1.zip › latest_supplementary_material file/Supplementary_Tables/Supplementary_Table_S2.docx]

**Supplementary Table 2.** Laboratory Instruments and Chemicals.

| **Category** | **Item** | **Manufacturer** | **Country** |
| --- | --- | --- | --- |
| **Instrument** | Digital Camera | Canon | Japan |
|  | Deionizer | GFL | Germany |
|  | Electrophoresis System | Thermo Fisher Scientific | USA |
|  | Microcentrifuge | Jouan | France |
|  | Micropipettes | Bio-Rad | USA |
|  | Thermal Cycler | Techne | UK |
|  | UV Transilluminator | Syngene | UK |
| **Chemicals** | Agarose | [Norgen Biotek Corp.](https://norgenbiotek.com/) | Canada |
|  | DNase/RNase-free water | Promega | USA |
|  | DNA Safe Stain | Solar Bio | China |
|  | Ethanol | Scharlau | European |
|  | 10X TBE buffer | Promega | USA |
