# Supplementary material for: Molecular detection of blaVIM and blaNDM in multidrug-resistant Pseudomonas aeruginosa from cancer and burn patients in Erbil, Iraq
Source: Front Microbiol. 2025 Sep 15;16:1672531. doi: 10.3389/fmicb.2025.1672531 (PMC12477123; doi:10.3389/fmicb.2025.1672531)
Supplement: Supplementary file 1 [file Data_Sheet_1.zip › latest_supplementary_material file/Supplementary_Tables/Supplementary_Table_S3.docx]

**Supplementary Table 3.** Burn injury and admission characteristics among burn patients.

| **Variable** | **n (%) or Median (Range)** |
| --- | --- |
| Total patients | 16 (100.0%) |
| **Mechanism of burn** |  |
| Fire / Flame | 14 (87.5%) |
| Electrical burn | 2 (12.5%) |
| **Degree of burn (highest per patient)** |  |
| 1^st^ degree | 1 (6.3%) |
| 2^nd^ degree | 12 (75.0%) |
| 3^rd^ degree | 3 (18.8%) |
| **Median TBSA burned (%) [range]** | 31.8% (8–90%) |
| **Median TBSA burned (%) [IQR]** | 31.8% (19.75–55.75%) |
| **Regions affected*** |  |
| Head | 12 (75.0%) |
| Neck | 10 (62.5%) |
| Anterior trunk | 11 (68.8%) |
| Posterior trunk | 4 (25.0%) |
| Genitalia | 3 (18.8%) |
| Buttocks | 3 (18.8%) |
| Upper limbs | 14 (87.5%) |
| Lower limbs | 8 (50.0%) |
| **Season of injury** |  |
| Autumn | 8 (50.0%) |
| Winter | 5 (31.3%) |
| Spring | 3 (18.8%) |
| **Place of occurrence** |  |
| Home | 3 (18.8%) |
| Industrial/Workplace | 2 (12.5%) |
| Street/Highway | 2 (12.5%) |
| Farm | 1 (6.3%) |
| Public Building | 1 (6.3%) |
| Unspecified | 7 (43.8%) |
| **Indication for admission** |  |
| TBSA burned & degree | 14 (87.5%) |
| Specific area involvement | 2 (12.5%) |

*Regions affected: Any involvement (counted per patient, multiple regions possible). TBSA = Total Body Surface Area.*
