# Supplementary material for: Molecular detection of blaVIM and blaNDM in multidrug-resistant Pseudomonas aeruginosa from cancer and burn patients in Erbil, Iraq
Source: Front Microbiol. 2025 Sep 15;16:1672531. doi: 10.3389/fmicb.2025.1672531 (PMC12477123; doi:10.3389/fmicb.2025.1672531)
Supplement: Supplementary file 1 [file Data_Sheet_1.zip › latest_supplementary_material file/Supplementary_Tables/Supplementary_Table_S4.docx]

| **Sample ID** | **Antibiotic Tested** | **AST Result (Zone Diameter or MIC)** | **Interpretation (R/I/S)** | **AST Method (Vitek2/Manual)** |
| --- | --- | --- | --- | --- |
| 1 | Piperacillin/Tazobactam | >= 128 | R | Vitek2 |
| 1 | Ceftazidime/Avibactam | >= 16 | R | Vitek2 |
| 1 | Ceftolozane/Tazobactam | >= 32 | R | Vitek2 |
| 1 | Ceftazidime | >= 64 | R | Vitek2 |
| 1 | Cefepime | >= 32 | R | Vitek2 |
| 1 | Imipenem | >= 16 | R | Vitek2 |
| 1 | Meropenem | >= 16 | R | Vitek2 |
| 1 | Amikacin | >= 64 | R | Vitek2 |
| 1 | Gentamicin | >= 16 | R | Vitek2 |
| 1 | Ciprofloxacin | >= 4 | R | Vitek2 |
| 1 | Colistin | 2 | S | Vitek2 |
| 2 | Piperacillin/Tazobactam | >= 128 | R | Vitek2 |
| 2 | Ceftazidime/Avibactam | 17 mm | R | Manual |
| 2 | Ceftolozane/Tazobactam | 11 mm | R | Manual |
| 2 | Ceftazidime | >= 64 | R | Vitek2 |
| 2 | Cefepime | >= 32 | R | Vitek2 |
| 2 | Imipenem | >= 16 | R | Vitek2 |
| 2 | Meropenem | >= 16 | R | Vitek2 |
| 2 | Amikacin | >= 64 | R | Vitek2 |
| 2 | Gentamicin | >= 16 | R | Vitek2 |
| 2 | Ciprofloxacin | >= 4 | R | Vitek2 |
| 2 | Colistin | — | — | — |
| 3 | Piperacillin/Tazobactam | <= 4 | S | Vitek2 |
| 3 | Ceftazidime/Avibactam | 2 | S | Vitek2 |
| 3 | Ceftolozane/Tazobactam | 0.5 | S | Vitek2 |
| 3 | Ceftazidime | 2 | S | Vitek2 |
| 3 | Cefepime | 2 | S | Vitek2 |
| 3 | Imipenem | 2 | S | Vitek2 |
| 3 | Meropenem | 4 | I | Vitek2 |
| 3 | Amikacin | <= 1 | S | Vitek2 |
| 3 | Gentamicin | <= 1 | S | Vitek2 |
| 3 | Ciprofloxacin | 0.25 | S | Vitek2 |
| 3 | Colistin | 1 | S | Vitek2 |
| 4 | Piperacillin/Tazobactam | >= 128 | R | Vitek2 |
| 4 | Ceftazidime/Avibactam | >= 16 | R | Vitek2 |
| 4 | Ceftolozane/Tazobactam | >= 32 | R | Vitek2 |
| 4 | Ceftazidime | >= 64 | R | Vitek2 |
| 4 | Cefepime | >= 32 | R | Vitek2 |
| 4 | Imipenem | 2 | S | Vitek2 |
| 4 | Meropenem | 4 | I | Vitek2 |
| 4 | Amikacin | 4 | S | Vitek2 |
| 4 | Gentamicin | <= 1 | S | Vitek2 |
| 4 | Ciprofloxacin | 0.25 | S | Vitek2 |
| 4 | Colistin | 2 | S | Vitek2 |
| 5 | Piperacillin/Tazobactam | >= 128 | R | Vitek2 |
| 5 | Ceftazidime/Avibactam | >= 16 | R | Vitek2 |
| 5 | Ceftolozane/Tazobactam | >= 32 | R | Vitek2 |
| 5 | Ceftazidime | >= 64 | R | Vitek2 |
| 5 | Cefepime | >= 32 | R | Vitek2 |
| 5 | Imipenem | >= 16 | R | Vitek2 |
| 5 | Meropenem | >= 16 | R | Vitek2 |
| 5 | Amikacin | >= 64 | R | Vitek2 |
| 5 | Gentamicin | >= 16 | R | Vitek2 |
| 5 | Ciprofloxacin | >= 4 | R | Vitek2 |
| 5 | Colistin | 2 | S | Vitek2 |
| 6 | Piperacillin/Tazobactam | >= 128 | R | Vitek2 |
| 6 | Ceftazidime/Avibactam | 16 mm | R | Manual |
| 6 | Ceftolozane/Tazobactam | 15 mm | R | Manual |
| 6 | Ceftazidime | >= 64 | R | Vitek2 |
| 6 | Cefepime | >= 32 | R | Vitek2 |
| 6 | Imipenem | 2 | S | Vitek2 |
| 6 | Meropenem | 1 | S | Vitek2 |
| 6 | Amikacin | 4 | S | Vitek2 |
| 6 | Gentamicin | <= 1 | S | Vitek2 |
| 6 | Ciprofloxacin | 0.25 | S | Vitek2 |
| 6 | Colistin | — | — | — |
| 7 | Piperacillin/Tazobactam | 16 | S | Vitek2 |
| 7 | Ceftazidime/Avibactam | 16 mm | S | Manual |
| 7 | Ceftolozane/Tazobactam | 15 mm | S | Manual |
| 7 | Ceftazidime | 4 | S | Vitek2 |
| 7 | Cefepime | 2 | S | Vitek2 |
| 7 | Imipenem | 2 | S | Vitek2 |
| 7 | Meropenem | 4 | I | Vitek2 |
| 7 | Amikacin | <= 2 | S | Vitek2 |
| 7 | Gentamicin | >= 16 | R | Vitek2 |
| 7 | Ciprofloxacin | >= 4 | R | Vitek2 |
| 7 | Colistin | 2 | S | Vitek2 |
| 8 | Piperacillin/Tazobactam | >= 128 | R | Vitek2 |
| 8 | Ceftazidime/Avibactam | >= 16 | R | Vitek2 |
| 8 | Ceftolozane/Tazobactam | >= 32 | R | Vitek2 |
| 8 | Ceftazidime | >= 64 | R | Vitek2 |
| 8 | Cefepime | >= 32 | R | Vitek2 |
| 8 | Imipenem | 2 | S | Vitek2 |
| 8 | Meropenem | 2 | S | Vitek2 |
| 8 | Amikacin | 4 | S | Vitek2 |
| 8 | Gentamicin | 2 | S | Vitek2 |
| 8 | Ciprofloxacin | 0.12 | S | Vitek2 |
| 8 | Colistin | 2 | S | Vitek2 |
| 9 | Piperacillin/Tazobactam | >= 128 | R | Vitek2 |
| 9 | Ceftazidime/Avibactam | >= 16 | R | Vitek2 |
| 9 | Ceftolozane/Tazobactam | >= 32 | R | Vitek2 |
| 9 | Ceftazidime | >= 64 | R | Vitek2 |
| 9 | Cefepime | >= 32 | R | Vitek2 |
| 9 | Imipenem | >= 16 | R | Vitek2 |
| 9 | Meropenem | >= 16 | R | Vitek2 |
| 9 | Amikacin | >= 64 | R | Vitek2 |
| 9 | Gentamicin | >= 16 | R | Vitek2 |
| 9 | Ciprofloxacin | >= 4 | R | Vitek2 |
| 9 | Colistin | 2 | S | Vitek2 |
| 10 | Piperacillin/Tazobactam | >= 128 | R | Vitek2 |
| 10 | Ceftazidime/Avibactam | >= 16 | R | Vitek2 |
| 10 | Ceftolozane/Tazobactam | >= 32 | R | Vitek2 |
| 10 | Ceftazidime | >= 64 | R | Vitek2 |
| 10 | Cefepime | >= 32 | R | Vitek2 |
| 10 | Imipenem | >= 16 | R | Vitek2 |
| 10 | Meropenem | >= 16 | R | Vitek2 |
| 10 | Amikacin | <= 1 | S | Vitek2 |
| 10 | Gentamicin | <= 1 | S | Vitek2 |
| 10 | Ciprofloxacin | 0.12 | S | Vitek2 |
| 10 | Colistin | 2 | S | Vitek2 |
| 11 | Piperacillin/Tazobactam | 32 | I | Vitek2 |
| 11 | Ceftazidime/Avibactam | 2 | S | Vitek2 |
| 11 | Ceftolozane/Tazobactam | 1 | S | Vitek2 |
| 11 | Ceftazidime | 2 | S | Vitek2 |
| 11 | Cefepime | 2 | S | Vitek2 |
| 11 | Imipenem | 2 | S | Vitek2 |
| 11 | Meropenem | 0.5 | S | Vitek2 |
| 11 | Amikacin | 4 | S | Vitek2 |
| 11 | Gentamicin | <= 1 | S | Vitek2 |
| 11 | Ciprofloxacin | 0.12 | S | Vitek2 |
| 11 | Colistin | 1 | S | Vitek2 |
| 12 | Piperacillin/Tazobactam | 8 | S | Vitek2 |
| 12 | Ceftazidime/Avibactam | 2 | S | Vitek2 |
| 12 | Ceftolozane/Tazobactam | 0.5 | S | Vitek2 |
| 12 | Ceftazidime | 2 | S | Vitek2 |
| 12 | Cefepime | 2 | S | Vitek2 |
| 12 | Imipenem | 2 | S | Vitek2 |
| 12 | Meropenem | 1 | S | Vitek2 |
| 12 | Amikacin | 4 | S | Vitek2 |
| 12 | Gentamicin | <= 1 | S | Vitek2 |
| 12 | Ciprofloxacin | 0.12 | S | Vitek2 |
| 12 | Colistin | 2 | S | Vitek2 |
| 13 | Piperacillin/Tazobactam | >= 128 | R | Vitek2 |
| 13 | Ceftazidime/Avibactam | 16 mm | R | Manual |
| 13 | Ceftolozane/Tazobactam | 11 mm | R | Manual |
| 13 | Ceftazidime | >= 64 | R | Vitek2 |
| 13 | Cefepime | >= 64 | R | Vitek2 |
| 13 | Imipenem | >= 16 | R | Vitek2 |
| 13 | Meropenem | >= 16 | R | Vitek2 |
| 13 | Amikacin | >= 64 | R | Vitek2 |
| 13 | Gentamicin | >= 16 | R | Vitek2 |
| 13 | Ciprofloxacin | >= 4 | R | Vitek2 |
| 13 | Colistin | — | — | — |
| 14 | Piperacillin/Tazobactam | 8 | S | Vitek2 |
| 14 | Ceftazidime/Avibactam | 4 | S | Vitek2 |
| 14 | Ceftolozane/Tazobactam | 1 | S | Vitek2 |
| 14 | Ceftazidime | 8 | S | Vitek2 |
| 14 | Cefepime | 8 | S | Vitek2 |
| 14 | Imipenem | 2 | S | Vitek2 |
| 14 | Meropenem | 2 | S | Vitek2 |
| 14 | Amikacin | 4 | S | Vitek2 |
| 14 | Gentamicin | <= 1 | S | Vitek2 |
| 14 | Ciprofloxacin | 0.5 | S | Vitek2 |
| 14 | Colistin | <= 0.5 | S | Vitek2 |
| 15 | Piperacillin/Tazobactam | >= 128 | R | Vitek2 |
| 15 | Ceftazidime/Avibactam | >= 16 | R | Vitek2 |
| 15 | Ceftolozane/Tazobactam | >= 32 | R | Vitek2 |
| 15 | Ceftazidime | >= 64 | R | Vitek2 |
| 15 | Cefepime | >= 32 | R | Vitek2 |
| 15 | Imipenem | 8 | R | Vitek2 |
| 15 | Meropenem | >= 16 | R | Vitek2 |
| 15 | Amikacin | >= 64 | R | Vitek2 |
| 15 | Gentamicin | >= 16 | R | Vitek2 |
| 15 | Ciprofloxacin | >= 4 | R | Vitek2 |
| 15 | Colistin | 10 mm | S | Manual |
| 16 | Piperacillin/Tazobactam | >= 128 | R | Vitek2 |
| 16 | Ceftazidime/Avibactam | >= 16 | R | Vitek2 |
| 16 | Ceftolozane/Tazobactam | >= 32 | R | Vitek2 |
| 16 | Ceftazidime | >= 64 | R | Vitek2 |
| 16 | Cefepime | >= 32 | R | Vitek2 |
| 16 | Imipenem | >= 16 | R | Vitek2 |
| 16 | Meropenem | >= 16 | R | Vitek2 |
| 16 | Amikacin | >= 64 | R | Vitek2 |
| 16 | Gentamicin | >= 16 | R | Vitek2 |
| 16 | Ciprofloxacin | >= 4 | R | Vitek2 |
| 16 | Colistin | 12 mm | S | Manual |
| 17 | Piperacillin/Tazobactam | >= 128 | R | Vitek2 |
| 17 | Ceftazidime/Avibactam | >= 16 | R | Vitek2 |
| 17 | Ceftolozane/Tazobactam | >= 32 | R | Vitek2 |
| 17 | Ceftazidime | >= 64 | R | Vitek2 |
| 17 | Cefepime | >= 32 | R | Vitek2 |
| 17 | Imipenem | 8 | R | Vitek2 |
| 17 | Meropenem | >= 16 | R | Vitek2 |
| 17 | Amikacin | >= 64 | R | Vitek2 |
| 17 | Gentamicin | >= 16 | R | Vitek2 |
| 17 | Ciprofloxacin | >= 4 | R | Vitek2 |
| 17 | Colistin | 12 mm | S | Manual |
| 18 | Piperacillin/Tazobactam | >= 128 | R | Vitek2 |
| 18 | Ceftazidime/Avibactam | >= 16 | R | Vitek2 |
| 18 | Ceftolozane/Tazobactam | >= 32 | R | Vitek2 |
| 18 | Ceftazidime | >= 64 | R | Vitek2 |
| 18 | Cefepime | >= 32 | R | Vitek2 |
| 18 | Imipenem | 8 | R | Vitek2 |
| 18 | Meropenem | >= 16 | R | Vitek2 |
| 18 | Amikacin | >= 64 | R | Vitek2 |
| 18 | Gentamicin | >= 16 | R | Vitek2 |
| 18 | Ciprofloxacin | >= 4 | R | Vitek2 |
| 18 | Colistin | 10 mm | S | Manual |
| 19 | Piperacillin/Tazobactam | >= 128 | R | Vitek2 |
| 19 | Ceftazidime/Avibactam | >= 16 | R | Vitek2 |
| 19 | Ceftolozane/Tazobactam | >= 32 | R | Vitek2 |
| 19 | Ceftazidime | >= 64 | R | Vitek2 |
| 19 | Cefepime | >= 32 | R | Vitek2 |
| 19 | Imipenem | 8 | R | Vitek2 |
| 19 | Meropenem | >= 16 | R | Vitek2 |
| 19 | Amikacin | >= 64 | R | Vitek2 |
| 19 | Gentamicin | >= 16 | R | Vitek2 |
| 19 | Ciprofloxacin | >= 4 | R | Vitek2 |
| 19 | Colistin | 12 mm | S | Manual |
| 20 | Piperacillin/Tazobactam | >= 128 | R | Vitek2 |
| 20 | Ceftazidime/Avibactam | >= 16 | R | Vitek2 |
| 20 | Ceftolozane/Tazobactam | >= 32 | R | Vitek2 |
| 20 | Ceftazidime | >= 64 | R | Vitek2 |
| 20 | Cefepime | >= 32 | R | Vitek2 |
| 20 | Imipenem | >= 16 | R | Vitek2 |
| 20 | Meropenem | >= 16 | R | Vitek2 |
| 20 | Amikacin | >= 64 | R | Vitek2 |
| 20 | Gentamicin | >= 16 | R | Vitek2 |
| 20 | Ciprofloxacin | >= 4 | R | Vitek2 |
| 20 | Colistin | 10 mm | S | Manual |
| 21 | Piperacillin/Tazobactam | >= 128 | R | Vitek2 |
| 21 | Ceftazidime/Avibactam | >= 16 | R | Vitek2 |
| 21 | Ceftolozane/Tazobactam | >= 32 | R | Vitek2 |
| 21 | Ceftazidime | >= 64 | R | Vitek2 |
| 21 | Cefepime | >= 32 | R | Vitek2 |
| 21 | Imipenem | >= 16 | R | Vitek2 |
| 21 | Meropenem | >= 16 | R | Vitek2 |
| 21 | Amikacin | >= 64 | R | Vitek2 |
| 21 | Gentamicin | >= 16 | R | Vitek2 |
| 21 | Ciprofloxacin | >= 4 | R | Vitek2 |
| 21 | Colistin | 12 mm | S | Manual |
| 22 | Piperacillin/Tazobactam | >= 128 | R | Vitek2 |
| 22 | Ceftazidime/Avibactam | >= 16 | R | Vitek2 |
| 22 | Ceftolozane/Tazobactam | >= 32 | R | Vitek2 |
| 22 | Ceftazidime | >= 64 | R | Vitek2 |
| 22 | Cefepime | >= 32 | R | Vitek2 |
| 22 | Imipenem | >= 16 | R | Vitek2 |
| 22 | Meropenem | >= 16 | R | Vitek2 |
| 22 | Amikacin | >= 64 | R | Vitek2 |
| 22 | Gentamicin | >= 16 | R | Vitek2 |
| 22 | Ciprofloxacin | >= 4 | R | Vitek2 |
| 22 | Colistin | 15 mm | S | Manual |
| 23 | Piperacillin/Tazobactam | >= 128 | R | Vitek2 |
| 23 | Ceftazidime/Avibactam | >= 16 | R | Vitek2 |
| 23 | Ceftolozane/Tazobactam | >= 32 | R | Vitek2 |
| 23 | Ceftazidime | >= 64 | R | Vitek2 |
| 23 | Cefepime | >= 32 | R | Vitek2 |
| 23 | Imipenem | 8 | R | Vitek2 |
| 23 | Meropenem | >= 16 | R | Vitek2 |
| 23 | Amikacin | >= 64 | R | Vitek2 |
| 23 | Gentamicin | >= 16 | R | Vitek2 |
| 23 | Ciprofloxacin | >= 4 | R | Vitek2 |
| 23 | Colistin | 12 mm | S | Manual |
| 24 | Piperacillin/Tazobactam | >= 128 | R | Vitek2 |
| 24 | Ceftazidime/Avibactam | >= 16 | R | Vitek2 |
| 24 | Ceftolozane/Tazobactam | >= 32 | R | Vitek2 |
| 24 | Ceftazidime | >= 64 | R | Vitek2 |
| 24 | Cefepime | >= 32 | R | Vitek2 |
| 24 | Imipenem | 8 | R | Vitek2 |
| 24 | Meropenem | >= 16 | R | Vitek2 |
| 24 | Amikacin | >= 64 | R | Vitek2 |
| 24 | Gentamicin | >= 16 | R | Vitek2 |
| 24 | Ciprofloxacin | >= 4 | R | Vitek2 |
| 24 | Colistin | 12 mm | S | Manual |
| 25 | Piperacillin/Tazobactam | >= 128 | R | Vitek2 |
| 25 | Ceftazidime/Avibactam | 8 | R | Vitek2 |
| 25 | Ceftolozane/Tazobactam | 2 | R | Vitek2 |
| 25 | Ceftazidime | >= 64 | R | Vitek2 |
| 25 | Cefepime | >= 16 | R | Vitek2 |
| 25 | Imipenem | >= 16 | R | Vitek2 |
| 25 | Meropenem | >= 16 | R | Vitek2 |
| 25 | Amikacin | 8 | R | Vitek2 |
| 25 | Gentamicin | >= 16 | R | Vitek2 |
| 25 | Ciprofloxacin | >= 4 | R | Vitek2 |
| 25 | Colistin | 10 mm | S | Manual |
| 26 | Piperacillin/Tazobactam | >= 128 | R | Vitek2 |
| 26 | Ceftazidime/Avibactam | >= 16 | R | Vitek2 |
| 26 | Ceftolozane/Tazobactam | >= 32 | R | Vitek2 |
| 26 | Ceftazidime | >= 64 | R | Vitek2 |
| 26 | Cefepime | >= 32 | R | Vitek2 |
| 26 | Imipenem | 8 | R | Vitek2 |
| 26 | Meropenem | >= 16 | R | Vitek2 |
| 26 | Amikacin | >= 64 | R | Vitek2 |
| 26 | Gentamicin | >= 16 | R | Vitek2 |
| 26 | Ciprofloxacin | >= 4 | R | Vitek2 |
| 26 | Colistin | 13 mm | S | Manual |
| 27 | Piperacillin/Tazobactam | >= 128 | R | Vitek2 |
| 27 | Ceftazidime/Avibactam | >= 16 | R | Vitek2 |
| 27 | Ceftolozane/Tazobactam | >= 32 | R | Vitek2 |
| 27 | Ceftazidime | >= 64 | R | Vitek2 |
| 27 | Cefepime | >= 32 | R | Vitek2 |
| 27 | Imipenem | >= 16 | R | Vitek2 |
| 27 | Meropenem | >= 16 | R | Vitek2 |
| 27 | Amikacin | >= 64 | R | Vitek2 |
| 27 | Gentamicin | >= 16 | R | Vitek2 |
| 27 | Ciprofloxacin | >= 4 | R | Vitek2 |
| 27 | Colistin | — | — | — |
| 28 | Piperacillin/Tazobactam | >= 128 | R | Vitek2 |
| 28 | Ceftazidime/Avibactam | >= 16 | R | Vitek2 |
| 28 | Ceftolozane/Tazobactam | >= 32 | R | Vitek2 |
| 28 | Ceftazidime | >= 64 | R | Vitek2 |
| 28 | Cefepime | >= 32 | R | Vitek2 |
| 28 | Imipenem | 8 | R | Vitek2 |
| 28 | Meropenem | >= 16 | R | Vitek2 |
| 28 | Amikacin | >= 64 | R | Vitek2 |
| 28 | Gentamicin | >= 16 | R | Vitek2 |
| 28 | Ciprofloxacin | >= 4 | R | Vitek2 |
| 28 | Colistin | — | — | — |
| 29 | Piperacillin/Tazobactam | >= 128 | R | Vitek2 |
| 29 | Ceftazidime/Avibactam | >= 16 | R | Vitek2 |
| 29 | Ceftolozane/Tazobactam | >= 32 | R | Vitek2 |
| 29 | Ceftazidime | >= 64 | R | Vitek2 |
| 29 | Cefepime | >= 32 | R | Vitek2 |
| 29 | Imipenem | >= 16 | R | Vitek2 |
| 29 | Meropenem | >= 16 | R | Vitek2 |
| 29 | Amikacin | >= 64 | R | Vitek2 |
| 29 | Gentamicin | >= 16 | R | Vitek2 |
| 29 | Ciprofloxacin | >= 4 | R | Vitek2 |
| 29 | Colistin | — | — | — |
| 30 | Piperacillin/Tazobactam | — | — | — |
| 30 | Ceftazidime/Avibactam | — | — | — |
| 30 | Ceftolozane/Tazobactam | — | — | — |
| 30 | Ceftazidime | — | — | — |
| 30 | Cefepime | 30 mm | S | Manual |
| 30 | Imipenem | 36 mm | S | Manual |
| 30 | Meropenem | 34 mm | S | Manual |
| 30 | Amikacin | 26 mm | S | Manual |
| 30 | Gentamicin | 28 mm | S | Manual |
| 30 | Ciprofloxacin | — | — | — |
| 30 | Colistin | 12 mm | S | Manual |
| 31 | Piperacillin/Tazobactam | 64 | I | Vitek2 |
| 31 | Ceftazidime/Avibactam | >= 16 | R | Vitek2 |
| 31 | Ceftolozane/Tazobactam | >= 32 | R | Vitek2 |
| 31 | Ceftazidime | >= 64 | R | Vitek2 |
| 31 | Cefepime | >= 32 | R | Vitek2 |
| 31 | Imipenem | >= 16 | R | Vitek2 |
| 31 | Meropenem | >= 16 | R | Vitek2 |
| 31 | Amikacin | >= 64 | R | Vitek2 |
| 31 | Gentamicin | >= 16 | R | Vitek2 |
| 31 | Ciprofloxacin | >= 4 | R | Vitek2 |
| 31 | Colistin | >= 16 | R | Vitek2 |
| 32 | Piperacillin/Tazobactam | >= 128 | R | Vitek2 |
| 32 | Ceftazidime/Avibactam | >= 16 | R | Vitek2 |
| 32 | Ceftolozane/Tazobactam | >= 32 | R | Vitek2 |
| 32 | Ceftazidime | >= 64 | R | Vitek2 |
| 32 | Cefepime | >= 32 | R | Vitek2 |
| 32 | Imipenem | >= 16 | R | Vitek2 |
| 32 | Meropenem | >= 16 | R | Vitek2 |
| 32 | Amikacin | >= 64 | R | Vitek2 |
| 32 | Gentamicin | >= 16 | R | Vitek2 |
| 32 | Ciprofloxacin | >= 4 | R | Vitek2 |
| 32 | Colistin | 12 mm | S | Manual |
| 33 | Piperacillin/Tazobactam | 64 | I | Vitek2 |
| 33 | Ceftazidime/Avibactam | >= 16 | R | Vitek2 |
| 33 | Ceftolozane/Tazobactam | >= 32 | R | Vitek2 |
| 33 | Ceftazidime | >= 64 | R | Vitek2 |
| 33 | Cefepime | >= 32 | R | Vitek2 |
| 33 | Imipenem | >= 16 | R | Vitek2 |
| 33 | Meropenem | >= 16 | R | Vitek2 |
| 33 | Amikacin | >= 64 | R | Vitek2 |
| 33 | Gentamicin | >= 16 | R | Vitek2 |
| 33 | Ciprofloxacin | >= 4 | R | Vitek2 |
| 33 | Colistin | 12 mm | S | Manual |
| 34 | Piperacillin/Tazobactam | >= 128 | R | Vitek2 |
| 34 | Ceftazidime/Avibactam | 16 mm | R | Manual |
| 34 | Ceftolozane/Tazobactam | 15 mm | R | Manual |
| 34 | Ceftazidime | 16 | R | Vitek2 |
| 34 | Cefepime | 8 | R | Vitek2 |
| 34 | Imipenem | >= 16 | R | Vitek2 |
| 34 | Meropenem | >= 16 | R | Vitek2 |
| 34 | Amikacin | 8 | S | Vitek2 |
| 34 | Gentamicin | 4 | S | Vitek2 |
| 34 | Ciprofloxacin | 2 | R | Vitek2 |
| 34 | Colistin | 2 | S | Vitek2 |
| 35 | Piperacillin/Tazobactam | >= 128 | R | Vitek2 |
| 35 | Ceftazidime/Avibactam | >= 16 | R | Vitek2 |
| 35 | Ceftolozane/Tazobactam | >= 32 | R | Vitek2 |
| 35 | Ceftazidime | >= 64 | R | Vitek2 |
| 35 | Cefepime | >= 32 | R | Vitek2 |
| 35 | Imipenem | 4 | I | Vitek2 |
| 35 | Meropenem | 1 | S | Vitek2 |
| 35 | Amikacin | 4 | S | Vitek2 |
| 35 | Gentamicin | 4 | S | Vitek2 |
| 35 | Ciprofloxacin | 0.5 | S | Vitek2 |
| 35 | Colistin | 2 | S | Vitek2 |
| 36 | Piperacillin/Tazobactam | >= 128 | R | Vitek2 |
| 36 | Ceftazidime/Avibactam | 4 | S | Vitek2 |
| 36 | Ceftolozane/Tazobactam | 2 | S | Vitek2 |
| 36 | Ceftazidime | 2 | R | Vitek2 |
| 36 | Cefepime | 16 | R | Vitek2 |
| 36 | Imipenem | >= 16 | R | Vitek2 |
| 36 | Meropenem | 4 | S | Vitek2 |
| 36 | Amikacin | >= 64 | R | Vitek2 |
| 36 | Gentamicin | >= 16 | R | Vitek2 |
| 36 | Ciprofloxacin | 2 | I | Vitek2 |
| 36 | Colistin | 12 mm | S | Manual |
| 37 | Piperacillin/Tazobactam | >= 128 | R | Vitek2 |
| 37 | Ceftazidime/Avibactam | 1 | S | Vitek2 |
| 37 | Ceftolozane/Tazobactam | 16 | R | Vitek2 |
| 37 | Ceftazidime | >= 64 | R | Vitek2 |
| 37 | Cefepime | >= 32 | R | Vitek2 |
| 37 | Imipenem | 8 | R | Vitek2 |
| 37 | Meropenem | 8 | I | Vitek2 |
| 37 | Amikacin | 2 | S | Vitek2 |
| 37 | Gentamicin | >= 16 | R | Vitek2 |
| 37 | Ciprofloxacin | >= 4 | R | Vitek2 |
| 37 | Colistin | — | — | — |
| 38 | Piperacillin/Tazobactam | >= 128 | R | Vitek2 |
| 38 | Ceftazidime/Avibactam | >= 16 | R | Vitek2 |
| 38 | Ceftolozane/Tazobactam | >= 32 | R | Vitek2 |
| 38 | Ceftazidime | >= 64 | R | Vitek2 |
| 38 | Cefepime | 16 | R | Vitek2 |
| 38 | Imipenem | >= 16 | R | Vitek2 |
| 38 | Meropenem | >= 16 | R | Vitek2 |
| 38 | Amikacin | 4 | S | Vitek2 |
| 38 | Gentamicin | <= 1 | S | Vitek2 |
| 38 | Ciprofloxacin | >= 4 | R | Vitek2 |
| 38 | Colistin | — | — | — |
| 39 | Piperacillin/Tazobactam | 27 mm | S | Manual |
| 39 | Ceftazidime/Avibactam | — | — | — |
| 39 | Ceftolozane/Tazobactam | — | — | — |
| 39 | Ceftazidime | 12 mm | R | Manual |
| 39 | Cefepime | — | — | — |
| 39 | Imipenem | 24 mm | S | Manual |
| 39 | Meropenem | 30 mm | S | Manual |
| 39 | Amikacin | 23 mm | S | Manual |
| 39 | Gentamicin | 20 mm | S | Manual |
| 39 | Ciprofloxacin | 29 mm | S | Manual |
| 39 | Colistin | 12 mm | S | Manual |
| 40 | Piperacillin/Tazobactam | — | — | — |
| 40 | Ceftazidime/Avibactam | — | — | — |
| 40 | Ceftolozane/Tazobactam | — | — | — |
| 40 | Ceftazidime | 12 mm | R | Manual |
| 40 | Cefepime | 11 mm | R | Manual |
| 40 | Imipenem | 22 mm | S | Manual |
| 40 | Meropenem | 25 mm | S | Manual |
| 40 | Amikacin | 22 mm | S | Manual |
| 40 | Gentamicin | 20 mm | S | Manual |
| 40 | Ciprofloxacin | 30 mm | S | Manual |
| 40 | Colistin | 10 mm | S | Manual |
| 41 | Piperacillin/Tazobactam | 26 mm | S | Manual |
| 41 | Ceftazidime/Avibactam | — | — | — |
| 41 | Ceftolozane/Tazobactam | — | — | — |
| 41 | Ceftazidime | 15 mm | I | Manual |
| 41 | Cefepime | 25 mm | S | Manual |
| 41 | Imipenem | 33 mm | S | Manual |
| 41 | Meropenem | 40 mm | S | Manual |
| 41 | Amikacin | 22 mm | S | Manual |
| 41 | Gentamicin | 22 mm | S | Manual |
| 41 | Ciprofloxacin | 30 mm | S | Manual |
| 41 | Colistin | 11 mm | S | Manual |
| 42 | Piperacillin/Tazobactam | 27 mm | S | Manual |
| 42 | Ceftazidime/Avibactam | — | — | — |
| 42 | Ceftolozane/Tazobactam | — | — | — |
| 42 | Ceftazidime | 24 mm | S | Manual |
| 42 | Cefepime | 22 mm | S | Manual |
| 42 | Imipenem | 23 mm | S | Manual |
| 42 | Meropenem | 30 mm | S | Manual |
| 42 | Amikacin | 25 mm | S | Manual |
| 42 | Gentamicin | 23 mm | S | Manual |
| 42 | Ciprofloxacin | 27 mm | S | Manual |
| 42 | Colistin | 10 mm | S | Manual |
| 43 | Piperacillin/Tazobactam | 23 mm | S | Manual |
| 43 | Ceftazidime/Avibactam | — | — | — |
| 43 | Ceftolozane/Tazobactam | — | — | — |
| 43 | Ceftazidime | 20 mm | S | Manual |
| 43 | Cefepime | 24 mm | S | Manual |
| 43 | Imipenem | 25 mm | S | Manual |
| 43 | Meropenem | 30 mm | S | Manual |
| 43 | Amikacin | — | — | — |
| 43 | Gentamicin | — | — | — |
| 43 | Ciprofloxacin | — | — | — |
| 43 | Colistin | 10 mm | S | Manual |
| 44 | Piperacillin/Tazobactam | 12 mm | R | Manual |
| 44 | Ceftazidime/Avibactam | — | — | — |
| 44 | Ceftolozane/Tazobactam | — | — | — |
| 44 | Ceftazidime | 12 mm | R | Manual |
| 44 | Cefepime | — | — | — |
| 44 | Imipenem | 25 mm | S | Manual |
| 44 | Meropenem | 29 mm | S | Manual |
| 44 | Amikacin | — | — | — |
| 44 | Gentamicin | — | — | — |
| 44 | Ciprofloxacin | 28 mm | S | Manual |
| 44 | Colistin | 12 mm | S | Manual |
| 45 | Piperacillin/Tazobactam | 32 mm | S | Manual |
| 45 | Ceftazidime/Avibactam | — | — | — |
| 45 | Ceftolozane/Tazobactam | — | — | — |
| 45 | Ceftazidime | 22 mm | S | Manual |
| 45 | Cefepime | 33 mm | S | Manual |
| 45 | Imipenem | 30 mm | S | Manual |
| 45 | Meropenem | 22 mm | S | Manual |
| 45 | Amikacin | 30 mm | S | Manual |
| 45 | Gentamicin | 35 mm | S | Manual |
| 45 | Ciprofloxacin | 34 mm | S | Manual |
| 45 | Colistin | 18 mm | S | Manual |
| 46 | Piperacillin/Tazobactam | 8 | S | Vitek2 |
| 46 | Ceftazidime/Avibactam | — | — | — |
| 46 | Ceftolozane/Tazobactam | — | — | — |
| 46 | Ceftazidime | 2 | S | Vitek2 |
| 46 | Cefepime | 2 | S | Vitek2 |
| 46 | Imipenem | 2 | S | Vitek2 |
| 46 | Meropenem | <= 0.25 | S | Vitek2 |
| 46 | Amikacin | 4 | S | Vitek2 |
| 46 | Gentamicin | 2 | S | Vitek2 |
| 46 | Ciprofloxacin | 0.25 | S | Vitek2 |
| 46 | Colistin | 2 | S | Vitek2 |
| 47 | Piperacillin/Tazobactam | 23 mm | S | Manual |
| 47 | Ceftazidime/Avibactam | — | — | — |
| 47 | Ceftolozane/Tazobactam | — | — | — |
| 47 | Ceftazidime | 19 mm | S | Manual |
| 47 | Cefepime | 26 mm | S | Manual |
| 47 | Imipenem | 26 mm | S | Manual |
| 47 | Meropenem | 30 mm | S | Manual |
| 47 | Amikacin | 23 mm | S | Manual |
| 47 | Gentamicin | 20 mm | S | Manual |
| 47 | Ciprofloxacin | 24 mm | S | Manual |
| 47 | Colistin | 13 mm | S | Manual |
| 48 | Piperacillin/Tazobactam | 23 mm | S | Manual |
| 48 | Ceftazidime/Avibactam | — | — | — |
| 48 | Ceftolozane/Tazobactam | — | — | — |
| 48 | Ceftazidime | 21 mm | S | Manual |
| 48 | Cefepime | — | — | — |
| 48 | Imipenem | — | — | — |
| 48 | Meropenem | 26 mm | S | Manual |
| 48 | Amikacin | 27 mm | S | Manual |
| 48 | Gentamicin | 21 mm | S | Manual |
| 48 | Ciprofloxacin | 28 mm | S | Manual |
| 48 | Colistin | — | — | — |
| 49 | Piperacillin/Tazobactam | 16 | S | Vitek2 |
| 49 | Ceftazidime/Avibactam | — | — | — |
| 49 | Ceftolozane/Tazobactam | — | — | — |
| 49 | Ceftazidime | 2 | S | Vitek2 |
| 49 | Cefepime | 2 | S | Vitek2 |
| 49 | Imipenem | 4 | I | Vitek2 |
| 49 | Meropenem | 1 | S | Vitek2 |
| 49 | Amikacin | 2 | S | Vitek2 |
| 49 | Gentamicin | <= 1 | S | Vitek2 |
| 49 | Ciprofloxacin | 0.25 | S | Vitek2 |
| 49 | Colistin | — | — | — |
| 50 | Piperacillin/Tazobactam | >= 128 | R | Vitek2 |
| 50 | Ceftazidime/Avibactam | >= 16 | R | Vitek2 |
| 50 | Ceftolozane/Tazobactam | >= 32 | R | Vitek2 |
| 50 | Ceftazidime | >= 64 | R | Vitek2 |
| 50 | Cefepime | >= 32 | R | Vitek2 |
| 50 | Imipenem | 8 | R | Vitek2 |
| 50 | Meropenem | 8 | I | Vitek2 |
| 50 | Amikacin | 4 | S | Vitek2 |
| 50 | Gentamicin | >= 16 | R | Vitek2 |
| 50 | Ciprofloxacin | >= 4 | R | Vitek2 |
| 50 | Colistin | >= 16 | R | Vitek2 |
