# Supplementary material for: Molecular detection of blaVIM and blaNDM in multidrug-resistant Pseudomonas aeruginosa from cancer and burn patients in Erbil, Iraq
Source: Front Microbiol. 2025 Sep 15;16:1672531. doi: 10.3389/fmicb.2025.1672531 (PMC12477123; doi:10.3389/fmicb.2025.1672531)
Supplement: Supplementary file 1 [file Data_Sheet_1.zip › latest_supplementary_material file/Supplementary_Tables/Supplementary_Table_S5.docx]

**Supplementary Table 5.** Significant pairwise differences in resistance (Tukey’s test).

| **Comparison** | **Mean Diff** | **95% CI** | **Summary** | **Adjusted *P* Value** |
| --- | --- | --- | --- | --- |
| **Ceftazidime: Burn vs Colistin: Other** | 119.1 | 69.02 to 169.2 | **** | <0.0001 |
| **Cefepime: Burn vs Colistin: Other** | 115.8 | 65.68 to 165.9 | **** | <0.0001 |
| **Piperacillin/Tazobactam:Burn vs Colistin:Other** | 114.1 | 64.02 to 164.2 | **** | <0.0001 |
| **Ceftolozane/Tazobactam:Burn vs Colistin:Other** | 112.5 | 62.35 to 162.6 | **** | <0.0001 |
| **Ceftazidime/Avibactam: Burn vs Colistin: Other** | 110.8 | 60.68 to 160.9 | **** | <0.0001 |
| **Ceftazidime: Burn vs Colistin: Cancer** | 106.5 | 56.42 to 156.6 | **** | <0.0001 |
| **Imipenem: Burn vs Colistin: Other** | 105.3 | 55.21 to 155.4 | **** | <0.0001 |
| **Ciprofloxacin: Burn vs Colistin: Other** | 103.6 | 53.54 to 153.7 | **** | <0.0001 |
| **Cefepime: Burn vs Colistin: Cancer** | 103.2 | 53.08 to 153.3 | **** | <0.0001 |
| **Gentamicin: Burn vs Colistin: Other** | 102 | 51.87 to 152.1 | **** | <0.0001 |
| **Piperacillin/Tazobactam:Burn vs Colistin:Cancer** | 101.5 | 51.42 to 151.6 | **** | <0.0001 |
| **Meropenem: Burn vs Colistin: Other** | 100.3 | 50.21 to 150.4 | **** | <0.0001 |
| **Ceftolozane/Tazobactam:Burn vs Colistin:Cancer** | 99.85 | 49.75 to 150.0 | **** | <0.0001 |
| **Ceftazidime/Avibactam: Burn vs Colistin: Cancer** | 98.19 | 48.08 to 148.3 | **** | <0.0001 |
| **Amikacin: Burn vs Colistin: Other** | 96.26 | 46.16 to 146.4 | **** | <0.0001 |
| **Imipenem: Burn vs Colistin: Cancer** | 92.71 | 42.61 to 142.8 | **** | <0.0001 |
| **Ciprofloxacin: Burn vs Colistin: Cancer** | 91.04 | 40.94 to 141.1 | **** | <0.0001 |
| **Gentamicin: Burn vs Colistin: Cancer** | 89.38 | 39.27 to 139.5 | **** | <0.0001 |
| **Meropenem: Burn vs Colistin: Cancer** | 87.71 | 37.61 to 137.8 | **** | <0.0001 |

*Full comparison results are in Supplementary Table S6. **** P < 0.0001.*
