# Supplementary material for: Molecular detection of blaVIM and blaNDM in multidrug-resistant Pseudomonas aeruginosa from cancer and burn patients in Erbil, Iraq
Source: Front Microbiol. 2025 Sep 15;16:1672531. doi: 10.3389/fmicb.2025.1672531 (PMC12477123; doi:10.3389/fmicb.2025.1672531)
Supplement: Supplementary file 1 [file Data_Sheet_1.zip › latest_supplementary_material file/Supplementary_Tables/Supplementary_Table_S7.docx]

**Supplementary** **Table 7.** Distribution of *bla_VIM_* and *bla_NDM_* genes across AES phenotypes.

| **AES β-lactams Phenotype** | **N** | ***bla_VIM_*-positive (%)** | ***bla_NDM_*-positive (%)** |
| --- | --- | --- | --- |
| **Carbapenemase** | 26 | 24 (92.3%) | 13 (50.0%) |
| **ESBL** | 31 | 24 (77.4%) | 15 (48.4%) |
| **Acquired Penicillinase** | 7 | 5 (71.4%) | 0 (0.0%) |
| **ESBL (Clavulanate Inhibited)** | 4 | 3 (75.0%) | 1 (25.0%) |
| **HL CASE + R Carbapenems** | 28 | 22 (78.6%) | 15 (53.6%) |
| **IMPER** | 28 | 22 (78.6%) | 15 (53.6%) |
| **Wild** | 7 | 5 (71.4%) | 0 (0.0%) |
| **Overall *p*-value (Fisher's exact)** |  | **0.601 (ns)** | **0.0169 (*)** |

*Statistical significance: ns = not significant, *p < 0.05, **p < 0.01, ***p < 0.001*.
