# Supplementary material for: Molecular detection of blaVIM and blaNDM in multidrug-resistant Pseudomonas aeruginosa from cancer and burn patients in Erbil, Iraq
Source: Front Microbiol. 2025 Sep 15;16:1672531. doi: 10.3389/fmicb.2025.1672531 (PMC12477123; doi:10.3389/fmicb.2025.1672531)
Supplement: Supplementary file 1 [file Data_Sheet_1.zip › latest_supplementary_material file/Supplementary_Tables/Supplementary_Table_S8.docx]

**Supplementary** **Table 8.** Association between ***bla_VIM_*** and ***bla_NDM_*** gene carriage and carbapenem resistance (n = 40).

| **Resistance Gene** | **Carbapenem Resistant (%)** | **Carbapenem Sensitive (%)** | **Fisher's exact *p*-value** |
| --- | --- | --- | --- |
| ***bla_VIM_*-positive (n=31)** | 23 (74.2%) | 8 (25.8%) | 0.686 (ns) |
| ***bla_VIM_*-negative (n=9)** | 6 (66.7%) | 3 (33.3%) |  |
| ***bla_NDM_*-positive (n=16)** | 15 (93.8%) | 1 (6.3%) | 0.027* |
| ***bla_NDM_*-negative (n=24)** | 14 (58.3%) | 10 (41.7%) |  |

*Statistical significance: ns = not significant at p < 0.05; * = significant at p < 0.05.*
