# Supplementary material for: Molecular detection of blaVIM and blaNDM in multidrug-resistant Pseudomonas aeruginosa from cancer and burn patients in Erbil, Iraq
Source: Front Microbiol. 2025 Sep 15;16:1672531. doi: 10.3389/fmicb.2025.1672531 (PMC12477123; doi:10.3389/fmicb.2025.1672531)
Supplement: Supplementary file 1 [file Data_Sheet_1.zip › latest_supplementary_material file/Supplementary_Tables/Supplementary_Table_S9.docx]

**Supplementary Table 9.** Co-occurrence of *bla_VIM_* and *bla_NDM_* genes among *Pseudomonas aeruginosa* isolates (n = 40).

| **Gene Carriage** | ***bla_NDM_*-positive (%)** | ***bla_NDM_*-negative (%)** | **Fisher's exact *p*-value** |
| --- | --- | --- | --- |
| ***bla_VIM_*-positive (n=31)** | 12 (38.7%) | 19 (61.3%) | >0.9999 (ns) |
| ***bla_VIM_*-negative (n=9)** | 4 (44.4%) | 5 (55.6%) |  |

*Statistical significance: ns = not significant at p < 0.05.*
