# Supplementary figures and images for: Molecular detection of blaVIM and blaNDM in multidrug-resistant Pseudomonas aeruginosa from cancer and burn patients in Erbil, Iraq
Source: Front Microbiol. 2025 Sep 15;16:1672531. doi: 10.3389/fmicb.2025.1672531 (PMC12477123; doi:10.3389/fmicb.2025.1672531)

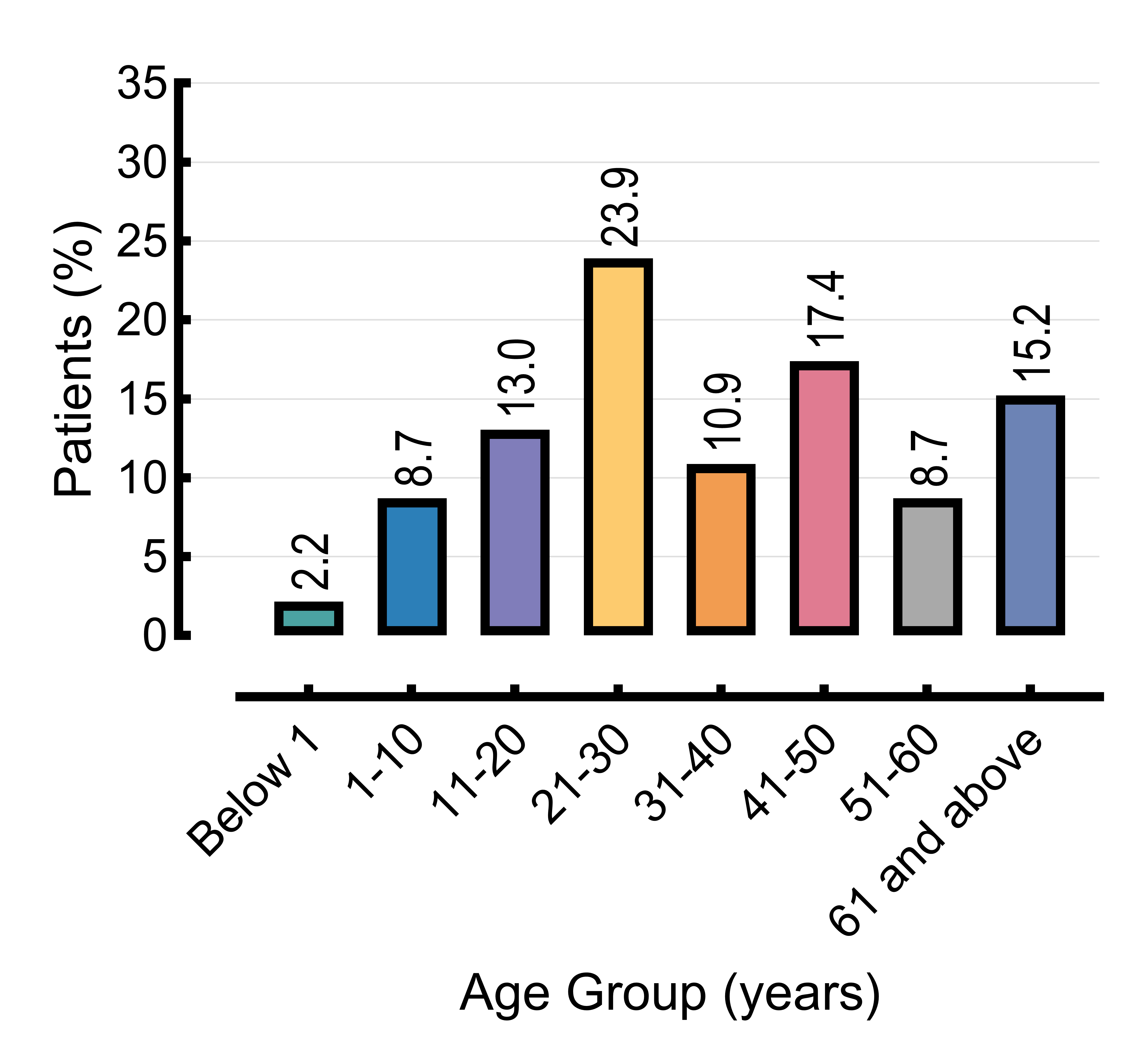

Supplement: Supplementary file 1 [file Data_Sheet_1.zip › latest_supplementary_material file/Supplementary_Figures_TIFF/Supplementary_Figure_S1.tiff]

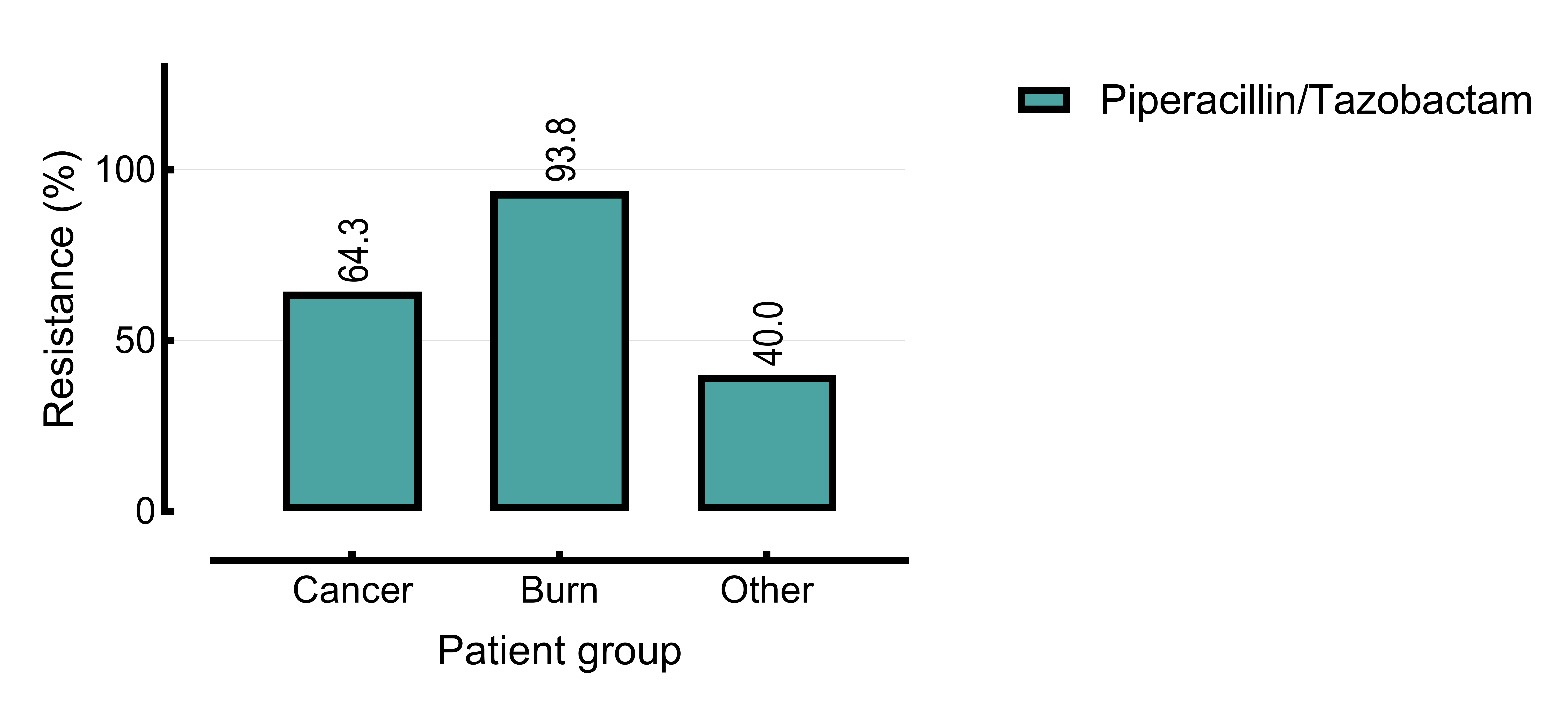

Supplement: Supplementary file 1 [file Data_Sheet_1.zip › latest_supplementary_material file/Supplementary_Figures_TIFF/Supplementary_Figure_S10.tiff]

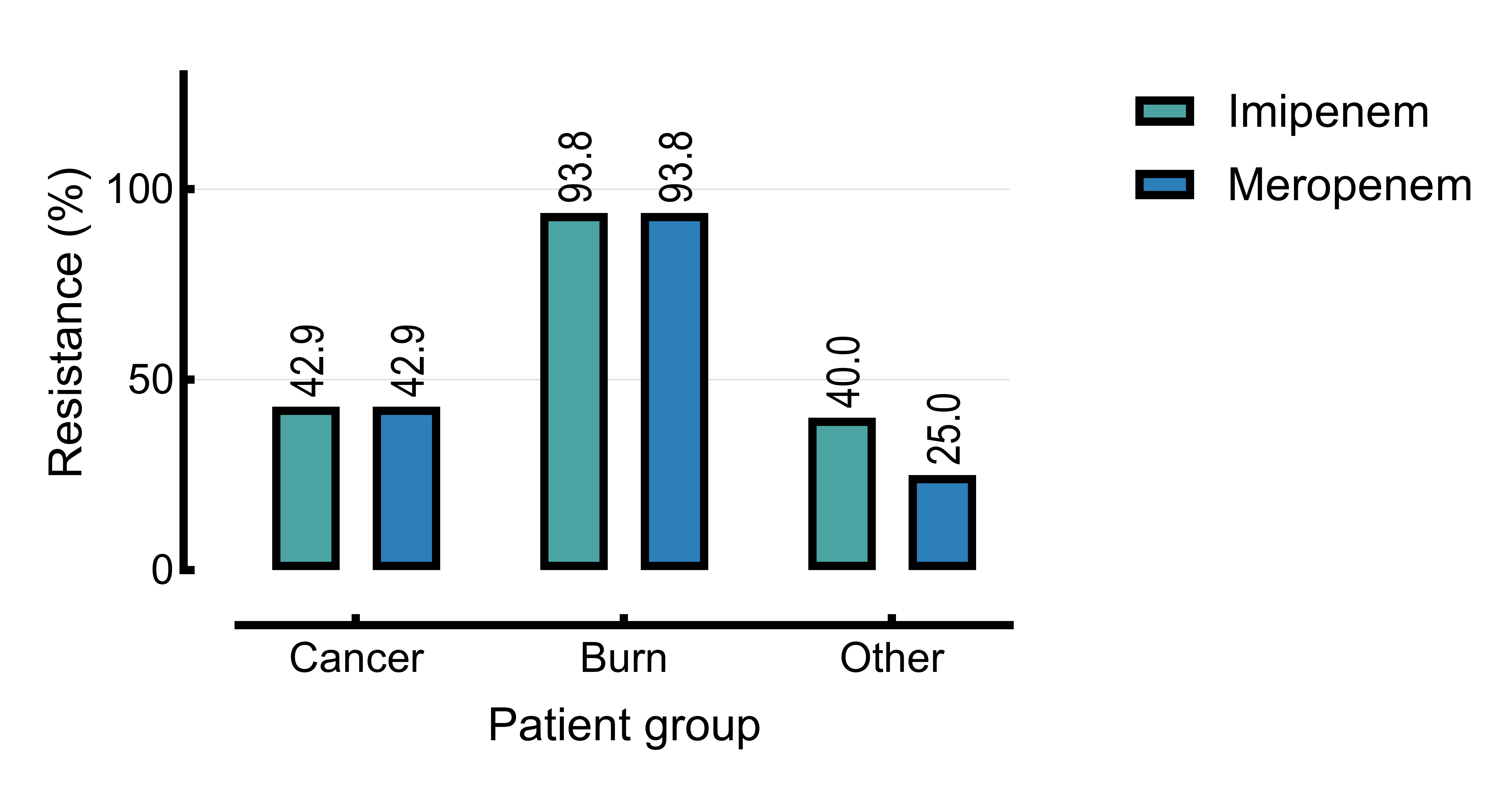

Supplement: Supplementary file 1 [file Data_Sheet_1.zip › latest_supplementary_material file/Supplementary_Figures_TIFF/Supplementary_Figure_S11.tiff]

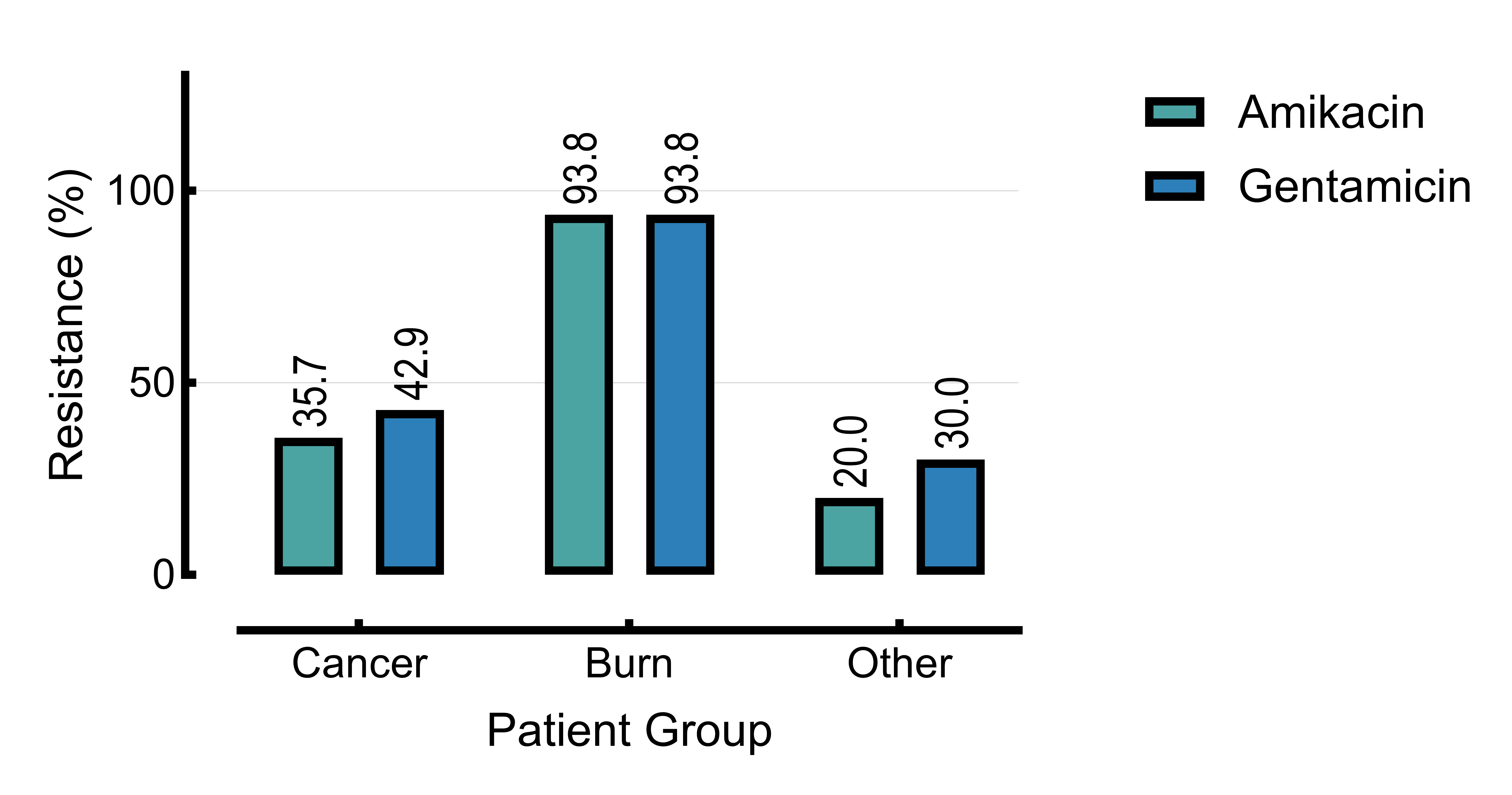

Supplement: Supplementary file 1 [file Data_Sheet_1.zip › latest_supplementary_material file/Supplementary_Figures_TIFF/Supplementary_Figure_S12.tiff]

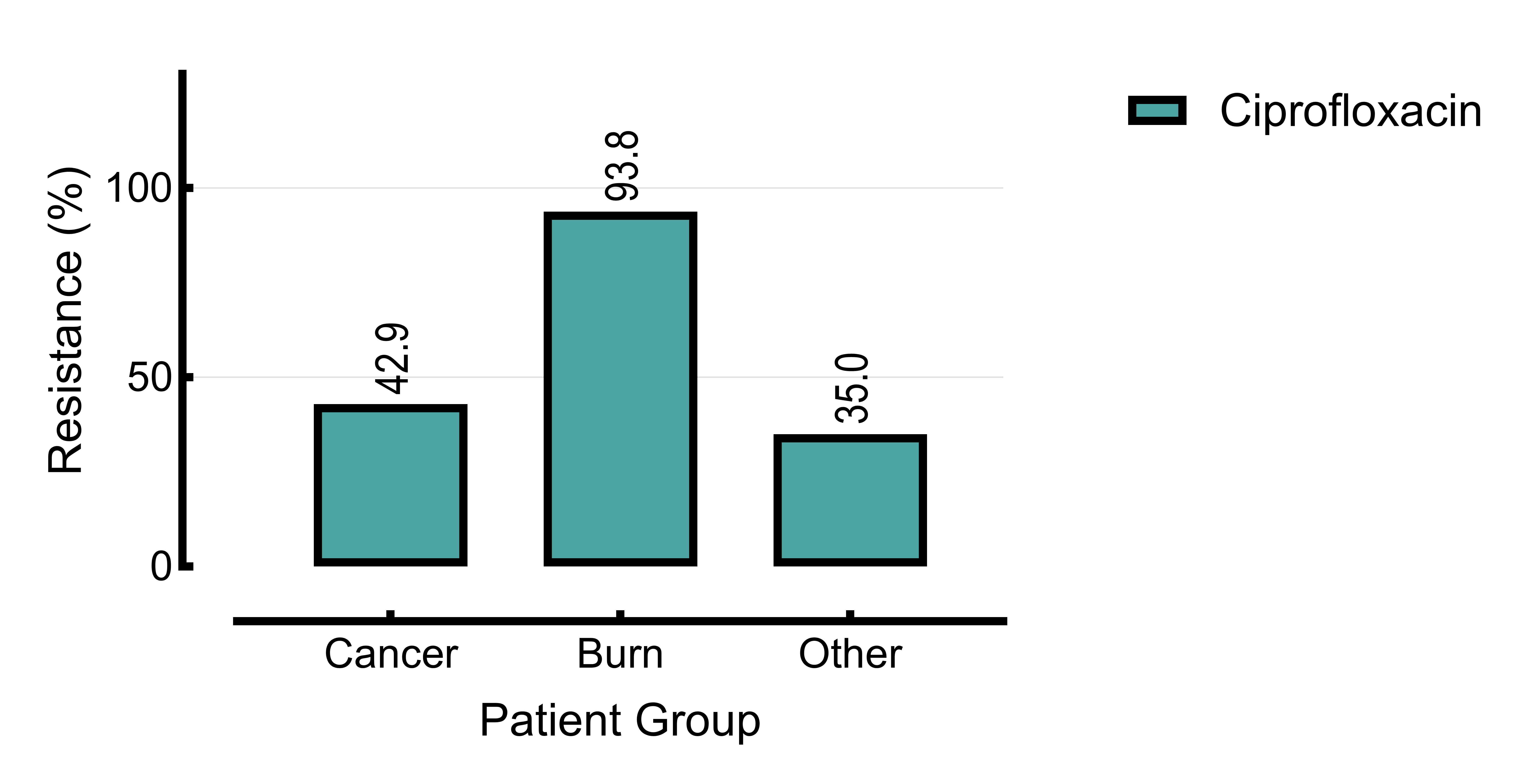

Supplement: Supplementary file 1 [file Data_Sheet_1.zip › latest_supplementary_material file/Supplementary_Figures_TIFF/Supplementary_Figure_S13.tiff]

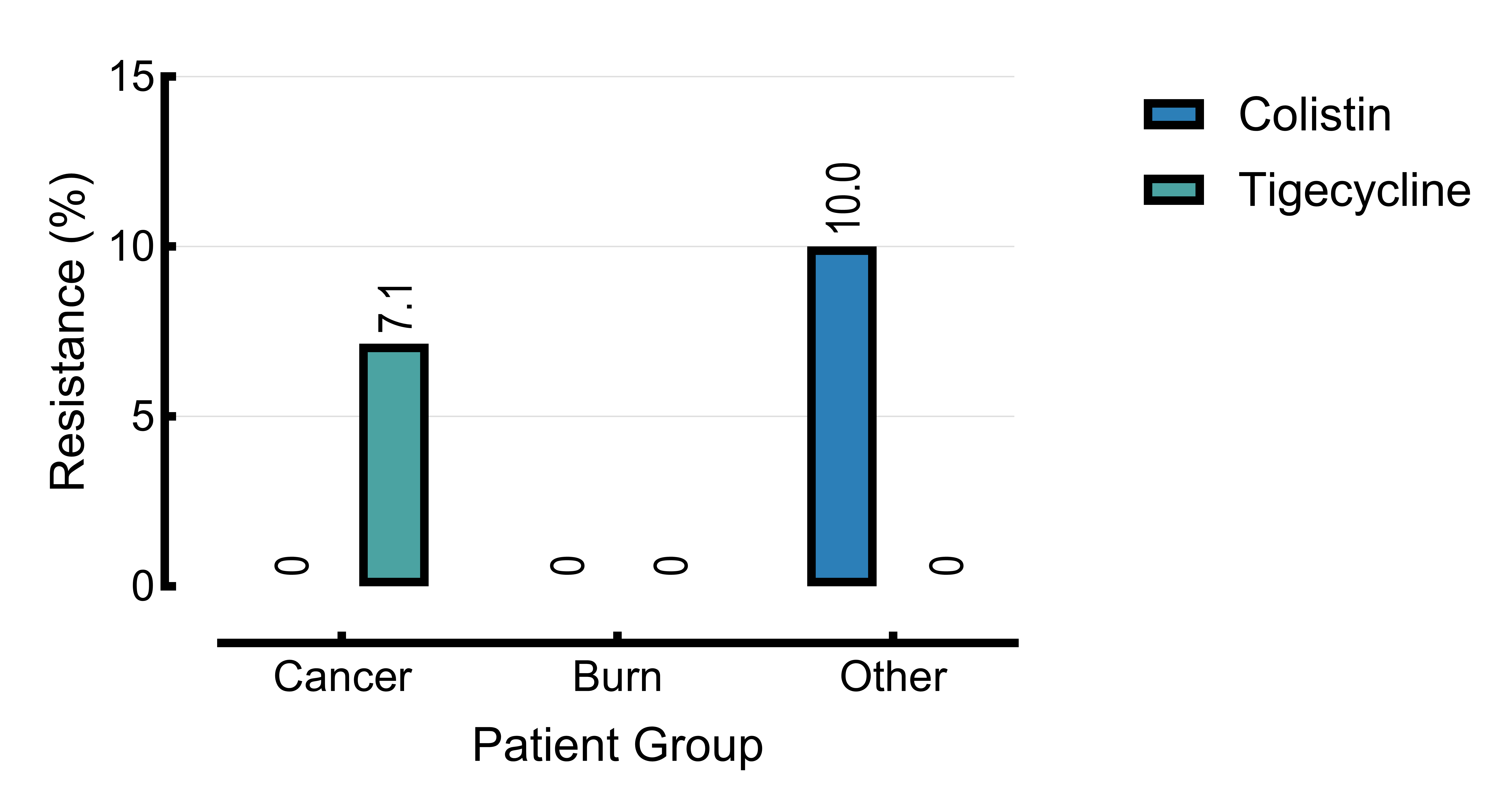

Supplement: Supplementary file 1 [file Data_Sheet_1.zip › latest_supplementary_material file/Supplementary_Figures_TIFF/Supplementary_Figure_S14.tiff]

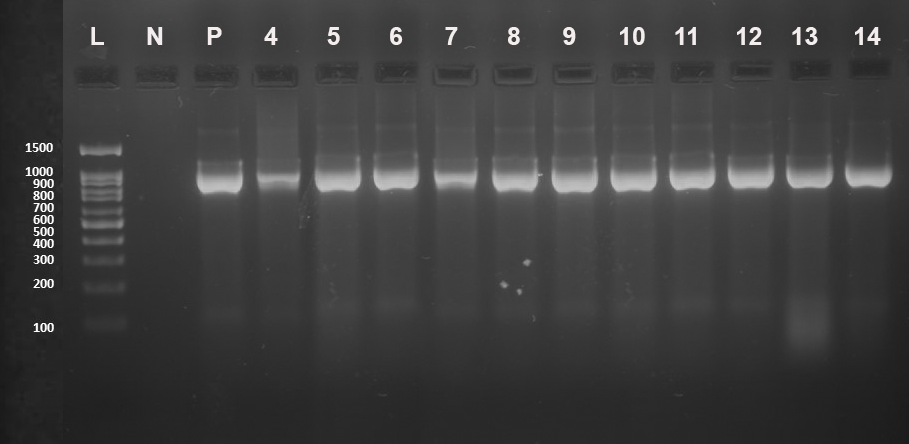

Supplement: Supplementary file 1 [file Data_Sheet_1.zip › latest_supplementary_material file/Supplementary_Figures_TIFF/Supplementary_Figure_S15.tiff]

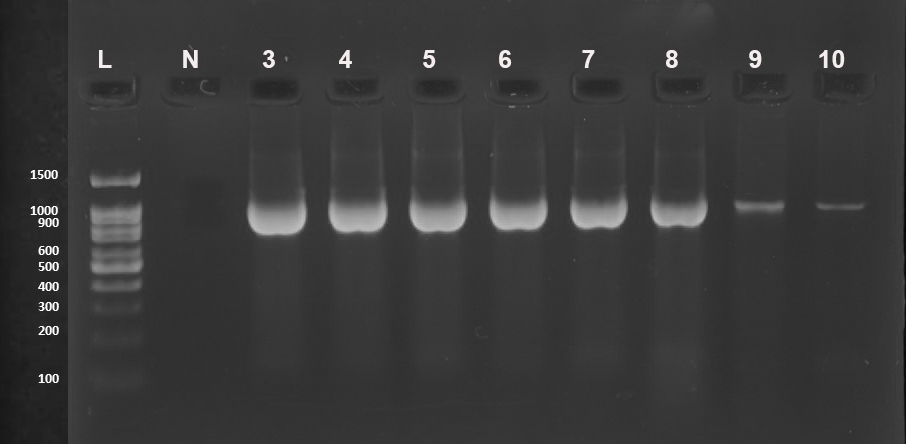

Supplement: Supplementary file 1 [file Data_Sheet_1.zip › latest_supplementary_material file/Supplementary_Figures_TIFF/Supplementary_Figure_S16.tiff]

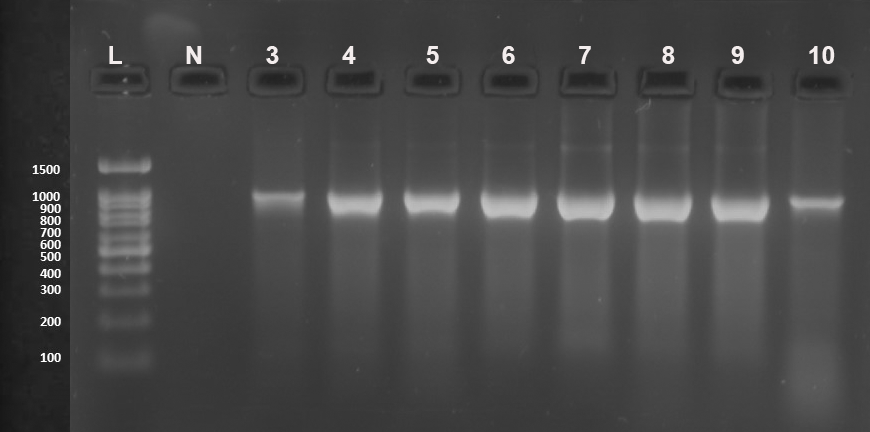

Supplement: Supplementary file 1 [file Data_Sheet_1.zip › latest_supplementary_material file/Supplementary_Figures_TIFF/Supplementary_Figure_S17.tiff]

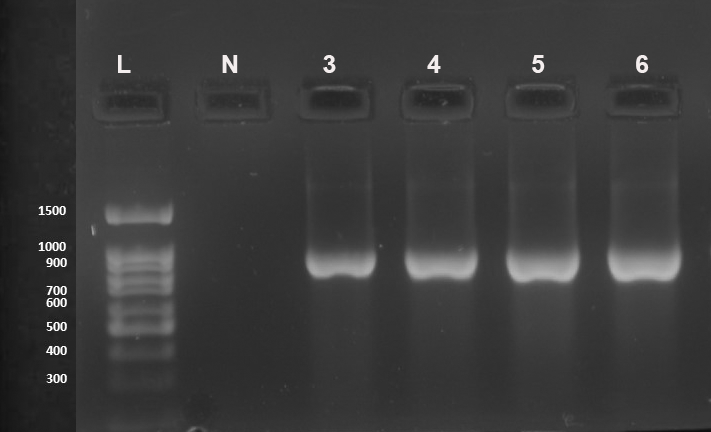

Supplement: Supplementary file 1 [file Data_Sheet_1.zip › latest_supplementary_material file/Supplementary_Figures_TIFF/Supplementary_Figure_S18.tiff]

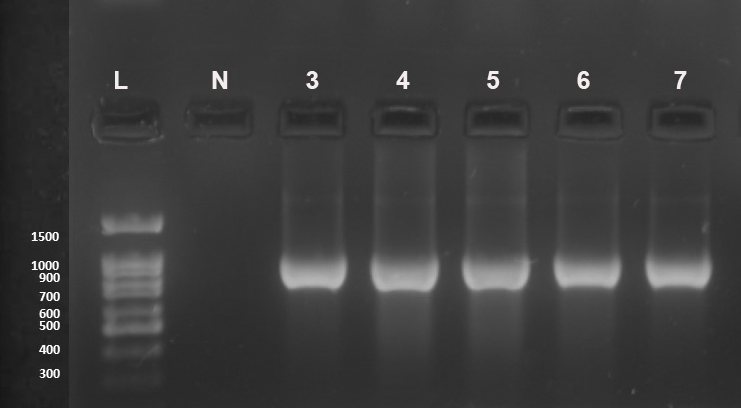

Supplement: Supplementary file 1 [file Data_Sheet_1.zip › latest_supplementary_material file/Supplementary_Figures_TIFF/Supplementary_Figure_S19.tiff]

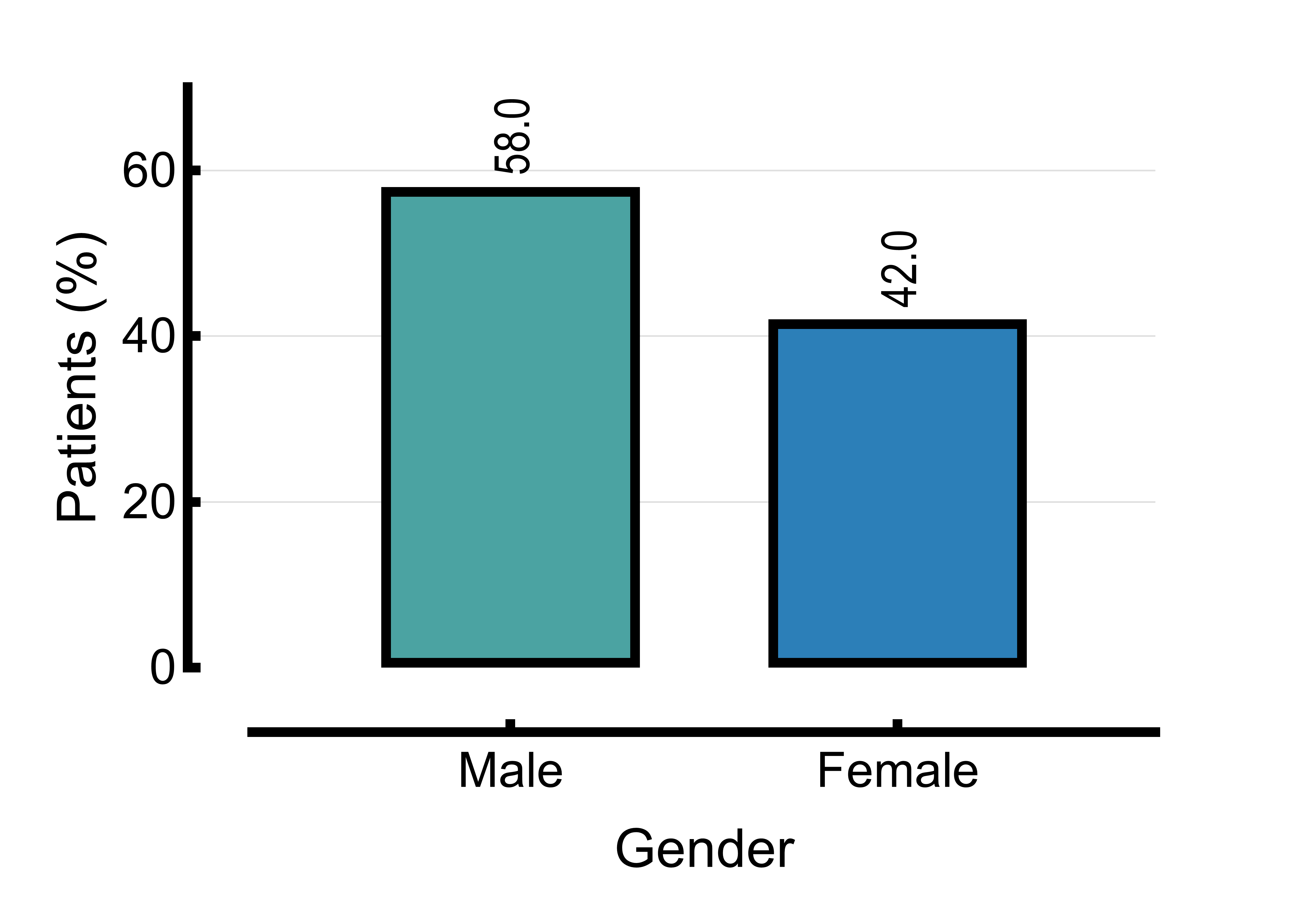

Supplement: Supplementary file 1 [file Data_Sheet_1.zip › latest_supplementary_material file/Supplementary_Figures_TIFF/Supplementary_Figure_S2.tiff]

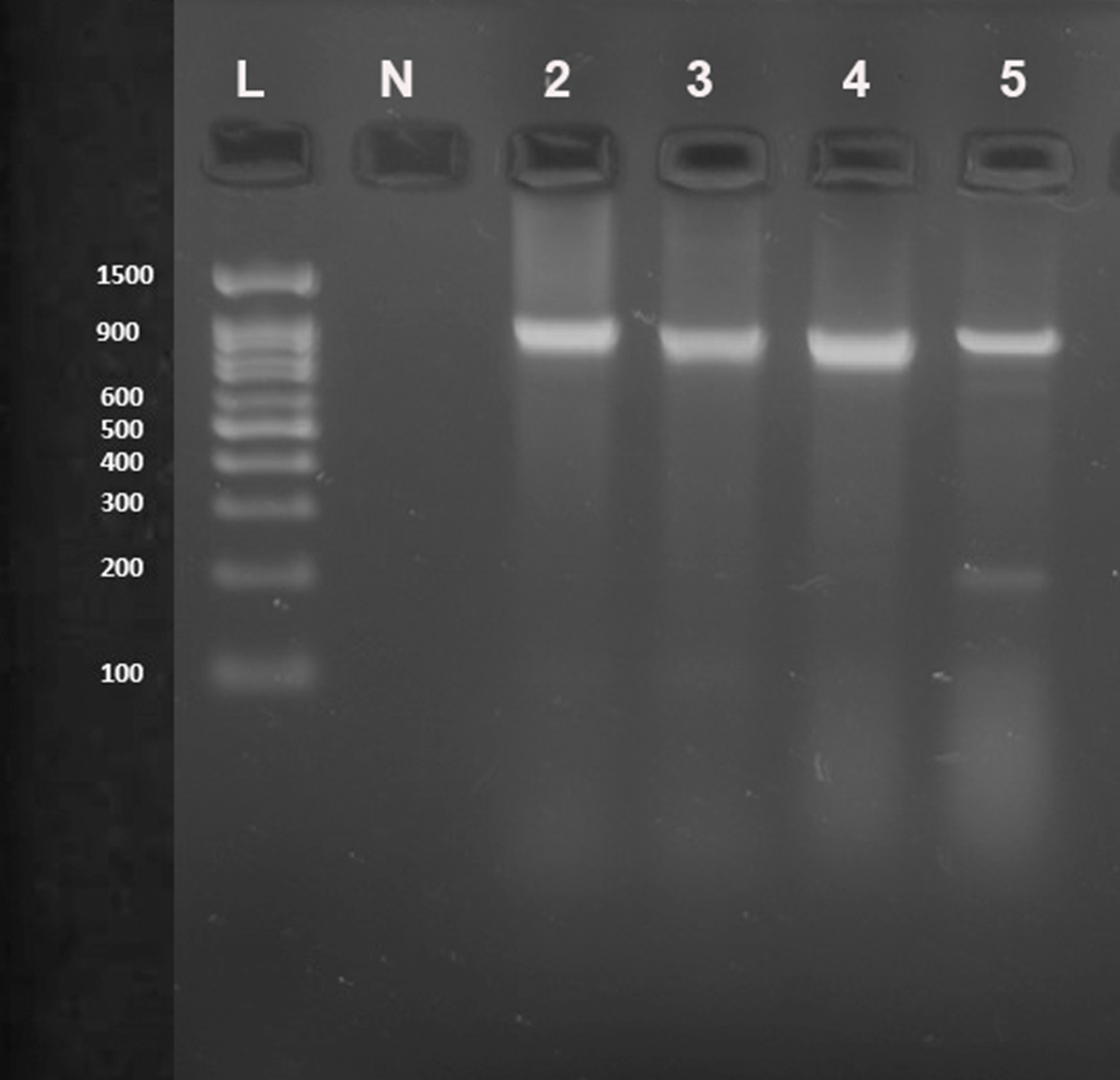

Supplement: Supplementary file 1 [file Data_Sheet_1.zip › latest_supplementary_material file/Supplementary_Figures_TIFF/Supplementary_Figure_S20.tiff]

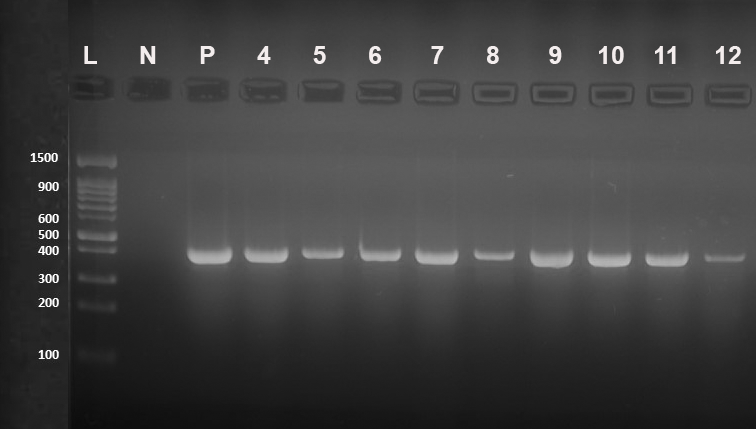

Supplement: Supplementary file 1 [file Data_Sheet_1.zip › latest_supplementary_material file/Supplementary_Figures_TIFF/Supplementary_Figure_S21.tiff]

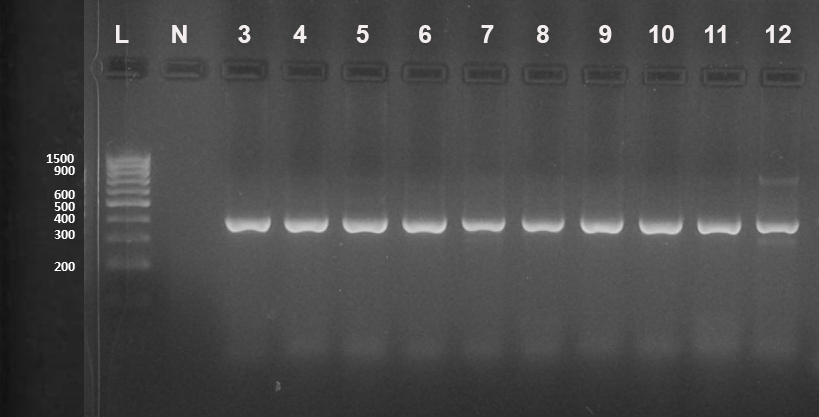

Supplement: Supplementary file 1 [file Data_Sheet_1.zip › latest_supplementary_material file/Supplementary_Figures_TIFF/Supplementary_Figure_S22.tiff]

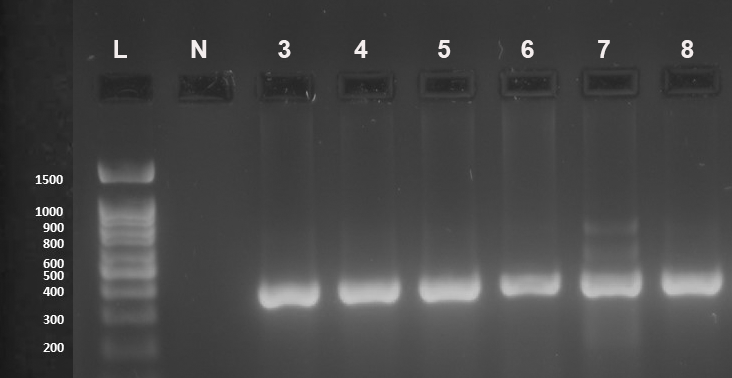

Supplement: Supplementary file 1 [file Data_Sheet_1.zip › latest_supplementary_material file/Supplementary_Figures_TIFF/Supplementary_Figure_S23.tiff]

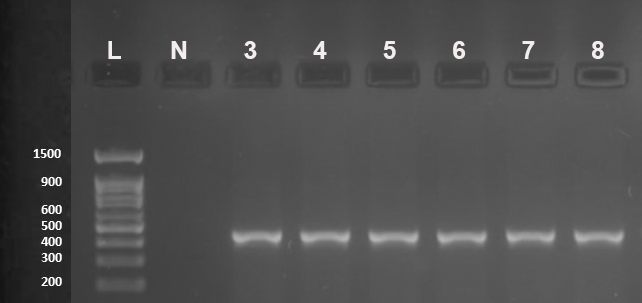

Supplement: Supplementary file 1 [file Data_Sheet_1.zip › latest_supplementary_material file/Supplementary_Figures_TIFF/Supplementary_Figure_S24.tiff]

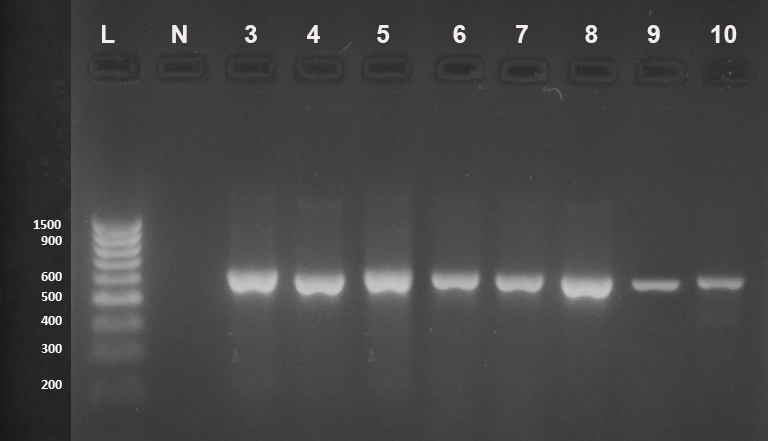

Supplement: Supplementary file 1 [file Data_Sheet_1.zip › latest_supplementary_material file/Supplementary_Figures_TIFF/Supplementary_Figure_S25.tiff]

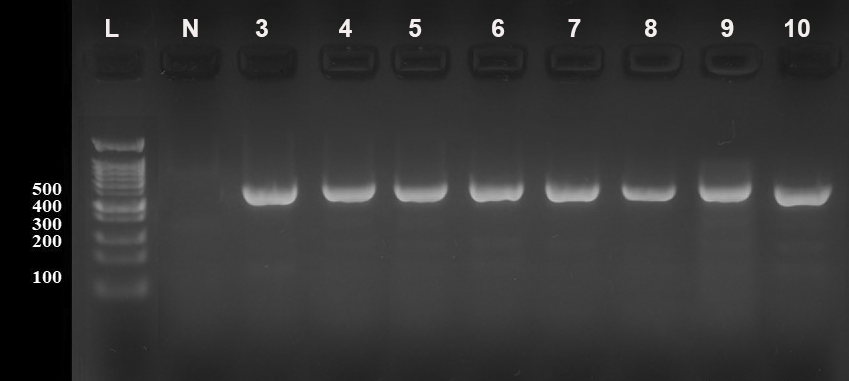

Supplement: Supplementary file 1 [file Data_Sheet_1.zip › latest_supplementary_material file/Supplementary_Figures_TIFF/Supplementary_Figure_S26.tiff]

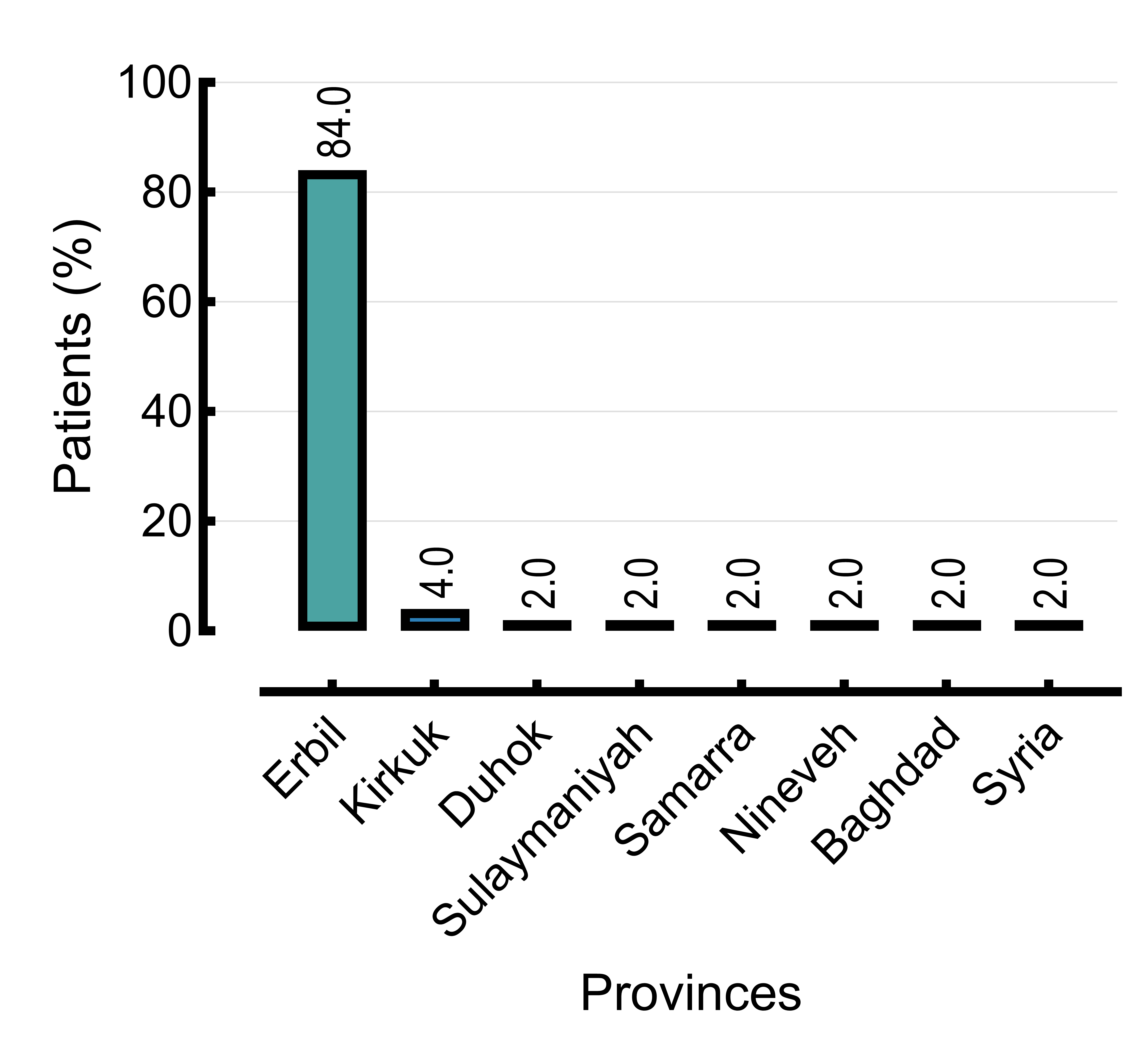

Supplement: Supplementary file 1 [file Data_Sheet_1.zip › latest_supplementary_material file/Supplementary_Figures_TIFF/Supplementary_Figure_S3.tiff]

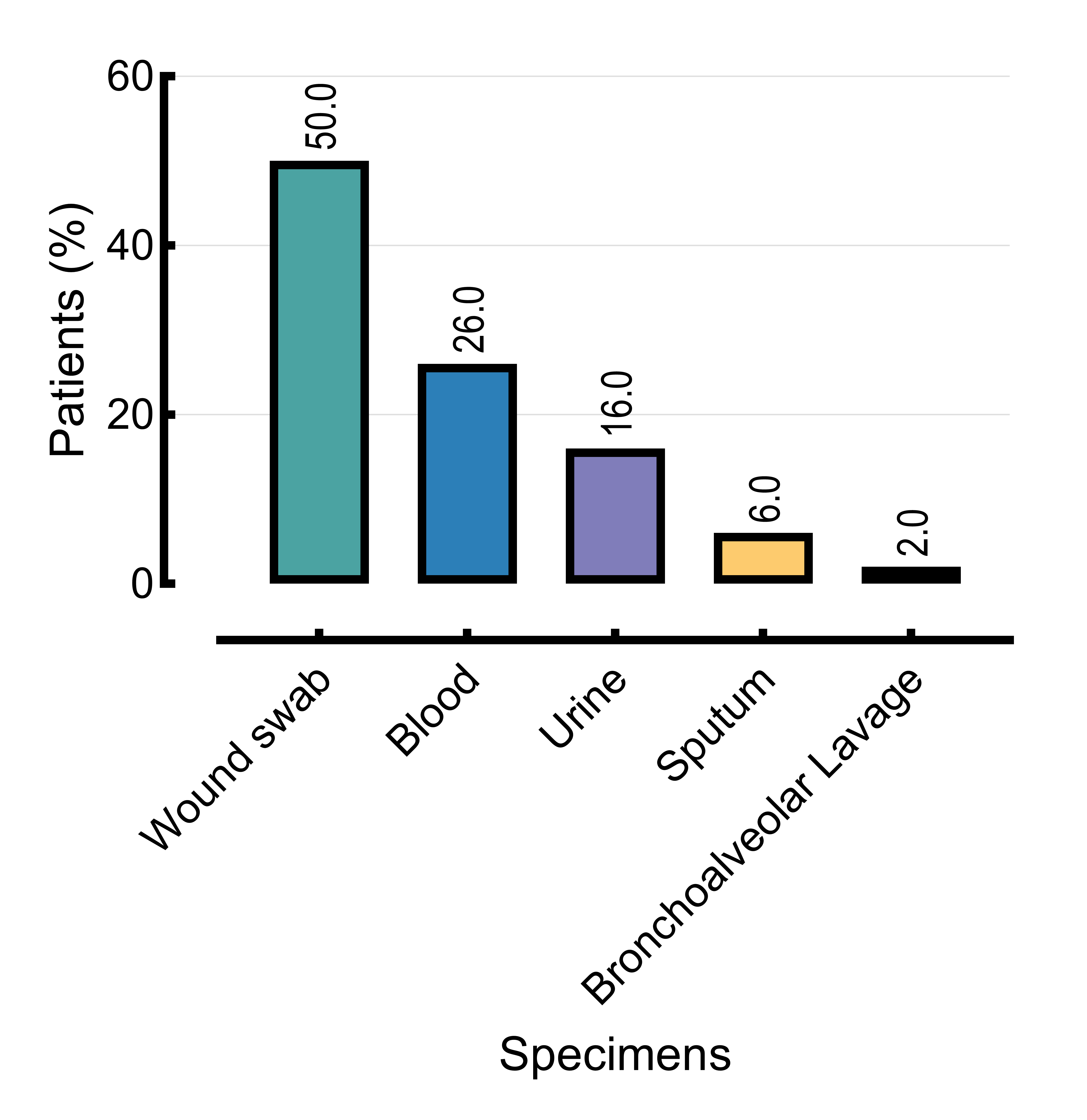

Supplement: Supplementary file 1 [file Data_Sheet_1.zip › latest_supplementary_material file/Supplementary_Figures_TIFF/Supplementary_Figure_S4.tiff]

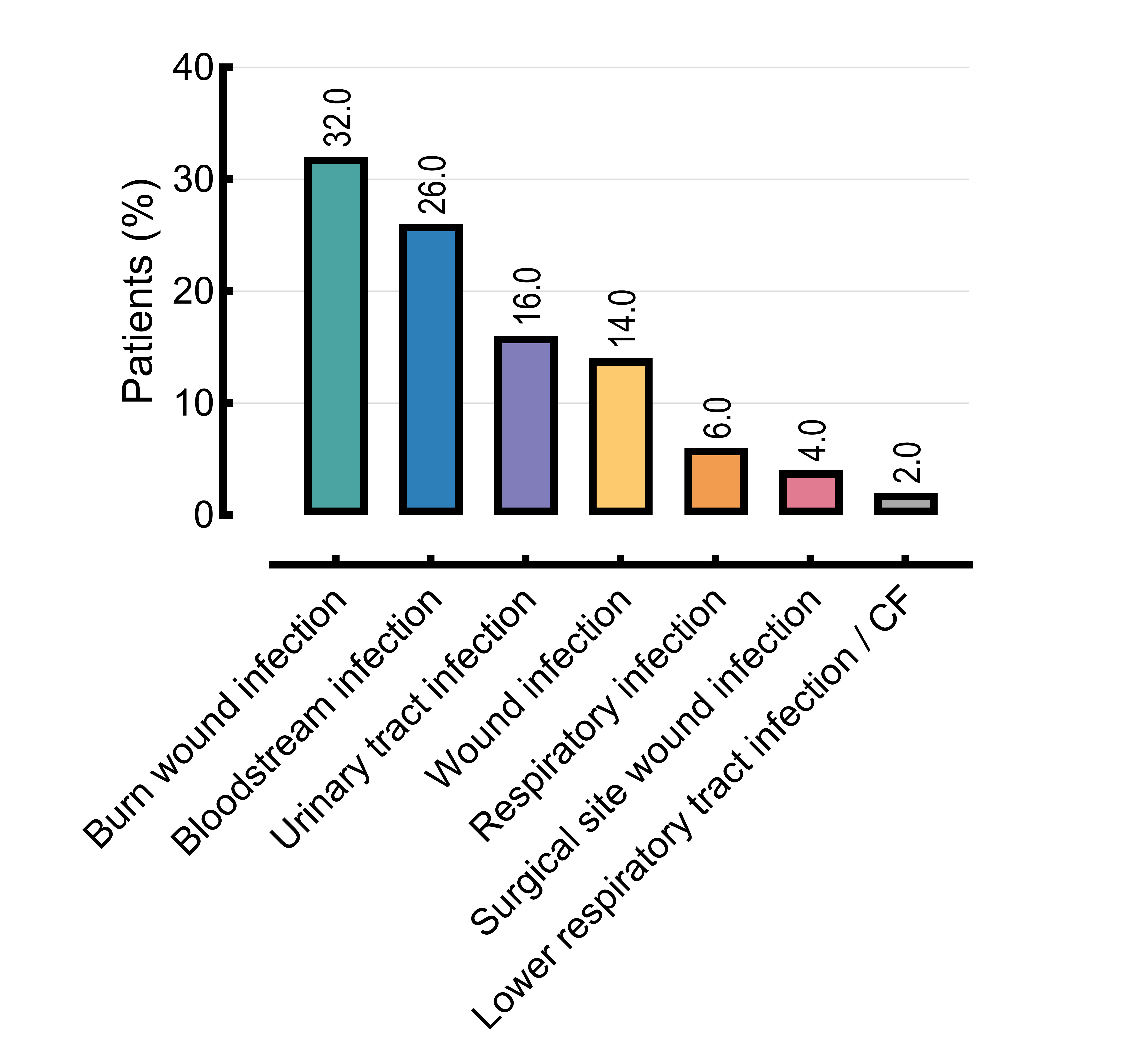

Supplement: Supplementary file 1 [file Data_Sheet_1.zip › latest_supplementary_material file/Supplementary_Figures_TIFF/Supplementary_Figure_S5.tiff]

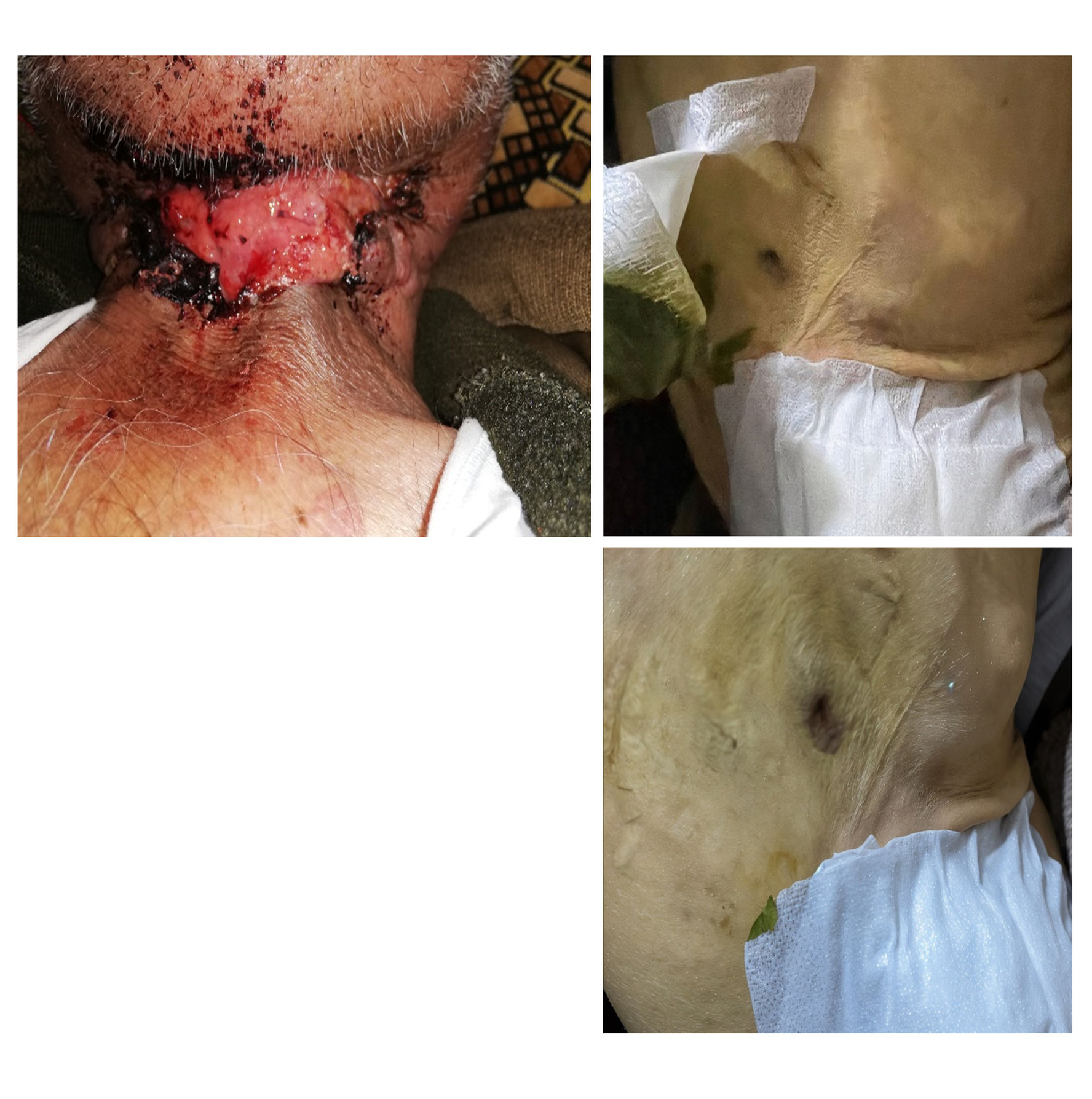

Supplement: Supplementary file 1 [file Data_Sheet_1.zip › latest_supplementary_material file/Supplementary_Figures_TIFF/Supplementary_Figure_S6.tiff]

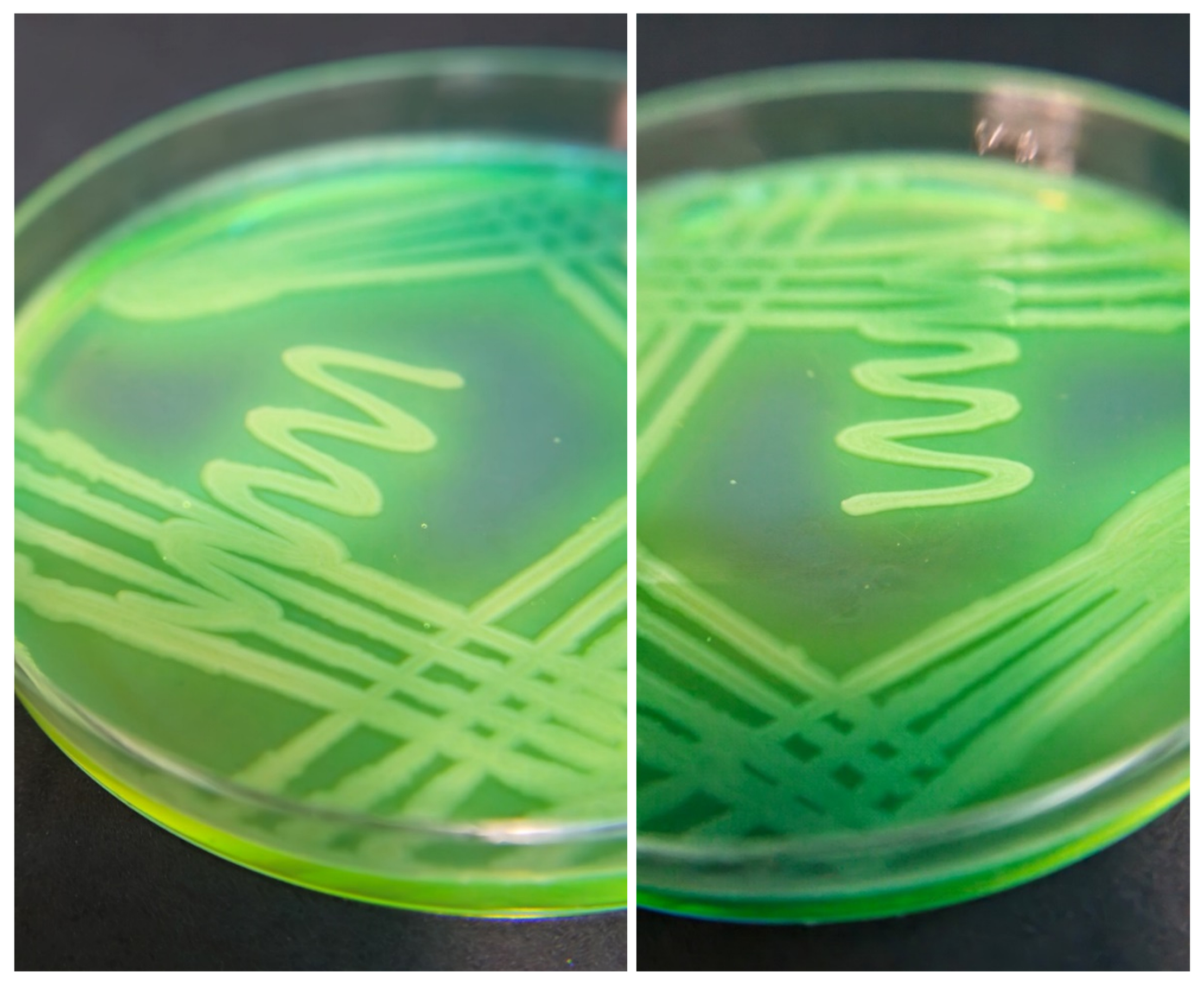

Supplement: Supplementary file 1 [file Data_Sheet_1.zip › latest_supplementary_material file/Supplementary_Figures_TIFF/Supplementary_Figure_S7.tiff]

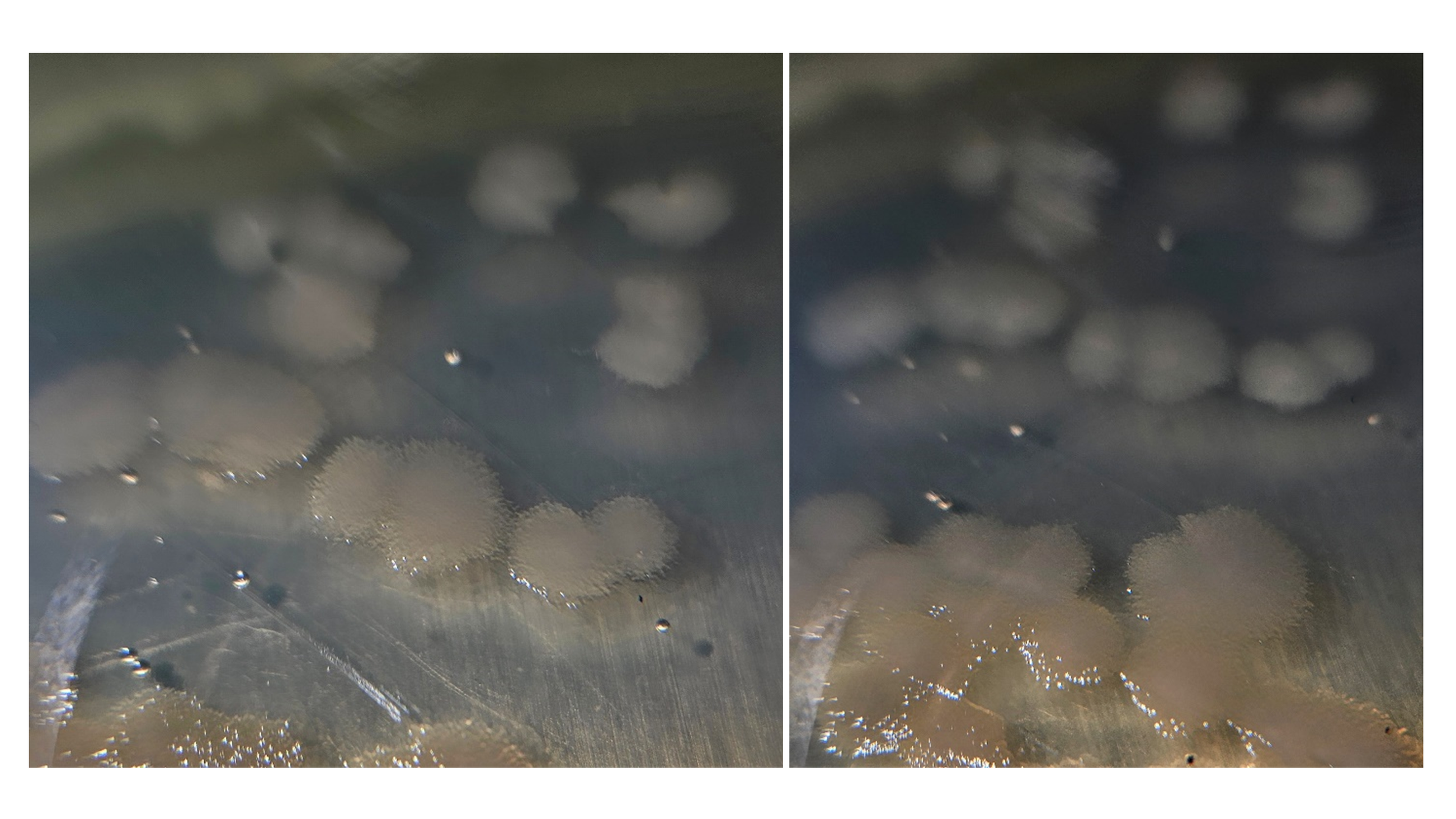

Supplement: Supplementary file 1 [file Data_Sheet_1.zip › latest_supplementary_material file/Supplementary_Figures_TIFF/Supplementary_Figure_S8.tiff]

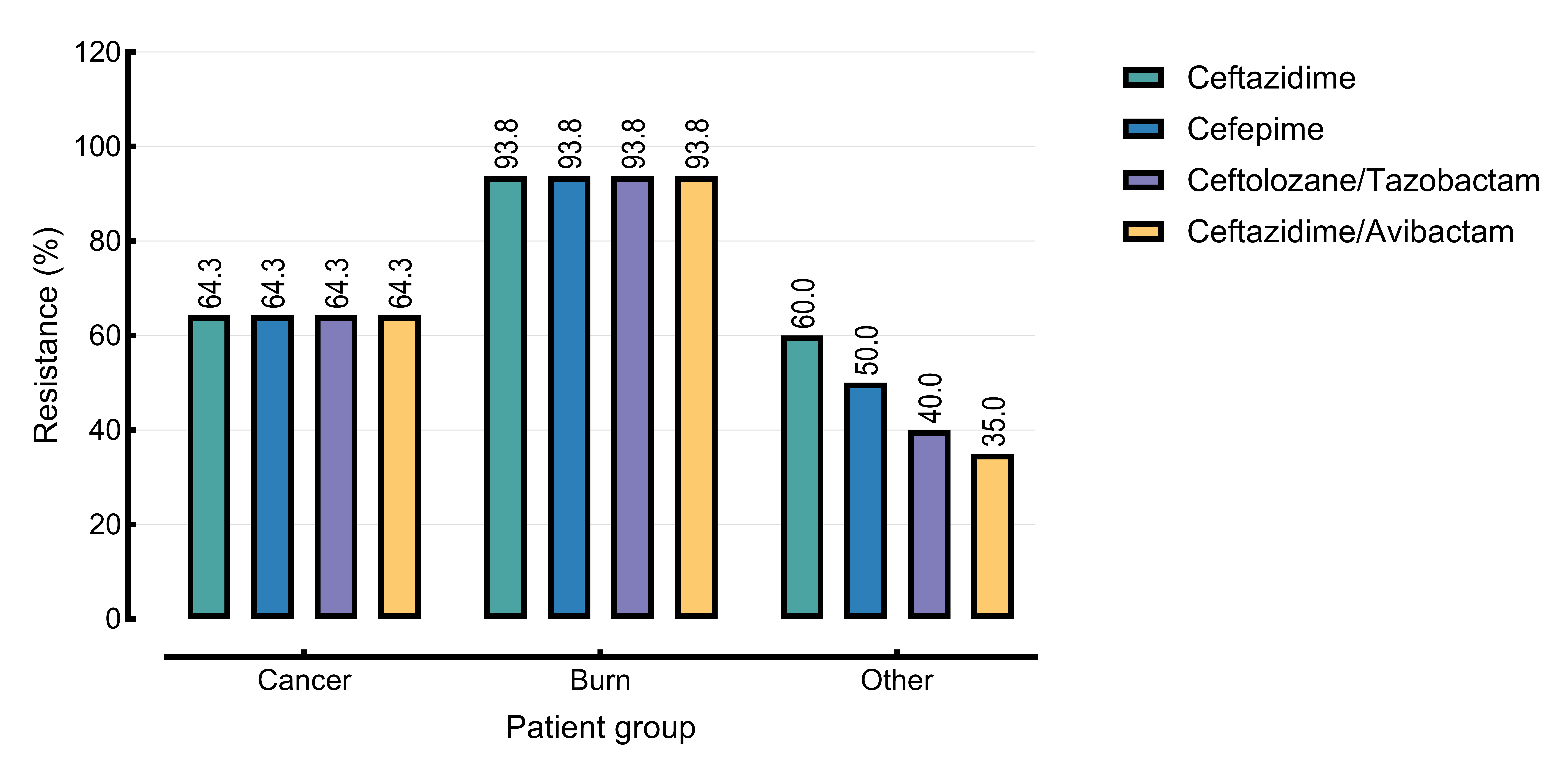

Supplement: Supplementary file 1 [file Data_Sheet_1.zip › latest_supplementary_material file/Supplementary_Figures_TIFF/Supplementary_Figure_S9.tiff]
